# Lipotoxic Injury Differentially Regulates Brain Microvascular Gene Expression in Male Mice

**DOI:** 10.3390/nu12061771

**Published:** 2020-06-13

**Authors:** Saivageethi Nuthikattu, Dragan Milenkovic, John C. Rutledge, Amparo C. Villablanca

**Affiliations:** 1 Division of Cardiovascular Medicine, Department of Internal Medicine, University of California, One Shields Ave., The Grove, Rm 1159, Davis, CA 95616, USA; snuthikattu@ucdavis.edu (S.N.); dragan.milenkovic@inra.fr (D.M.); jcrutledge@ucdavis.edu (J.C.R.); 2 INRA, UNH, Université Clermont Auvergne, 63000 Clermont-Ferrand, France

**Keywords:** genomics, microvascular, brain, dementia, hyperlipidemia, Western diet, males

## Abstract

The Western diet (WD) and hyperlipidemia are risk factors for vascular disease, dementia, and cognitive impairment. However, the molecular mechanisms are poorly understood. This pilot study investigated the genomic pathways by which the WD and hyperlipidemia regulate gene expression in brain microvessels. Five-week-old C57BL/6J wild type (WT) control and low-density lipoprotein receptor deficient (LDL-R−/−) male mice were fed the WD for eight weeks. Differential gene expression, gene networks and pathways, transcription factors, and non-protein coding RNAs were evaluated by a genome-wide microarray and bioinformatics analysis of laser-captured hippocampal microvessels. The WD resulted in the differential expression of 1972 genes. Much of the differentially expressed gene (DEG) was attributable to the differential regulation of cell signaling proteins and their transcription factors, approximately 4% was attributable to the differential expression of miRNAs, and 10% was due to other non-protein coding RNAs, primarily long non-coding RNAs (lncRNAs) and small nucleolar RNAs (snoRNAs) not previously described to be modified by the WD. Lipotoxic injury resulted in complex and multilevel molecular regulation of the hippocampal microvasculature involving transcriptional and post-transcriptional regulation and may provide a molecular basis for a better understanding of hyperlipidemia-associated dementia risk.

## 1. Introduction

Alzheimer’s disease (AD) is a progressive disease characterized by a decline in cognitive function and loss of memory, and its etiology includes both environmental and genetic factors [1]. The strongest genetic risk factor for AD is the ε4 variant of apolipoprotein E (ApoE), yet the most common cause of vascular dementia (VaD) is cerebral small vessel disease [2]. Risk factors for cardiovascular disease (CVD) are known to overlap with risk factors of AD and VaD [1], still the mechanistic links have not been clearly established [3]. As a result, understanding the effect of CVD risk factors in dementia provides potential important therapeutic targets for the prevention of cognitive dysfunction.

The global increase in obesity has been linked to a decline in complex carbohydrate and fiber intake, diets with fewer fruits and vegetables, and a shift towards diets high in fat and refined sugars—otherwise known as the Western-type diet (WD). An association has been reported between obesity and cognitive dysfunction and reduced neural integrity (e.g., grey and white matter atrophy) [4]. One consequence of the WD is hyperlipidemia. A greater risk of AD is correlated with high concentrations of low-density lipoprotein cholesterol (LDL-C) and total cholesterol (TC) [5] and increased consumption of saturated fat is linked to compromised cognitive function, working memory, and attention [6]. We have also recently shown that the WD results in cognitive dysfunction in hyperlipidemic male mice [7].

High dietary fat and cholesterol also have functional sequelae in the brain. The blood–brain barrier (BBB) plays a key role in many cognitive dysfunction and neurodegenerative disorders, presumably leading to increased movement of immune cells and immune intermediaries into the brain, which contribute to neuro-inflammation and consequently, neuro-degeneration [8,9]. The BBB function is regulated by brain endothelial cells, which together with neurons and non-neuronal cells (e.g., pericytes, astrocytes, and microglia), form a functional unit known as the neurovascular unit. Injury of the endothelial cell layer can have adverse consequences on brain function. Our prior work has shown that when the endothelial cell function is disrupted in wild type C57BL/6J mice fed the WD, there is a significant increase in BBB permeability associated with cognitive dysfunction and impaired memory [7]. Further, we have shown that exposure of endothelial cells to triglyceride-rich lipoproteins (TGRL) lipolysis products induces significant endothelial cell injury related to BBB dysfunction [10]. Others have similarly shown that hyperlipidemia induces changes in the expression of the vascular endothelial growth factor and tight junction protein Claudin-5 that affects the permeability of the BBB [11].

In vivo studies have been performed on whole brains or specific regions of the brain, thus obscuring the specific effects of a hyperlipidemic diet on endothelial cells. In addition, it is known that lipids are central to the pathogenesis of dementia, yet the multifactorial mechanisms by which they contribute to cognitive dysfunction in the brain are not entirely understood. Several studies have employed genomic approaches to assess for molecular level effects following the Western-type diet or hyperlipidemia induced by the low-density lipoprotein receptor-deficient (LDLR−/−) genotype. For example, high-fat diets can modulate the expression of genes related to neuronal projections and synaptic transmission corresponding to significant deterioration of neurite morphology and cognition [12]. In addition to changes in the expression of protein-coding genes, diets rich in lipids can modulate the expression of several miRNAs [13].

More recently, it has also been shown that a high-fat diet can also regulate long non-coding RNAs (ncRNAs) [14]. LncRNAs are non-protein-coding transcripts with at least 200 nucleotides in length. They have a broad range of functions in diverse biologic processes, including having a potential role in brain function and disease [15]. LncRNAs have also been found to recruit transcription factors and chromatin-modifying complexes to specific genomic sites, thereby contributing to the transcriptional and epigenetic regulation of gene expression. These studies suggest that lipotoxic injury can induce significant changes at the genome level in the brain. However, these studies were performed on whole brain or brain regions, and the specific response of different cell types, specifically brain microvascular endothelial cells, is not well understood.

LDL-R-deficient mice are widely used as models for the investigation of atherosclerosis and diet-associated lipotoxic injury because the LDL receptor is essential to the removal of ApoE-containing lipoproteins from the blood [16]. Deficiency of LDL receptors extends the residence of LDL in the blood, making LDL-R-deficient mice a particularly useful model for examining the association between lipid metabolism and inflammatory processes [16]. This, in turn, is of importance to understanding neurovascular inflammation and the vascular determinants of dementia. In our recently published work, we used whole genome transcriptomic analysis to study how hyperlipidemia affects the microvasculature in the hippocampus, a key memory center in the brain, in female low-density lipoprotein receptor-deficient (LDL-R−/−) mice fed the Western diet (WD) [17]. We showed for the first time that hyperlipidemic stress modulates the differential expression of the hippocampal microvascular genome in the females by 7%, including for protein-coding and non-coding genes (microRNAs, small nucleolar RNAs, and long non-coding RNAs). These differentially expressed genes (DEGs) were associated with the differential regulation of a number of important cellular pathways such as the regulation of the actin cytoskeleton, cell adhesion amyloid proteins, regulation of angiogenesis, Rap-1 signaling pathway, and transcription factors such as CREB1 (cAMP responsive element binding protein), ESR1 (estrogen receptor 1), and YY1 (Yin Yang 1) [17]. However, how hyperlipidemic stress and the WD affect the entire hippocampal microvascular genome in male mice is currently unknown. As women have more rapid cognitive decline after a diagnosis of dementia than men [18] and these differences do not appear to be solely explainable by the longer life expectancy of women when compared with men, it is important to fully characterize the nutrigenomic response in males to better understand the effect of the WD on the male brain microvasculature. We hypothesized that the WD would induce complex genomic effects that lead to the differential gene expression of previously unreported protein-coding and non-protein-coding genes in the male brain hippocampal microvasculature.

Thus, the goal of our study was to determine and characterize the molecular mechanisms for the genomic effects of a high-fat diet and experimental hyperlipidemia on brain endothelium of LDL-R-deficient male mice, by performing global transcriptomic analyses on laser-captured isolated microvessels from the hippocampal regions of the brain. In addition, most prior studies have evaluated the impact of lipids on one type of RNA but have not ventured beyond that or looked at potential interactions, and therefore, a secondary goal was to perform integrated omics analyses to better understand the complexity of genetic regulation by non-protein-coding RNAs in response to the WD in males.

## 2. Methods

### 2.1. Experimental Animals

Low-density lipoprotein receptor-deficient (LDL-R−/−; strain B6.129S7-Ldlr tm1Her/J, Jackson Laboratories, Bar Harbor, ME, USA) and C57BL/6J wild type (WT; Jackson Laboratories, stock 000664) male mice were fed either a standard chow control diet (CD = Chow, Nestlé Purina PetCare Co., St. Louis, MO, USA) or a Western diet (WD, catalog no. 88137, Harlan Laboratories, Madison, WI, USA) composed of 21% fat and 0.2% cholesterol (*w*/*w*) for 8 weeks. There were four experimental treatment groups randomly assigned to the diets: WT fed CD = WT-CD, WT fed WD = WT-WD, LDL-R −/− fed CD = LR-CD, and LDL-R fed WD = LR-WD; n = 7 mice/group. Animals were housed in single cages in a temperature- and humidity-controlled environment with a 12 h light/dark cycle in the University of California, Davis Mouse Biology Program. Body weight was measured at baseline and at the completion of the dietary intervention period, and activity and food intake monitored daily by vivarium staff. Research was conducted in conformity with the Public Health Service Policy on Humane Care and Use of Laboratory Animals. The institutional review board of the University of California, Davis, the Institutional Animal Care and Use Committee (IACUC) approved this project protocol number 19750 on 7 February 2017.

### 2.2. Blood Metabolic and Hormone Assays

Fasting lipid levels were measured in serum samples that were stored at −80 °C until assayed. Triglyceride (TG), total cholesterol (TC), high-density lipoprotein cholesterol (HDL), and low-density lipoprotein cholesterol (LDL) were measured using enzymatic assays from Fisher Diagnostics (Middleton, VA, USA), and precipitation separation from AbCam (Cambridge, MA, USA) adapted to a microplate format. Fasting glucose and insulin levels were also measured on serum samples. Glucose was measured using enzymatic assays from Fisher Diagnostics (Middleton, VA, USA), and insulin was determined by electrochemiluminescence from Meso Scale Discovery (Rockville, MD, USA) according to the manufacturer’s instructions. All assays were performed by the UC Davis Mouse Metabolic Phenotyping Center (MMPC) in triplicate, on non-pooled plasma samples.

### 2.3. Isolation and Cryosection of Murine Brain Hippocampus

Following completion of the dietary feeding period, mice were anesthetized by intraperitoneal xylazine/ketamine and euthanized by exsanguination during the light phase of their light/dark cycle, then intravascularly perfused with DEPC-treated PBS. Intact brains were rapidly removed under RNAse free conditions, cut into regions including the temporal lobe segment, and embedded using HistoPrep Frozen Tissue Embedding Media (Fisher Scientific, Pittsburgh, PA, USA). To identify the hippocampus and hippocampal neurons, brain sections in the medial aspect of the temporal lobe were stained with hematoxylin and visualized with microscopy by a histopathology expert at UC Davis (Dr. Dennis Wilson). The hippocampus was then coronally cryosectioned (8 µm, Leica Frigocut 2800 n Cryostat, Leica Biosystems, Buffalo Grove, IL, USA). Hippocampal cryosections were placed on charged RNA-free PEN membrane glass slides, treated with RNA*later*^®^-ICE (Life Technologies, Grand Island, NY, USA) to prevent RNA degradation, and stored at −80 °C until use. When ready for use, cryosections from the hippocampal segments were submerged in nuclease-free water and dehydrated in desiccant.

### 2.4. Laser Capture Microdissection (LCM) of Hippocampal Microvessels

For the analysis of the gene transcriptome of the hippocampal brain microvessels, endothelial microvessels (<20µm) were first identified in the hippocampal brain cryosections by alkaline phosphatase staining utilizing 5-bromo-4-chloro-3-indolyl phosphate/nitro blue tetrazolium chloride (BCIP/NBT) substrate as previously described [19]. Laser capture microdissection (LCM) was then used to isolate the endothelium of the microvessels within the hippocampal sections by capture of the entire vessel wall under direct microscopic visualization using a Leica LMD6000 Laser Microdissection Microscope (Leica Microsystems, Wetzlar, Germany), Supplemental Figure S1. Microvessels were not categorized by the hippocampal region or subregion, although they primarily corresponded to endothelial-enriched sections in the hippocampus dorsal segments that would have included the CA1 and CA3 regions.

### 2.5. RNA Extraction from Laser Captured Brain Microvessels

Total RNA was extracted from the laser-captured hippocampal brain microvessels (100 microvessels/sample) from each of the four experimental animal groups using an Arcturus PicoPure™ RNA Isolation Kit (Thermo Fisher Scientific, Santa Clara, CA, USA) according to the manufacturer’s instructions. The quality of the RNA from the LCM-derived vessels was assessed by Nanodrop, and RNA integrity verified by qRT-PCR of the control gene transcription (GAPDH). RNA quantification was performed according to Affymetrix RNA quantification kit with the SYBR Green I and ROX™ Passive Reference Dye protocol (Affymetrix, Santa Clara, CA, USA).

### 2.6. Microarray Hybridization and Transcriptome Analysis

For the transcriptomics analysis, we used Affymetrix GeneChip Mouse Gene 2.0 ST Array (~28,000 coding transcripts and ~7000 non-coding transcripts, Affymetrix, Santa Clara, CA, USA). RNA (125 pg) was used to prepare cRNA and sscDNA using an Affymetrix GeneChip ^®^ WT Pico Kit. SscDNA (5.5 µg) was fragmented by uracil-DNA glycosylase (UDG) and apurinic/apyrimidinic endonuclease 1 (APE 1) and labeled by terminal deoxynucleotidyl transferase (TdT) using the DNA labeling reagent that is covalently linked to biotin. Fragmented and labelled ssCDNA samples in triplicate were then submitted to the UC Davis Genome Center shared resource core for hybridization, staining, and scanning using the Affymetrix WT array hybridization protocol following the manufacturer’s protocol. Hybridization of the fragmented and labelled ssCDNA samples was done using GeneChip ™Hybridization Oven 645, and samples then washed and stained using GeneChip ™ Fluidics Station 450. The arrays were scanned using a GeneChip™ Scanner 3000 7G (Thermo Fisher Scientific, Santa Clara, CA, USA). Quality control of the microarrays was done using the Affymetrix Expression Console software version 1.4.1 and data analysis performed using the Affymetrix Transcriptome Analysis Console software version 3.1.0.5.

### 2.7. qRT-PCR Analysis of Gene Expression in Murine Hippocampal Microvessels

To corroborate the microarray analysis results, we performed qRT-PCR on 11 randomly selected differentially expressed RNA transcripts. For these experiments, RNA (75 ng) from the laser-captured brain microvessels was reverse-transcribed into cDNA using iScript Reverse Transcription Supermix for RT-Qpcr (Biorad, Hercules, CA, USA). qRT-PCR for selected genes was performed in the ABI Vii7 Sequence detection system (PE Applied Biosystems, Foster City, CA, USA). Reactions were carried out in 384-well optical plates containing 25 ng cDNA/well and SsoAdvanced™ Universal SYBR ^®^ Green Supermix as the fluorescent reporter (Biorad, Hercules, CA, USA). Specific primers were designed with the Primer3 software [20] using the gene sequences obtained from the Affymetrix transcript IDs. The sequences of the primers used are listed in the Table S1. The PCR amplification parameters were as follows: initial denaturation step at 95 °C for 10 min followed by 40 cycles, each at 95 °C for 15 s (melting) and 60 °C for 1 min (annealing and extension). Gene expression was normalized to the glyceraldehyde-3-phosphate dehydrogenase (GAPDH) transcription. Relative gene expression was calculated using the delta–delta comparative threshold cycle (Ct) method and expressed as a fold change compared with the wild type (WT) mice fed with the control diet (CD).

### 2.8. Bioinformatic Analysis

Bioinformatics analysis of differentially expressed genes was performed by two of the study investigators (SN and DM) using multiple software tools. We compared each study group (LR-WD, LR-CD, and WT-WD) to the control (WT-CD). For the fold change calculations, it was also necessary to input the experimental group data and compare them to the control group data. This information is required by the microarray software (Affymetrix Transcriptome Analysis Console, version 3.1.0.5, Santa Clara, CA) used in the project.

Gene ontology of identified differentially expressed genes was done using the David bioinformatics database (https://david.ncifcrf.gov/home.jsp) [21,22], and a treemap was constructed using Revigo (http://revigo.irb.hr/) [23]. Canonical pathway analysis was conducted using the GeneTrial2 online database (https://genetrail2bioinfuni-sbde) [24] and the Metacore software package (https://portalgenegocom) to identify significantly over represented pathways. Enrichment statistics were calculated for these data sets assuming a hypergeometric distribution to identify significantly over represented pathways. Gene network and transcription factor analyses were performed using Metacore™. MicroRNA validated targets were searched using the miRWalk database (http://zmf.umm.uni-heidelberg.de/apps/zmf/mirwalk2/index.html) [25] that enables the retrieval of experimentally verified miRNA–gene target interactions. Hierarchical clustering and heat map representations of miRNA profiles were performed using the PermutMatrix software (http://www.atgc-montpellier.fr/permutmatrix/) [26]. Venn diagrams were generated using Venny (http://bioinfogp.cnb.csic.es/tools/venny/). Network analysis of interactions between functional groups was identified using Metascape (http://metascape.org/) [27], and obtained networks were visualized using the Cytoscape platform (https://cytoscape.org/) [28]. OmicsNet from MetaboAnalyst (http://wwwmetaboanalystca/MetaboAnalyst/faces/homexhtml) [29] was used for the integrated analyses of the protein–protein network with miRNAs and transcription factors. To identify potential target genes and miRNAs of lncRNAs, we used several databases, including starBase (http://starbase.sysu.edu.cn/index.php) [30], starBase v2.0: decoding miRNA–mRNA, miRNA–ncRNA and protein–RNA interaction networks from large-scale CLIP-Seq data; miRcode (http://www.mircode.org/index.php) [31], and the RNAcentral database (https://rnacentral.org) [32].

### 2.9. Statistical Methods

For the microarray, a two-way ANOVA (Affymetrix Transcriptome Analysis Console software, Santa Clara, CA) was used for the statistical analysis of the microvessel transcriptome of the WT-WD, LR-CD mice, and LR-WD, each compared with the WT-CD mice. All genes from the microarray with *p* < 0.05 and a ±2.0-fold change were considered as significantly differentially expressed. Mean body weight and plasma lipid levels of all diet/genotype groups (WT-CD, WT-WD, LR-CD, LR-WD) were expressed as means ± standard error of the mean (SEM), and significance was determined at *p* ≤ 0.05 using unpaired student’s *t*-tests (GraphPad software, La Jolla, CA, USA). qRT-PCR determined the gene expression in the hippocampal microvessels of the experimental mice, compared with WT-CD, and was expressed as a log2-fold change, and statistical significance was determined by unpaired student’s *t*-tests (GraphPad software, La Jolla, CA, USA).

## 3. Results

### 3.1. Model of Hyperlipidemia

The dietary treatment resulted in the expected weight gain in the study mice as follows: mean weight for WT mice at baseline was 21 g and increased by an average of 24% when fed with CD and 39% when fed with WD; mean weight of LR mice at baseline was 17.25 g and increased by an average of 62% when fed with CD and 74% when fed with WD (*p* < 0.05 respectively for all group comparisons): Supplemental Figure S2.

After eight weeks on the experimental diets, the mean total cholesterol levels in the CD- and WD-fed WT mice were 89.3 and 252.8 mg/dL (*p* < 0.05), respectively, and 285.6 and 1151.8 mg/dL (*p* < 0.05) for the LR-CD and LR-WD-fed mice, respectively: Supplemental Table S2A. We also determined the blood glucose and insulin levels in our study mice. Compared with WT-CD, glucose levels were highest and significantly greater (*p* < 0.05) in the LR-WD-fed groups: Supplemental Table S2B. Insulin levels were highest and significantly greater (*p* < 0.05) in the LDL-R−/− genotype and WD-fed mice compared with the WT mice on the CD. These results are consistent with what has been published previously for these experimental models [33,34].

### 3.2. Effect of the Western Diet on Brain Hippocampal Microvessel Gene Expression

To define the molecular mechanisms in the brain hippocampal microvessels in response to the WD, we began by assessing the effect of the WD on the global gene expression in the hippocampal microvessels of the CD-fed and WD-fed WT and LDL-R−/− male mice. These studies showed that among the 34,472 genes studied in the microarrays, 1972 genes (5.7%) were differentially expressed (DE). Volcano plots of the significantly up- or down-regulated genes in the brain microvessels of WT-WD showed up-regulation of two genes and down-regulation of eight genes compared with the microvessels of the WT-CD mice: Supplemental Figure S3A (see Supplemental Table S3 for a complete listing of the DE genes). The effect of the WD was contrasted to the effect of genotype by comparing the differential gene expression in LR-CD to WT-CD, which revealed up-regulation of 961 genes and down regulation of 2 genes: Supplemental Figure S3B (see Supplemental Table S4 for a complete listing of the DE genes). In contrast, in the microvessels of LR-WD, there was up-regulation of 1012 genes and down-regulation of 5 genes compared with the WT-CD mice: Supplemental Figure S3C (see Supplemental Table S5 for a complete listing of the DE genes). Although over 85% of the differential gene expression in the brain microvessels of male mice was for protein-coding genes, to our knowledge, we show for the first time that the WD also regulated the expression of protein-non-coding genes in the male brain microvessels: long non-coding RNAs (lncRNA), microRNAs (miRNAs), and small nucleolar RNAs (snoRNAs). The total number of differentially expressed non-protein-coding genes was greatest (153 total) for LR-WD, and for lncRNAs (113) compared with the other ncRNA (83 miRNAs and 97 snoRNAs).

**Figure 1:**
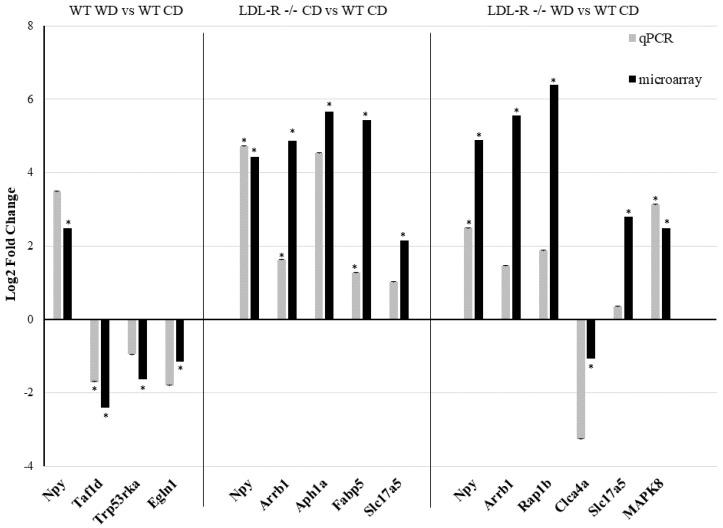
Gene expression by qRT-PCR of genes identified by microarray analysis in hippocampal microvessels. Eleven genes (Npy, Taf1d, Trp53rka, Egln1, Arrb1, Aph1a, Fabp5, Slc17a5, Rap1b, Clca4a, MAPK8) were tested by qRT-PCR in the hippocampal microvessels isolated from wild type (WT) and LDL-R−/− mice fed with the control diet (CD) and Western diet (WD) and showed the same trend in gene expression as the microarray. Expression levels were expressed as a log2-fold change (* *p* ≤ 0.05 for WT-WD, LDL-R−/− CD, and LDL-R−/− WD when compared with WT-CD).

A random sample of eleven differentially expressed protein-coding and ncRNA, representative of each of the experimental genotype/diet groups, was tested by qRT-PCR and confirmed to have the same direction of change in gene expression (up- or down-regulation) as observed with the microarrays (Figure 1). These data suggest a significant effect of the consumption of the WD on the differential gene expression in brain microvessels, attributable to both the WD and the LR genotype.

### 3.3. Effect of the Western Diet on Expression of Protein-coding Genes in Brain Hippocampal Microvessels

To identify the biological functions of differentially expressed protein-coding genes, and consequently the potential cellular processes which could be affected by changes in their expression, we performed a series of bioinformatic analyses. Our first analysis was to identify the gene ontologies of the differentially expressed genes. The genes identified as differentially expressed are involved in many biological functions: Supplemental Figure S4. These functions include angiogenesis (cellular response to insulin stimulus, triglyceride metabolism regulation of reactive oxygen species, or G protein-coupled receptor internalization), apoptotic processes (leukocyte chemotaxis, regulation of vasodilation, regulation of endothelial cell migration, angiogenesis), gene regulation and RNA biogenesis (mRNA splicing via spliceosomes, ubiquitin-dependent protein catabolism, regulation of Nf-kB transcription factor activity), and cell adhesion. This observation suggests that the cellular functions primarily affected by lipid-associated vascular injury are involved in angiogenesis, apoptosis, immune cell interaction, and gene regulation and RNA biogenesis.

**Figure 2:**
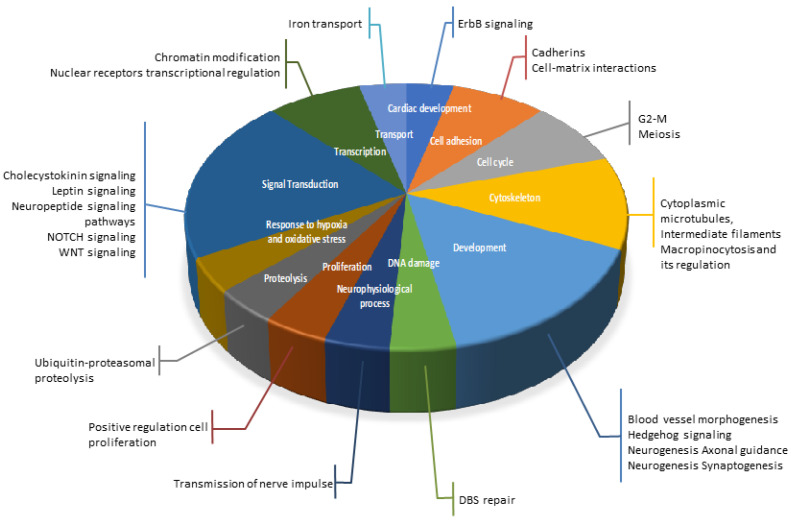
Gene Network Analysis of differentially expressed protein-coding genes in hippocampal microvessels. A text-mining algorithm (Metacore) was used to perform the gene network analyses and identify functional groups of the differentially expressed protein-coding genes in the hippocampus microvessels from Western diet (WD)-fed C57BL/6J (WT) mice compared with control diet (CD)-fed WT mice, CD-fed LDL-R−/− mice compared with CD-fed WT mice, and WD-fed LDL-R −/− mice compared with CD-fed WT mice. These gene networks represented in the pie chart were involved in cell adhesion (orange), cytoskeleton (yellow), proliferation (brown), proteolysis (grey), transcription (dark green), development (light blue), and signal transduction (dark blue).

Next, we performed gene network analyses using a text-mining algorithm (MetaCore) to identify the functional groups of the gene networks (Figure 2). Using this approach, we identified gene networks involved in cell adhesion (cell–matrix interactions and cadherins), cytoskeleton (cytoplasmic microtubules, intermediate filaments, macropinocytosis and its regulation), proliferation, proteolysis, transcription (nuclear receptors transcriptional regulation, chromatin modification), development, and signal transduction (Wingless and Int-1 (WNT) signaling, Notch signaling or neuropeptide signaling pathways). The gene network analysis suggests that lipid injury can principally affect the expression of genes involved in cellular network processes that participate in cell adhesion, development, or signal transduction. 

**Figure 3:**
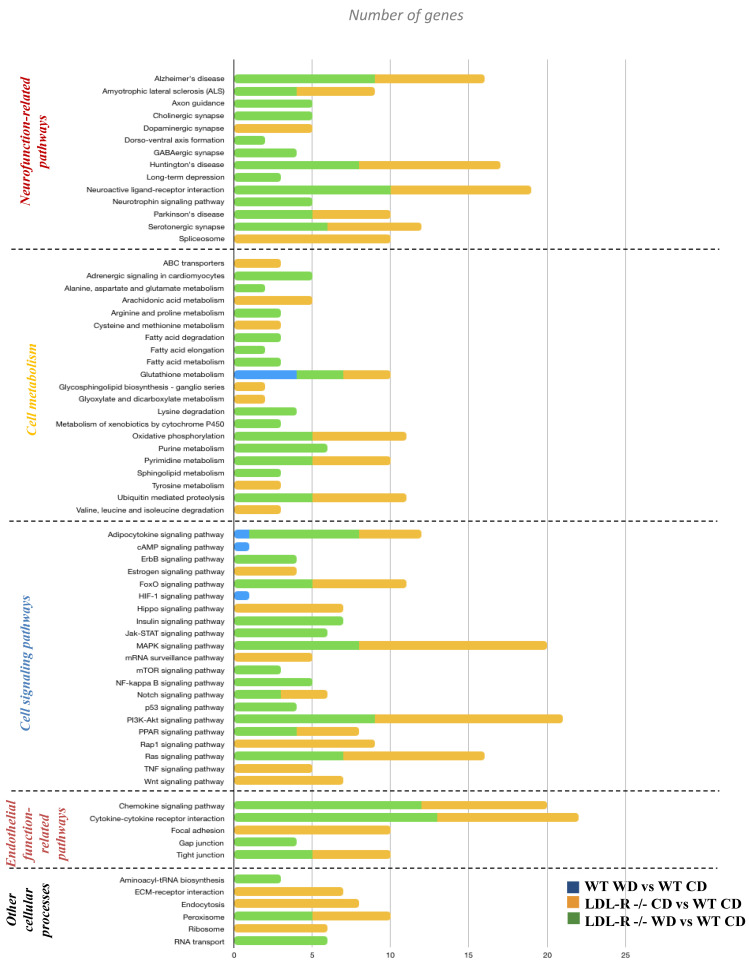
Pathway analyses of the pathways of differentially expressed protein-coding genes in hippocampal microvessels. Histogram of the significant cellular pathways of differentially expressed protein-coding genes in hippocampal microvessels. Cellular pathways of differentially expressed genes in hippocampus microvessels from Western diet (WD)-fed C57BL/6J (WT) mice compared with control diet (CD)-fed WT mice, CD-fed LDL-R−/− mice compared with CD-fed WT mice, and WD-fed LDL-R−/− mice compared with CD-fed WT mice were identified using KEGG and MetaCore.

Following the gene network analyses, we used KEGG and MetaCore to identify the cellular pathways for the differentially expressed genes. We observed the differential regulation of a number of important cellular pathways including those for neurological function-related pathways (axon guidance, long-term depression, or neuroactive ligand–receptor interaction), cellular metabolism (fatty acid metabolism, ABC transporters, glutathione metabolism, or oxidative phosphorylation), cell signaling (Nf-kB signaling, p53 signaling, Raps1/Ras signaling, insulin signaling, cAMP signaling, or MAPK signaling pathways), endothelial function (focal adhesion, gap junction, or tight junction), and a few other cellular processes (Figure 3). In general, when compared with the WT-CD mice, the LDL-R−/− genotype showed a larger number of genes involved in the differential expression of cellular pathways. The gene network analyses showed a similar trend to the pathway analyses for the LDL-R−/− genotype.

Using the ClueGo tool on Cytoscape, we also searched cellular pathways that form networks to identify the function of the groups of pathways of differentially expressed genes. Using this approach, we identified over 50 pathways in a network. These networks of pathways formed functional groups involved in cell signaling, oxidative stress, endothelial cell function, and neurofunction.

### 3.4. Potential Transcription Factors Involved in the Genomic Effects of the Western Diet on Brain Hippocampal Microvessels

**Figure 4:**
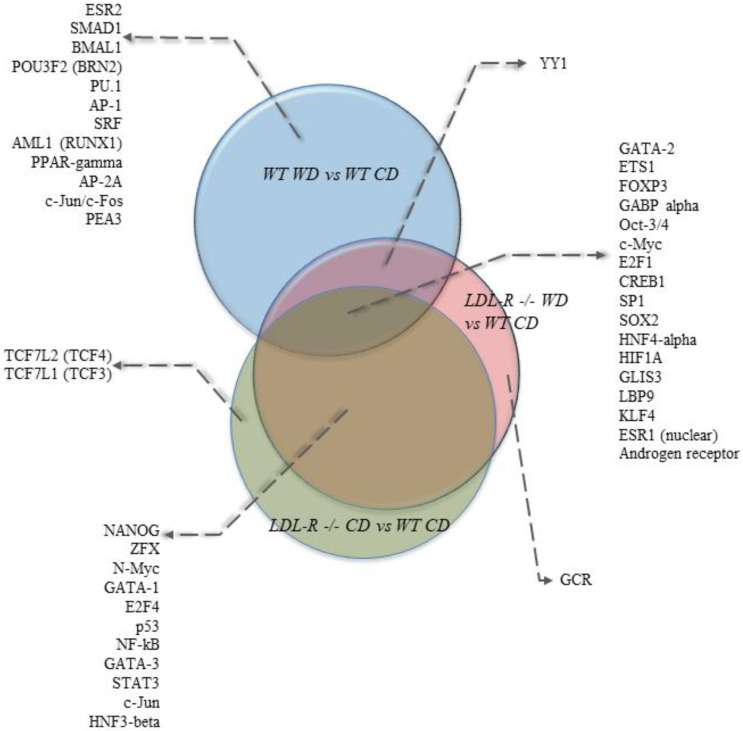
Venn diagram of the top 30 transcription factors affected by diet and genotype in hippocampal microvascular endothelium. Transcription factors potentially modulated by lipid injury were identified using a MetaCore transcription regulation algorithm. Venn diagram shows 17 transcription factors (TFs) in common for the Western diet (WD)-fed C57BL/6J (WT) mice, control diet (CD)-fed LDL-R−/− mice, and WD-fed LDL-R−/− mice, when compared with the CD-fed WT mice.

We also performed bioinformatics analyses of the gene expression data to identify potential transcription factors (TF) whose activity could be modulated by lipid injury and be involved in mediating the observed genomic effects. The most statistically significant transcription factors were ETS1 (ETS proto-oncogene 1), c-Myc (cellular myelocytomatosis), FOXP3 (forkhead box P3), and GABPalpha (GA binding protein transcription factor) (Supplemental Table S6). Comparisons of the top 30 transcription factors (TFs) identified in our study groups are shown in Venn diagrams in Figure 4. Among the top TFs, 17 were in common for the 3 diet and genotype groups. Eleven TFs were in common between the LDLR−/− mice on the CD and the LDLR−/− mice on the WD compared with the WT mice on the CD, while no common TFs were identified between LDLR−/− on the CD and WT on the WD. One TF (YY1) was in common between LDLR−/− on the WD and WT on the WD.

### 3.5. Impact of the Western Diet on Expression of miRNA, their Targets and Pathways in Brain Microvessels

Our microarray analysis allowed us to study not only protein-coding but also miRNAs. Using this approach, we identified that lipotoxic injury can modulate the expression of miRNAs in brain endothelial cells (Supplemental Table S7). We identified that the WD in WT mice increased the expression of one miRNA, miR-1954. On the other hand, lipotoxic injury induced by the LDL-R−/− genotype on the WD resulted in the increased expression of 42 miRNAs, including miR692, miR-196a, miR-210 or miR-486, and 39 miRNAs. In comparing the differentially expressed miRNAs for the three study conditions, only one miRNA was in common, miR-1954. On the other hand, 15 miRNAs were identified in common between the LDLR −/− mice on the CD and the WD when compared with the WT mice, including miR-375, miR-210, or miR-678.

To identify potential target genes for the differentially expressed miRNAs in the study groups, we used the miRWalk database (Supplemental Figure S5A). When compared with the WT-CD mice, the miRNA target gene analysis identified 554 differentially expressed gene targets for miRNA in WT-WD, 6844 gene targets for miRNA in LR-CD, and 6153 gene targets for miRNA in LR-WD. The comparison of the target genes of the differentially expressed miRNAs revealed relatively little overlap (a total of 1 gene, 269 genes, and 189 genes, in common for WT-WD, LR-CD, and LR-WD, respectively, when compared with the WT-CD mice).

Our next step was to identify the pathways of the target genes of the differentially expressed miRNAs. We used the miRWalk online tool to search for the pathways in the KEGG database. Using this approach, we showed that compared with the WT-CD mice, there were 6, 68, and 25 pathways of miRNA target genes in the WT-WD, LR-CD, and WD-fed LDL-R−/− mice, respectively (Supplemental Figure S5B). Among the pathways were those involved in the regulation of cGMP-PKG signaling, Ras/Rap signaling, regulation of the actin cytoskeleton, chemokine signaling, insulin resistance, PI3K-Akt signaling, focal adhesion, cytokine–cytokine receptor interaction, and the NF-kappa B signaling pathway.

**Figure 5:**
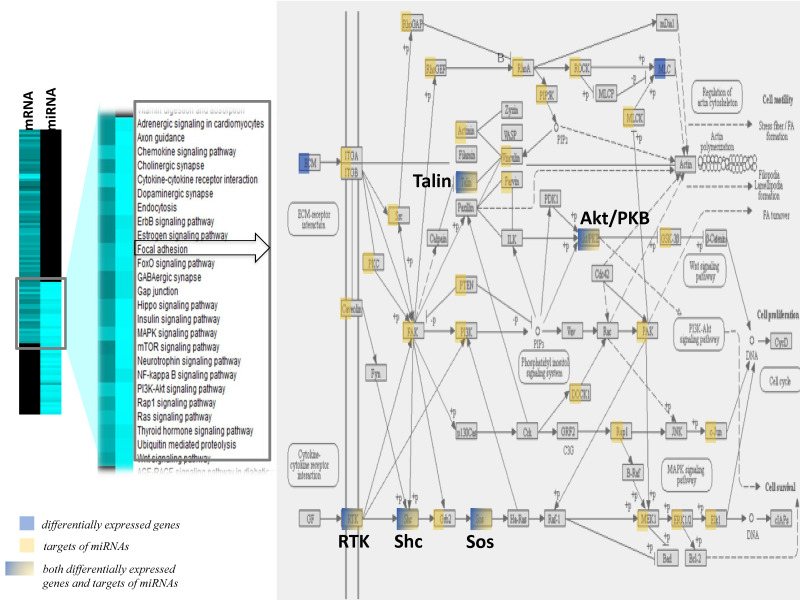
Effect of lipid injury on microRNA target differentially expressed gene pathways expression in hippocampal microvessels. Heat map of differentially expressed gene pathways and miRNA target gene pathways. Comparisons of pathways of differentially expressed genes and pathways of miRNA target genes identified a group of pathways such as the chemokine signaling pathway, focal adhesion, gap junction, insulin signaling, Nf-kB signaling or Gap junctions, and pathways that regulate endothelial cell interaction and permeability in common. The representative integrated analysis of the differentially expressed genes, and target genes of differentially expressed miRNAs for the focal adhesion signaling pathway is detailed. Blue = differentially expressed genes; yellow = target genes of differentially expressed miRNAs; color gradation from blue to yellow = genes identified to be both differentially expressed and to be targets of differentially expressed miRNAs.

The comparisons of the pathways identified with the differentially expressed genes and pathways identified using the target genes of miRNAs revealed a group of pathways in common between the two modes of gene regulation (Figure 5). Among the pathways in common were the chemokine signaling pathway, focal adhesion, gap junction, insulin signaling, Nf-kB signaling or Gap junctions, and pathways that regulate endothelial cell interaction and permeability. The integrated analyses of focal adhesion or the Rap1 signaling pathway showed that most of the genes were either differentially expressed following lipotoxic injury or were potential target genes of differentially expressed miRNAs. This observation suggests a potential dual mode of action of lipotoxic injury in brain endothelial cells—both at the transcriptional and post-transcriptional levels of regulation.

**Figure 6:**
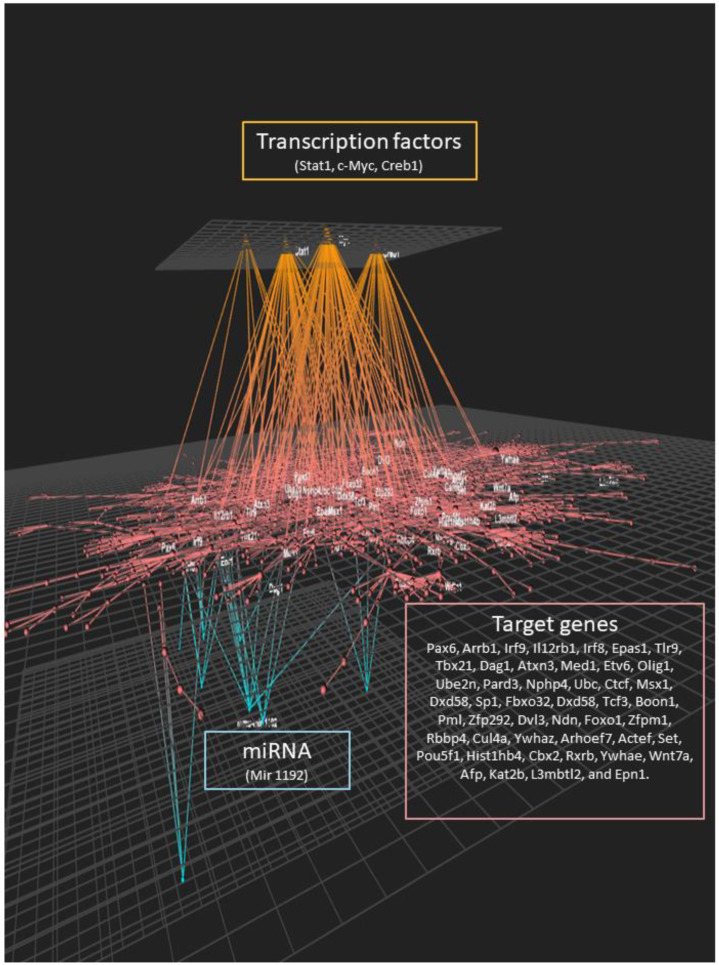
Protein–protein network enriched with transcription factors and miRNA regulation. Protein–protein interactions were identified using the STRING database from differentially expressed genes. Interactions between the protein–protein network, differentially expressed miRNAs (Mir 1192), and potential transcription factors (Stat1, c-Myc, Creb1) were constructed using the OmicsNet online tool. Target genes are: Pax6, Arrb1, Irf9, Il12rb1, Irf8, Epas1, Tlr9, Tbx21, Dag1, Atxn3, Med1, Etv6, Olig1, Ube2n, Pard3, Nphp4, Ubc, Ctcf, Msx1, Dxd58, Sp1, Fbxo32, Dxd58, Tcf3, Boon1, Pml, Zfp292, Dvl3, Ndn, Foxo1, Zfpm1, Rbbp4, Cul4a, Ywhaz, Arhoef7, Actef, Set, Pou5f1, Hist1hb4, Cbx2, Rxrb, Ywhae, Wnt7a, Afp, Kat2b, L3mbtl2, and Epn1.

We used the OmicsNet tool to incorporate the data obtained by the individual analyses and integrate it with the data obtained on the effect of lipotoxic injury on the expression of protein-coding genes, potential transcription factors involved in the observed genomic response, and potential post-transcriptional regulation of gene expression (Figure 6). We observed a large inter-connecting network between the three levels of genomic regulation of cell function. The transcription factors identified were associated with a large number of genes identified as differentially expressed. These genes were also connected to the expression of miRNAs identified as modulated by lipotoxic injury. Taken together, this analysis suggests a very complex and multilevel genomic effect of lipotoxic injury on hippocampal microvascular endothelial cells.

### 3.6. Impact of the Western Diet on Expression of snoRNAs and lncRNAs in Brain Microvessels

As detailed above, most of the differential gene expression in our study system was attributable to the differential regulation of cell signaling proteins and their transcription factors. However, approximately 4% of the differential expression was attributable to the differential expression of miRNAs, and 10% was due to other ncRNA, primarily long non-coding RNAs (lncRNAs) and small nucleolar RNAs (snoRNAs).

Regarding non-coding RNAs, we identified the differential expression of a total of 109 lncRNAs and 97 snoRNAs in the experimental groups when compared with the control WT-CD mice: Table 1. The differential expression for snoRNAs and lncRNAs consisted almost exclusively of their up-regulation, and only a few snoRNAs, like Snord61, Gm25443, and Snord61, were identified as down-regulated. This analysis shows for the first time that lipotoxic injury modulates the expression of ncRNA in the brain microvasculature in vivo, revealing a new mode of biological regulation via snoRNAs and lncRNAs.

### 3.7. Integration of Multiomics Data

**Figure 7:**
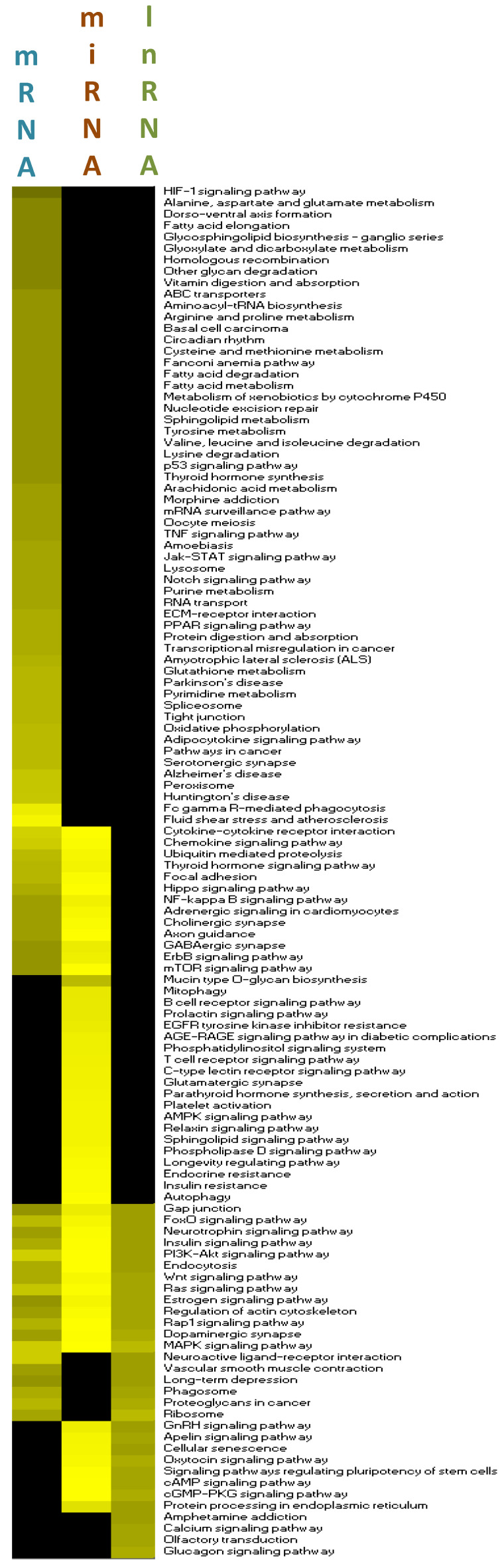
Heatmap of pathway analyses of differentially expressed genes, miRNA target genes, and lncRNA targets. Heat map of the pathways identified using the KEGG database using differentially expressed genes, target genes of differentially expressed miRNA, and targets of lncRNA. The color intensity is proportional to the number of genes in the pathway.

**Figure 8:**
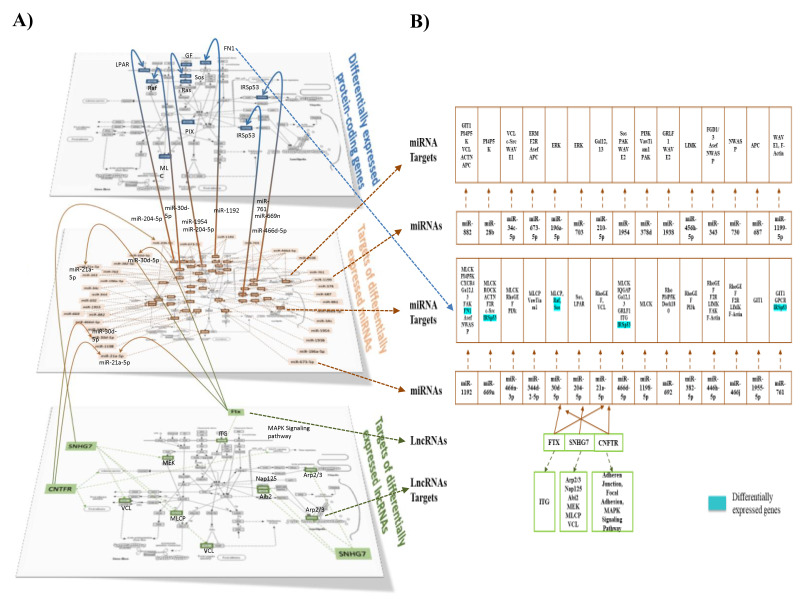
Regulation of the actin cytoskeleton pathway by differentially expressed genes, miRNA, and lncRNA. **(A)** Integration analysis showing differentially expressed genes (blue squares), differentially expressed miRNAs (brown squares) and their targets (brown broken arrows), and differentially expressed lncRNAs (green squares) and their targets (green broken arrows). Potential interactions between genes, miRNA, and lncRNAs are also shown. (**B**) Amplification of the list of differentially expressed genes (highlighted in blue), miRNAs and their targets, and lncRNAs and their targets, regulating the actin cytoskeleton pathway.

The next step in our bioinformatic analyses was to integrate the different omic analyses together. Using a heat map tool, we first compared the pathways identified from the three omic analyses, i.e., from differentially expressed protein-coding genes, targets of differentially expressed miRNAs, and targets of lncRNAs (Figure 7). Using this approach, we identified a set of 12 pathways in common for the three analyses. Among these pathways was the pathway involved in the regulation of cytoskeletal organization. In Figure 8A,B, we present differentially expressed genes implicated in cytoskeletal organization (shown in detail in Supplemental Figure S6A), miRNAs with identified target genes in cytoskeletal organization (shown in detail in Supplemental Figure S6B), as well as lncRNA in this pathway with their target genes (shown in detail in Supplemental Figure S6C). We also identified that differentially expressed lncRNAs can regulate differentially expressed miRNAs. In addition, certain target genes of differentially expressed genes were identified to themselves be differentially expressed. Using the pathway of cytoskeletal organization as an example, these observations exemplify the interactions between the different modes of genomic regulation and indicate that lipotoxic injury can modulate the expression of genes involved in cytoskeletal organization at the level of transcription, but also post-transcription via miRNAs and lncRNAs.

## 4. Discussion

This pilot study was a large-scale transcriptome gene-profiling of the male mice hippocampal microvasculature in relation to lipotoxic injury from two different sources, diet and genetics, using the Western diet (WD) and/or LDL-R−/− (LR) genotype, respectively. Our study focused on the molecular mechanisms of differential gene expression in the brain microvessels due to their significance in the pathogenesis of vascular dementia, and also due to our recently published work demonstrating that the WD results in increased blood–brain barrier (BBB) permeability and cognitive impairment [7], providing a functional correlation to the molecular pathways indicated in this experimental study. Although we describe hippocampal injury induced by the WD and the LR mouse as lipotoxic injury, we cannot omit potential glycemic injury associated with the WD and further studies are needed to make this distinction.

Our main findings are as follows:

➢ The WD resulted in the differential expression (primarily up-regulation) of a large number of genes (1972), representing 5.7% of the genome of microvessels in the hippocampus of male mice;
➢ Overall, the differential gene expression was associated with the differential regulation of cell signaling proteins and their transcription factors, with complex mechanisms of action for genes that regulate increased endothelial dysfunction following lipid stress as the main disruption, particularly via pathways that serve to increase permeability, consistent with the previously reported increase in BBB permeability following the WD;
➢ There were some differences in the differential gene expression for diet and genotype. Differentially expressed genes involved in focal adhesion, ECM–receptor interaction, and signaling pathways (such as PI3K-Akt, TNF, Jak-STAT, and Ras) were up-regulated with lipid injury in the LR genotype while down-regulated by the WD in the wild type mice;
➢ Most of the differential gene expression was attributable to protein-coding genes (85%), but approximately 4% was due to the differential expression of miRNAs, and 10% was due to other non-protein-coding RNAs not previously found to be affected by the WD, including mostly long non-coding RNAs (lncRNAs) and small nucleolar RNAs (snoRNAs). The targets of lncRNAs included genes involved in NF-kB signaling, Ras/Rap signaling, focal adhesion, actin cytoskeleton organization, cell adhesion, chemokine signaling, tight junctions, and adherent junctions;
➢ Lipotoxic injury resulted in previously unreported complex and multilevel molecular regulation of the hippocampal microvasculature involving transcriptional and post-transcriptional regulation. Post-transcriptional regulation accounted for up to a third of the differential gene expression;
➢ Specific detailed examples of this complex regulation for the representative genes, pathways, transcription factors, and non-coding RNAs are provided below.

### 4.1. Lipotoxic Injury Up-Regulates Hippocampal Microvascular Gene Expression

Our study demonstrated the expected significant differences in cholesterol and lipid levels between the control diet (CD) and the high-fat LR-WD in male mice. The LR-CD mice spontaneously demonstrated hyperlipidemia because of the absence of the LDL receptor. While the physiological implications of severe hyperlipidemia are a topic of further study, it has been shown to correlate with accelerated atherosclerosis and vascular injury in other systems [16]. We decided to carry out our studies in the LR phenotype and recognize that the outcomes may differ in other murine models of hyperlipidemia. We also found in our experimental models serum glucose and insulin changes that were consistent with those previously published [33,34].

However, it was previously unknown how a high-fat diet affects the transcriptome of the brain hippocampal microvessels in males. Using a candidate gene approach in our previous work, we identified activating transcription factor 3 (ATF3) as an important regulator of neuro-inflammation in male mice [10]. The present study significantly extends our prior work and demonstrates for the first time that the WD and LR genotype significantly modulate the differential expression of approximately 5.7% of the hippocampal microvasculature genome of male mice, including protein-coding genes as well as ncRNA (miRNAs, snoRNAs, and LncRNAs), and the analysis suggests a separation between diet and genotype. Analyses of the global expression profile of the genes differentially expressed in at least one of the three comparison study groups showed that the majority of genes were modulated in a similar manner by lipotoxicity, up-regulated. We also identified four clusters of genes containing genes that were down-regulated by the WD in the WT mice compared with the CD, while their expression was up-regulated in the LR mice (Supplemental Figure S7**)**. This observation suggests that lipotoxicity induced by the LR genotype has a potentially greater impact on differential gene expression than the WD and could mask the effect of the WD in the LR genotype.

### 4.2. Regulation of Gene Networks and Pathways for Endothelial Permeability, Neurofunction and Serotonergic Pathways

It has previously been suggested that consumption of fatty acids can modulate gene expression in rat brain [35] and that a high-fat diet can modulate the brain transcriptome in a mouse model of Alzheimer’s disease [12]. These studies suggest that lipotoxic injury can induce changes in different cell types in the brain or in specific brain regions. We have previously shown that exposure of brain microvascular endothelial cells to lipid induces significant modification in the target gene expression profiles [10]. Bioinformatic analyses of protein-coding genes in the present study revealed that lipotoxic injury can also modulate the expression of genes involved in a variety of key cellular processes including gene networks and pathways that regulate endothelial cell adhesion, cytoskeletal organization, neurofunction, cell junctions and chemotaxis, and focal adhesion—all mechanisms involved in the regulation of endothelial permeability [36]. This is of significance because a disruption of endothelial permeability in the brain, where endothelial cells are part of a neurovascular unit, results in BBB dysfunction which may be a significant contributor to the pathogenesis of cognitive impairment, amyotrophic lateral sclerosis, or Alzheimer’s disease [37]. We also found various differentially expressed gene networks that contribute to inflammation, oxidative stress, and Alzheimer’s dementia.

Amongst the differentially expressed genes controlling BBB specifically was GTPase HRas, a GTP-binding protein that plays an important role in cytoskeletal reorganization, cell polarity, cell cycle progression, or angiogenesis [38]. Inadequate angiogenic signaling results in malformed vessels with reduced investiture by pericytes, which results in increased vascular permeability, vascular instability, greater endothelial cell apoptosis and proliferation, and inflammation processes implicated in neurovascular disease [39]. Furthermore, over-expression of HRas results in dilated proliferative blood vessels in the brain, or in blood–brain barrier break-down [39]. Therefore, an increased expression of HRas by lipotoxic injury could lead to an increase in BBB permeability, consistent with our previous work demonstrating increased BBB permeability by fMRI in vivo following a high-fat diet [7].

We also observed that lipotoxic injury differentially regulates the expression of genes involved in pathways related to neurofunction and serotonergic pathways. Among these genes is MOAB, which encodes for Monoamine oxidases isoform B, important in oxidative deamination and thus vascular oxidative stress and endothelial dysfunction [40]. In our study, a lipid injury-induced increased expression of MOAB could therefore lead to increased oxidative stress and brain endothelial dysfunction. The serotonergic synapse pathway was also identified to be differentially expressed and includes genes such as 5-Hydroxytryptamine Receptor 1A (HTR1A) and 5-Hydroxytryptamine Receptor 1B (HTR1B). Interestingly, serotonin/5-hydroxytryptamine (5-HT) and its receptors are known to contribute to atherosclerosis-associated conditions, are associated with chronic inflammation, leukocyte activation, recruitment of leukocytes to endothelial cells [41], and cognition impairment [42]. Taken together, a modulation of the expression of these genes by chronic lipotoxic injury could be associated with cognitive dysfunction and present novel molecular mechanisms of action of lipotoxic injury on brain endothelial cells.

### 4.3. Differential Regulation of Transcription Factors for Endothelial Dysfunction: HNF4a, KLF4, CREB1

Using bioinformatic tools, we identified from the list of differentially expressed genes potential transcription factors (TFs) that could have their activity modulated by lipotoxic injury and be involved in the observed genomic effect. Among the TFs identified, 17 were in common between the study groups. Among them was hepatocyte nuclear factor 4 alpha (HNF4a), whose activity is known to be modulated by fatty acids [43]. HNF4a can in turn modulate both mRNA and the protein expression levels in the brain where it plays a role in depression and physiological homeostasis [44]. Krüppel-like factor 4 (KLF4) is another transcription factor we identified. KLF4 activity is known to be modulated in endothelial cells by a high-fat diet leading to endothelial cell dysfunction, including increased monocyte endothelial adhesion and increased endothelial permeability [45]. This finding suggests another mechanism whereby lipotoxic injury induced by the WD or the LR genotype could induce endothelial dysfunction and increased permeability in the brain. Moreover, several studies indicate that KLF4 is linked to multiple neurological disorders, including Alzheimer’s disease [46]. Other TFs we identified to be differentially expressed by lipid injury, such as CREB1, have also previously been implicated to play a role in vascular dementia [47].

### 4.4. Novel Lipotoxic Injury-Mediated Differential Expression of non-Coding RNAs (miRNA, sno RNA, lncRNA), and their Targets: Implications for Cognitive Dysfunction

In addition to assessing the effect of chronic consumption of the WD on the expression of protein-coding genes, we also identified a previously unknown effect of lipid injury on the expression of ncRNA in the male hippocampal microvasculature. These included microRNAs (miRNAs), small nucleolar RNAs (snoRNAs), and long non-coding RNAs (lncRNAs). This is of significance as ncRNAs have been previously reported to play a vital role in cognition and the vasculature, but the mechanisms have not been defined.

Following a high-fat diet, miRNAs have been reported to be involved in neural function and in metabolic and inflammatory pathways that play a role in atherogenesis [48]. In our study, we observed that lipotoxic injury regulates the expression of a number of microRNAs including up-regulation of mir-678 and mir-210. Mir-678 is known to be regulated by a high-fat diet [13]. Mir-210 has been shown to be up-regulated in the hippocampus and increase cognitive dysfunction in a rat model of vascular dementia (VD) [49]. Mir-210 expression is also important for endothelial cell survival and migration [50]. This suggests that lipotoxic injury-activated mir-210 could impair cognitive function and play a role in endothelial cell migration. Thus, the differential expression of miRNAs mediated by lipotoxic injury could be a mechanism for cognitive dysfunction.

Additionally, some several hundred target genes were identified for each dietary experimental group from the analyses of the predicted miRNA target genes. Interestingly, we observed that 28% of the miRNA target genes overlapped with differentially expressed genes (DEG), suggesting that nearly one-third of the gene expression could be regulated at the post-transcriptional level by lipid injury, while the remaining can be regulated at the level of transcription. The target genes of the differentially expressed miRNAs were involved in pathways related to endothelial cell permeability and function, cell signaling, insulin signaling/resistance, apoptosis, and dementia. Insulin dysregulation has been linked with cognitive dysfunction [51] and endothelial cell dysfunction [52]. It was therefore interesting that among the target DEG miRNAs induced by lipotoxic injury was NGFR, also known as p75NTR (low affinity neurotrophin receptor), which is a target of miRNA-882. p75NTR is known to promote apoptosis of endothelial cells and disrupt angiogenesis in type-1 diabetic mice [53]. In addition, the NGFR levels are low in the hippocampus of learning-impaired aged rats [54]. This suggests that the lipotoxic injury-induced expression of miRNA-882 targets NGFR, which could play a role in apoptosis of endothelial cells and affect cognitive function. Lipotoxic injury also activated miRNA-678 which targets the differentially expressed gene platelet endothelial cell adhesion molecule-1 (PECAM-1). Increased expression of PECAM-1 is found in endothelial cell junctions and plays a role in the BBB [55]. This suggests that miRNA-678 could target the PECAM-1 mRNA expression which could affect endothelial cell adhesion and migration. Taken together, these results are consistent with lipotoxic injury effects on the expression of miRNAs, which post-transcriptionally regulate the expression of mRNA, with functional implications consistent with endothelial cell dysfunction and cognitive dysfunction.

Our microarray analysis also identified the differential expression of other ncRNA other than miRNAs, including snoRNAs and lncRNAs. SnoRNAs play a key role in the regulation of gene expression, post-transcriptional modification of other RNAs, and in stabilizing the genome [56]. We observed that up-regulation in the expression of the brain-specific snoRNA AF357425 (MBII-48) occurred following lipotoxic injury. In the hippocampus, AF357425 is down-regulated following contextual fear memory consolidation [57], a component of memory that is diminished in patients with early-stage AD. Therefore, the observed up-regulation of the AF357425 expression implies that it could contribute to impaired memory consolidation. However, most of the snoRNAs we identified do not currently have known functions, allowing the possibility for the future identification of additional important functional sequelae for the differential expression of snoRNAs following lipid injury.

This study also revealed that lipotoxic injury can control the expression of several lncRNAs. LncRNAs are regulatory RNAs involved in the transcriptional, post-transcriptional, and translational modulation of genes. As such, lncRNAs function in several aging-related processes, including apoptosis, neuronal differentiation, and immune or stress responses [58,59]. We observed an increased expression of the lncRNA A930024E05Rik, also known as LncKdm2b, which positively regulates the transcription of Kdm2b, a histone demethylase that is important for neural development [60] and plays a role in autism and syndromic intellectual disability [61]. Single nucleotide polymorphisms (SNPs) in Kdm2b have been found to increase AD incidence and interact with the APOE e4 gene, a key genetic risk factor in AD [62]. We also observed an increased expression of the lncRNA Ftx, which has been shown to decrease the phosphorylation of vimentin [63], which plays a role in integrin-mediated signal transduction in endothelial cells and is a crucial regulator of focal adhesion organization and endothelial sprouting [64]. This suggests that the inhibition of vimentin by up-regulated Ftx due to lipotoxic injury may lead to defects in endothelial cell adhesion, migration, and signaling. In addition, we observed an increased expression of the lncRNA SNHG7 (small nucleolar RNA host gene 7). GRN is found to be up-regulated in neurodegenerative diseases such as AD and multiple sclerosis and may also function in neuro-inflammation [65,66,67]. Another lncRNA whose expression was increased by lipotoxic injury in male hippocampal microvessels was Gm12603 (WINCR1), important in collective cell migration and collagen contraction [68], suggesting that lipotoxic injury-activated WINCR1 might affect endothelial cell migration and contractibility, and also potentially endothelial permeability.

In summary, to our knowledge, these findings are novel indicators that lipotoxic injury modulates the expression of non-coding RNAs (miRNAs, snoRNAs, and lncRNAs) in the male hippocampal microvasculature via targets that may play an important role in lipotoxic injury-associated brain microvascular disease and vascular dementia.

### 4.5. An Integrated Multi-omics Approach Reveals a Complex and Integrated Transcriptional and Post Transcriptional Response to Lipid Injury in Brain Microvessels

Integrative analyses of the genomic data allowed us to identify the full complexity of the genomic effects of lipotoxicity in the brain microvasculature. Bioinformatic analyses of the genes allowed us to identify the cellular functional pathways in which they were involved, including angiogenesis, apoptosis, and immune cell interactions as well as functional networks, including for cell adhesion and signal transduction. The comparison of the pathways identified separately for the different types of RNAs showed that a third of them were in common for at least two different RNA types and 12 of them were identified for the three different RNA types studied (Figure 7). This observation suggests that cellular processes, including networks of pathways that formed functional groups encompassing cell signaling, oxidative stress, endothelial cell function, and neurologic function, could be affected by lipotoxicity through the regulation of different types of RNAs, providing novel and deeper knowledge regarding the mechanisms of action of lipid injury on brain microvascular molecular regulation. The integrated analysis shown in Figure 7 and Figure 8, revealed for the first time the complex genomic response of the brain microvascular endothelium to lipotoxic injury in vivo in mice. It also demonstrated interactions between different levels of cellular regulators and the need to use systems biology approaches to study lipotoxic injury on cellular function in order to decipher as precisely as possible the underlying molecular mechanisms of action.

Our study is one of a few studies that have analyzed multi-omic regulation simultaneously and integrated different types of RNAs in the brain microvasculature [69,70], and to our knowledge, ours is the only study that has used a multi-omics approach to show the role of various RNA types in the brain microvasculature following lipid injury in male mice. As presented in the example of the regulation of the cytoskeleton pathways, we identified differentially expressed protein-coding genes and also target genes of miRNAs and lncRNAs (Figure 8A,B). Interestingly, important interactions were observed as some of the targets of differentially expressed miRNAs were also identified as differentially expressed, and could also be targets of lncRNAs. Therefore, by regulating the expression of lncRNAs, lipid injury can impact miRNAs and consequently their target genes, but also the differential expression of protein-coding genes. In this manner, our integrated analysis revealed a large interconnecting cascade between the three levels of genomic regulation of cell function (protein-coding genes, possible transcription factors, and potential post-transcriptional regulation via non-protein-coding mechanisms involving primarily miRNAs and lncRNAs) (Supplemental Figure S8), especially for the WD-fed LR mice, our experimental model with the highest degree of lipid stress. The novel, complex, and substantial multilevel genomic effect of lipotoxic injury on the brain microvasculature could help explain the deleterious impact of the Western diet on brain microvascular function and cognitive performance.

### 4.6. Summary and Implications for Future Research

In alignment with our hypothesis, our results showed profound transcriptome changes in response to the WD in the hippocampal microvasculature of male mice including the modulation of protein-coding genes, miRNAs, snoRNAs, and lncRNAs, as well as the corresponding cellular functional pathways, and the mechanism of regulation by transcription factors. Integrative analyses of the genomic data also identified the cellular functions primarily affected by lipid-associated vascular injury to include angiogenesis, apoptosis, immune cell interactions, and gene regulation and RNA biogenesis, which could be related to vascular injury or response to lipid injury. Among these gene networks and pathways, endothelial cell adhesion, cytoskeleton organization, neurofunction, cell junctions and chemotaxis, and focal adhesion are regulated—mechanisms involved in the regulation of endothelial permeability. This is of significance because a disruption to the endothelial permeability in the brain, where endothelial cells are part of a neurovascular unit, results in BBB dysfunction, which may be a significant contributor to the pathogenesis of cognitive impairment, Alzheimer’s disease, and other related dementias. As such, our work helps to significantly advance the field and elucidate the molecular mechanisms whereby the Western diet disrupts brain microvascular endothelial function and how that may predispose to vascular dementia. Future work will need to address whether and how the high-glycemic component of a Western diet contributes to microvascular lipid injury in the brain, the reversibility of diet associated vascular dementia, and the interaction of genetic factors and diet on vascular dementia.

## Figures and Tables

**Table 1:** Effect of the Western diet on the expression of small nucleolar RNAs (snoRNAs) and long non-coding RNAs (lncRNAs) in male hippocampal microvessels.

SnoRNAs	WTWD vs. WTCD	LDL-R−/− CD vs. WTCD	LDL-R−/− CD vs. WTCD	lncRNAs	WTWD vs. WTCD	LDL-R−/− CD vs. WTCD	LDL-R−/− CD vs. WTCD
Snord61	−39.34			AI504432		56.21	
Gm25443	−2.32			Cep83os		49.06	
AF357355		123.27		Zfp91Cntf		19.36	27.1
Gm25635		90.8		D4Ertd617e		12.6	
Gm25856		55.53		2810049E08Rik		11.51	
Gm22289		45.23		Mir124-1		10.4	
Snord95		27.98		Ftx *		6.43	
Gm25607		12.59		F420014N23Rik		5.55	6.86
Snora17		11.93	64.98	1700110C19Rik		4.07	
Gm23123		9.93	6.21	AI314278		2.98	
Snora23 *		8.96		2310069G16Rik		2.4	
Snord88c		7.89		1700027J07Rik		2.27	2.5
Gm24013		6.77		1700009J07Rik		2.23	
Gm24336		6.38	33.24	1700024B18Rik		2.23	
Gm25125		6.24		D130017N08Rik		2.08	
Gm24770		5.9	6.8	C130071C03Rik *		2.87	
Gm23456		5.32	11.45	Gm10010		4.65	
Gm24844		5.3		Gm13411		3.58	3.32
Gm25092		3.89		Gm15409		2.92	
Snord107		3.77		Gm26593		2.84	
Snord16a *		3.73		Gm26643		2.68	
Gm22546		3.59		Gm14254		2.06	
Gm24284		3.07		AU022754		2.22	
Snord72		2.99		1700016L21Rik		2.2	
Gm26070		2.72		Snhg7os		2.1	
Gm23722		2.4		1700027H10Rik		2.08	
Snora5c		2.38		Pcsk2os2		2.11	2.03
Gm25410		2.24		Plet1os		2.15	
Gm22531		2.21		Gldnos		2.15	
Gm24429		2.09		Sp3os		2.31	
Gm26272		2.09		4921534H16Rik		2.24	
Gm25860		2.05		4930405O22Rik		2.21	
Gm26387		2.04		5330413P13Rik		2.19	2.3
Snord61		−27.1	−29.61	6330415B21Rik		5.62	
Gm23199		32.34		Mhrt *		3.72	
Gm24400		20.1		Med9os		3.15	
Gm23121		17.62		9530082P21Rik		2.75	
Gm22935		11.9		A930024E05Rik *		2.52	
Gm24878		9.74		9430083A17Rik		2.45	
Gm25401		9.05		Gm26583		2.22	
Gm25720		6.28	67.3	Gm14061		2.13	
Gm22378		6.25		Gm26777		15.18	
Gm24449		5.46		Gm15323		3.28	
Gm22485		3.66		Gm15322		3.28	
Gm25371		3.12		Gm12121		2.7	
Gm24504		2.57		Gm22		2.03	
Gm25982		2.14		Atcayos			5.91
Gm24678		2.03	3.88	Gm10790			5.16
Gm24682		3.65		Chn1os3			3.42
Snhg7		11.93		5730420D15Rik			3.27
Snord66 *			70.95	Gm16793			3.2
Gm23546			66.56	Gm10390			3.07
Gm25777			36.23	Arhgap33os			3
Gm25788			29.72	B230312C02Rik			2.98
Gm23734			18.87	4632428C04Rik			2.96
Gm24916			11.99	4933424G05Rik			2.65
Gm22144			11.18	Gm19784			2.59
Gm24771			11.03	Gm12603 *			2.59
Gm22504			8.42	D5Ertd605e			2.54
Snord14a *			7.54	9330102E08Rik			2.51
Gm23527			5.66	9230105E05Rik			2.43
Gm26148			4.58	9630013K17Rik			2.43
Gm25376			4.27	4933432I03Rik			2.36
Gm25432			3.18	4930565D16Rik			2.35
Gm25715			3.1	Gm16548			2.33
Gm23534			2.94	Gm13985			2.31
Gm26047			2.88	Gm10007			2.24
Gm25526			2.81	Gm10619			2.23
Rnu3a			2.76	4930568E12Rik			2.2
Snord53 *			2.72	C130080G10Rik *			2.19
Gm24127			2.67	4930522O17Rik			2.19
Gm24581			2.26	Gm13003			2.14
Gm24648			2.23	4930444M15Rik			2.12
Gm25945			2.17	4931403G20Rik			2.11
Gm23136			2.16	4930488L21Rik			2.11
Gm23129			2.13	1700113A16Rik			2.1
Gm24252			2.13	Gm5144			2.09
Gm22840			2.11	Lincred1 *			2.09
Gm24313			2.07	4933433G08Rik			2.08
Gm22269			2.05	A930019D19Rik			2.07
AF357425 *			−22.59	1700047A11Rik			2.07
ScaRNA15			8.54	2500002B13Rik			2.07
Gm24668			167.27	4930455F16Rik			2.05
Gm22940			75.5	Hoxaas3 *			2.05
Gm23970			14.37	1700045H11Rik			2.04
Gm23000			4.21	Gm15413			2.04
Gm22883			2.56	1700064J06Rik			2.02
Gm23031			2.21	5430434I15Rik			2.02
Gm24524			2.03	1700066N21Rik			2.01
Gm23119			63.09	C530044C16Rik			2.01
				Snhg7 *			64.98
				Gm12590			28.43
				Gm26906			4.55
				Gm26675			4.4
				Gm6410			3.92
				Gm9898			2.58
				Gm10425			2.45
				Gm13790			2.42
				Gm28890			2.36
				Gm26542			2.26
				Gm16295			2.19
				Gm14684			2.17
				Gm26656			2.1
				Gm15556			2.07
				Gm12148			2.05
				Gm12637			2.05

* denotes snoRNAs and LncRNAs of a known function.

## Supplementary Materials

The following are available online at https://www.mdpi.com/2072-6643/12/6/1771/s1. Figure S1: Representative images of hippocampal neurons and microvessels dissected by laser capture microdissection. Figure S2: Mean body weight of wild type (WT) and LDL-R −/− mice pre- and post-feeding with the control (CD) and Western (WD) diets. Figure S3: Volcano plot of gene expression changes in hippocampal microvessels. A) WT-WD vs. WT-CD, B) LDL-R −/− CD vs. WT-CD and C) LDL-R −/− WD vs. WT-CD. Figure S4: Gene ontology biological processes tree map of differentially expressed protein coding genes in hippocampal microvessels. Figure S5: (A) Venn diagrams representing the number of differentially expressed (DE) genes, compared to miRNA target genes, affected by diet and genotype in hippocampal microvessels. (B) Venn diagrams representing the pathways of differentially expressed (DE) genes, compared to pathways of miRNA target genes, affected by diet and genotype in hippocampal microvessels. Figure S6: Regulation of actin cytoskeleton pathway. (A) Differentially expressed genes. (B) Differentially expressed miRNAs and their targets. (C) Differentially expressed LncRNAs and their targets. Figure S7: Effect of the Western diet and LDL-R −/− genotype on differentially expressed protein-coding genes in hippocampal microvessels. Figure S8: Schematic presentation of genomic modifications and interactions in brain hippocampal microvascular following Western diet consumption of LDR knock-out. Table S1: Primer sequences for genes tested by qRT-PCR were prepared by the Primer3 software using Affymetrix transcript ID sequences. Table S2: (A) Plasma lipid levels of wildtype (WT) and LDL-R−/− mice fed with control (CD) and Western (WD) diet. B) Plasma glucose and insulin levels of wildtype (WT) and LDL-R−/− mice fed with control (CD) and Western (WD) diet. Table S3: Differentially expressed genes in Western diet (WD)-fed WT mice when compared to control diet (CD)-fed WT mice. Table S4: Differentially expressed genes in control diet (CD)-fed LDL-R−/− mice when compared to control diet (CD)-fed WT mice. Table S5: Differentially expressed genes in Western diet (WD)-fed LDL-R−/− mice when compared to control diet (CD)-fed WT mice. Table S6: Transcription factors affected by Western diet and LDL-R−/− in hippocampal microvascular endothelium. Table S7: Effect of the Western diet on the expression of microRNAs (miRNAs) in male hippocampal microvessels.
