# Supplementary material for: Lipotoxic Injury Differentially Regulates Brain Microvascular Gene Expression in Male Mice
Source: Nutrients. 2020 Jun 13;12(6):1771. doi: 10.3390/nu12061771 (PMC7353447; doi:10.3390/nu12061771)
Supplement: Supplementary file 1 [file nutrients-12-01771-s001.pdf]

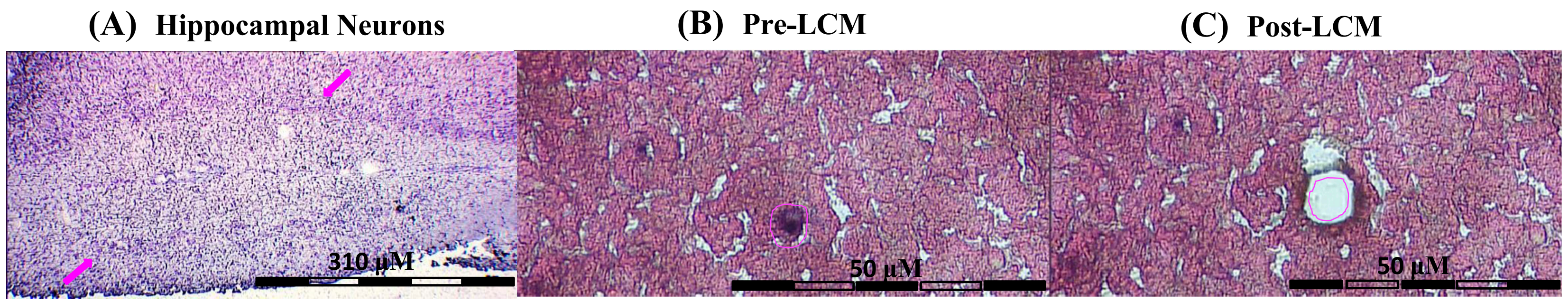

Supplement Figure S1: Representative images of hippocampal neurons and microvessels dissected by laser capture microdissection. Neurons in the hippocampus of Western diet (WD)-fed and control diet (CD)-fed low density lipoprotein receptor (LDL-R) -/- and C57BL/6J (WT) male mice were identified by hematoxylin staining (panel A, pink arrows). Microvessels in the hippocampus were identified by alkaline phosphatase staining and subjected to laser capture microdissection (LCM). The middle panel (panel B) shows the outline of an entire microvessel pre-LCM, and the right panel (panel C) shows the same microvessel post-LCM. Scale bar = 310 um panel A, and 50 μm panels B and C.

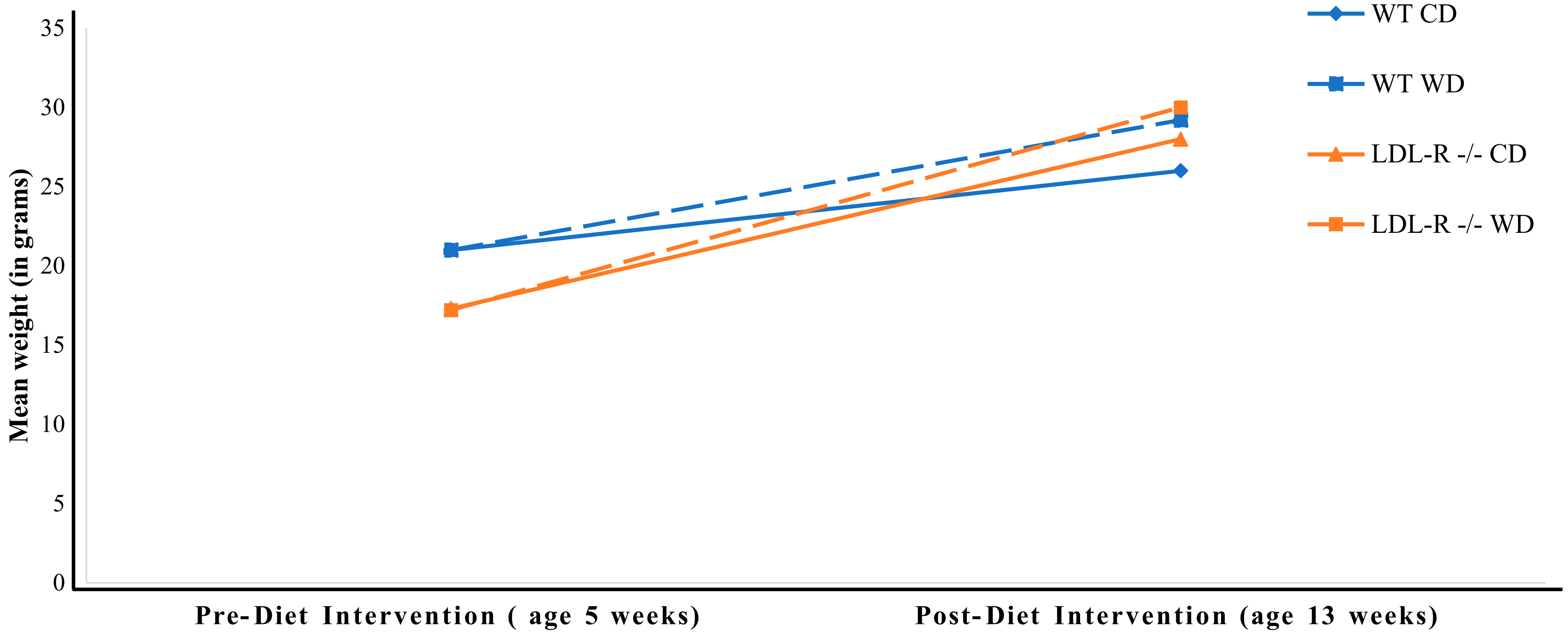

Supplement Figure S2: Mean body weight of wild type (WT) and LDL-R -/- mice pre- and post-feeding with the control (CD) and western (WD) diets. Line graph shows mean weight (grams) of wild type (WT) and LDL-R -/- mice before feeding (at age 5 weeks, solid lines) and after feeding (at age 13 weeks, dashed lines) with the control (CD) and western (WD) diets. Weight increased for mice in all the 4 groups (WT CD, WT WD, LDL-R -/- CD and LDL-R -/- WD) post the diet when compared to pre-diet intervention ($p<0.05$ for all pre/post comparisons).

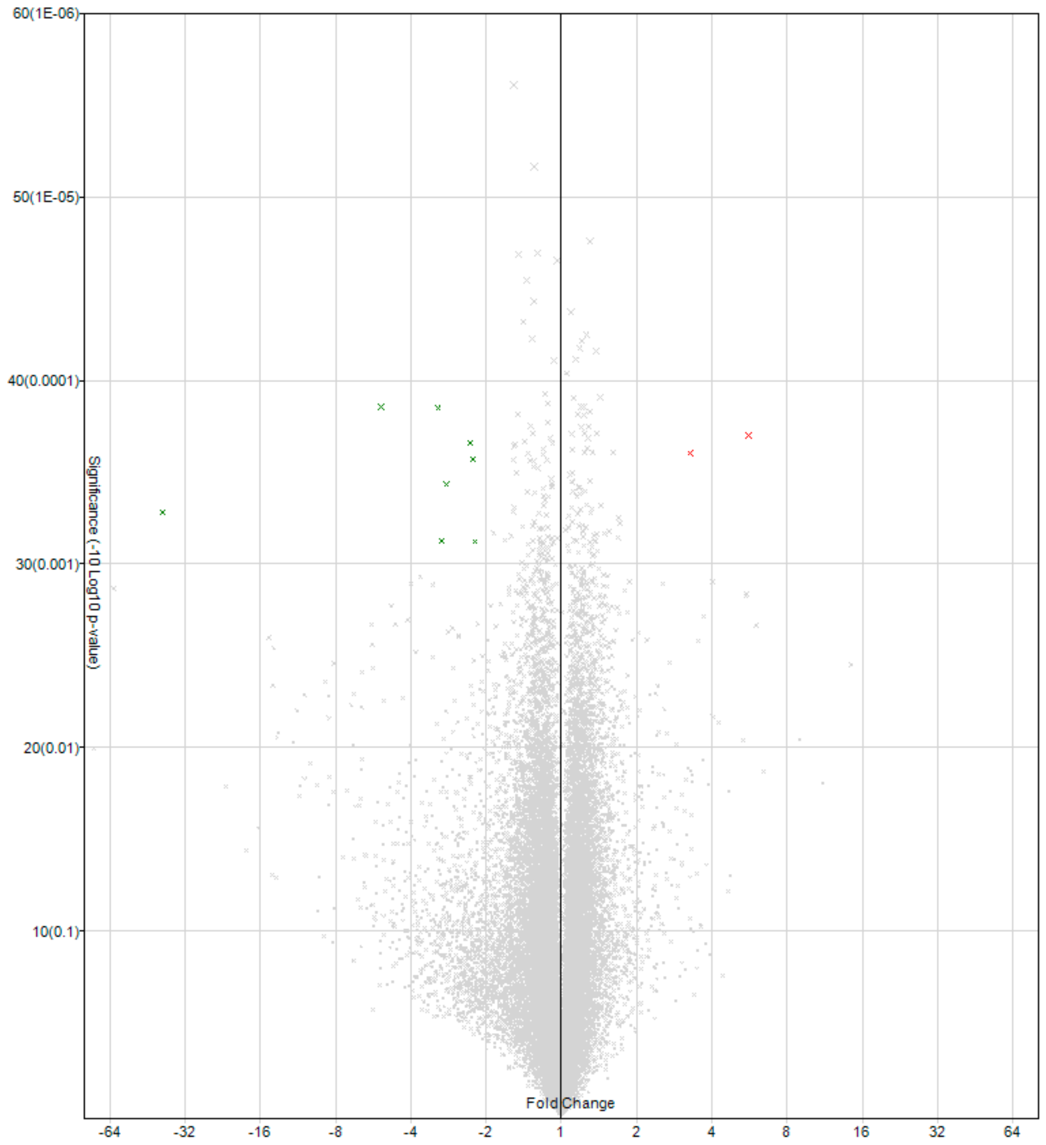

Supplement Figure S3A: Volcano plot of gene expression changes in hippocampal microvessels in WT WD vs WT CD. The transcriptome of microvessels from western diet (WD) fed C57BL/6J (WT) compared to microvessels from control diet (CD) fed WT mice. The x-axis specifies the fold-changes (FC) and the y-axis specifies the negative logarithm to the base 10 of the p-values. Red vertical and horizontal lines reflect the filtering criteria (FC = fold change, ±2.0 and p-value = 0.05). Red and green dots represent probe sets for transcripts expressed at significantly higher or lower levels than control mice (CD fed WT mice), respectively.

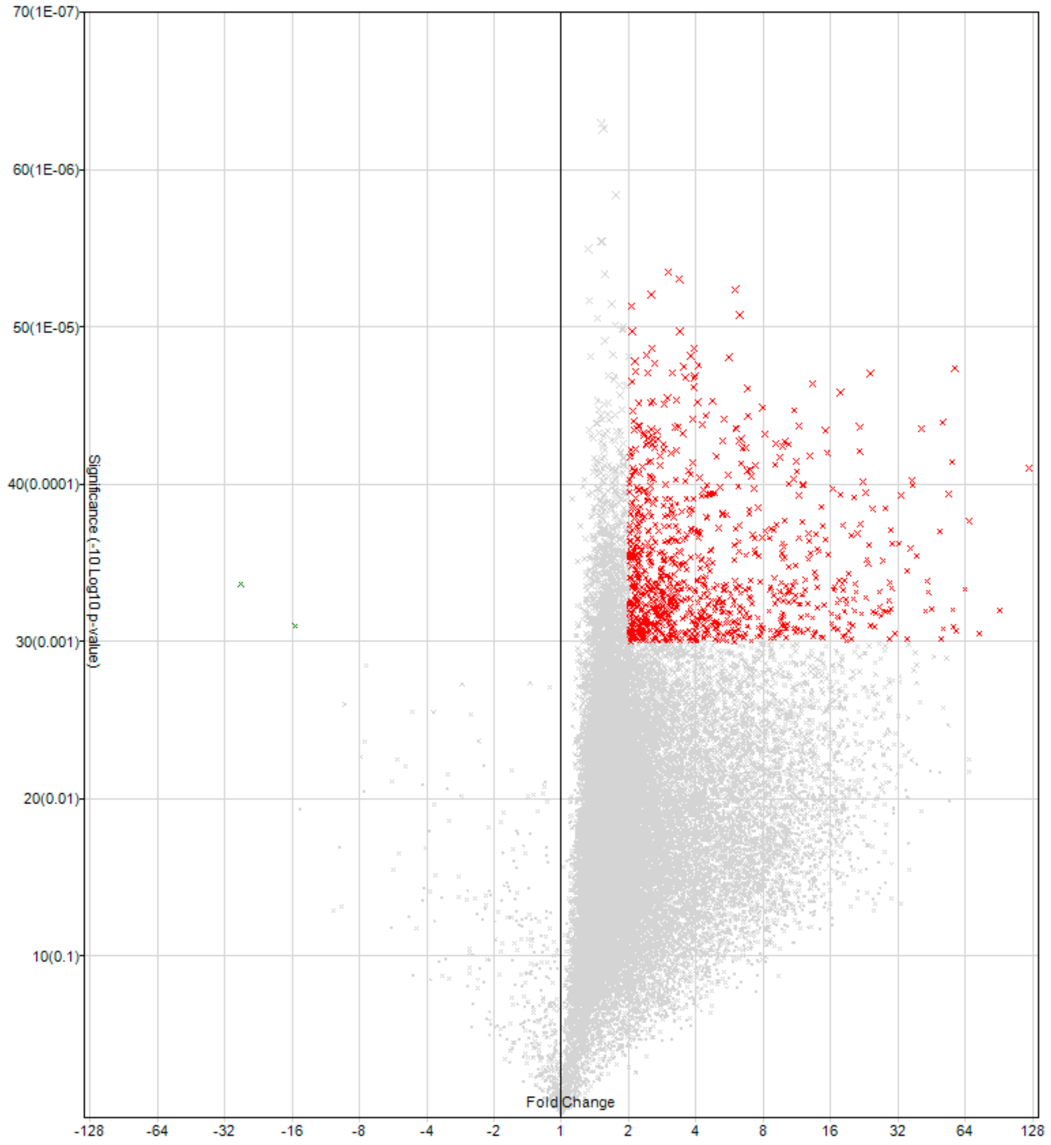

LDL-R -/- CD vs WT CD

Supplement Figure S3B: Volcano plot of gene expression changes in hippocampal microvessels in LDL-R -/- CD vs WT CD. The transcriptome of microvessels from CD fed LDL-R -/- mice compared to microvessels from CD fed WT mice. The x-axis specifies the fold-changes (FC) and the y-axis specifies the negative logarithm to the base 10 of the p-values. Red vertical and horizontal lines reflect the filtering criteria (FC = fold change, ±2.0 and p-value = 0.05). Red and green dots represent probe sets for transcripts expressed at significantly higher or lower levels than control mice (CD fed WT mice), respectively.

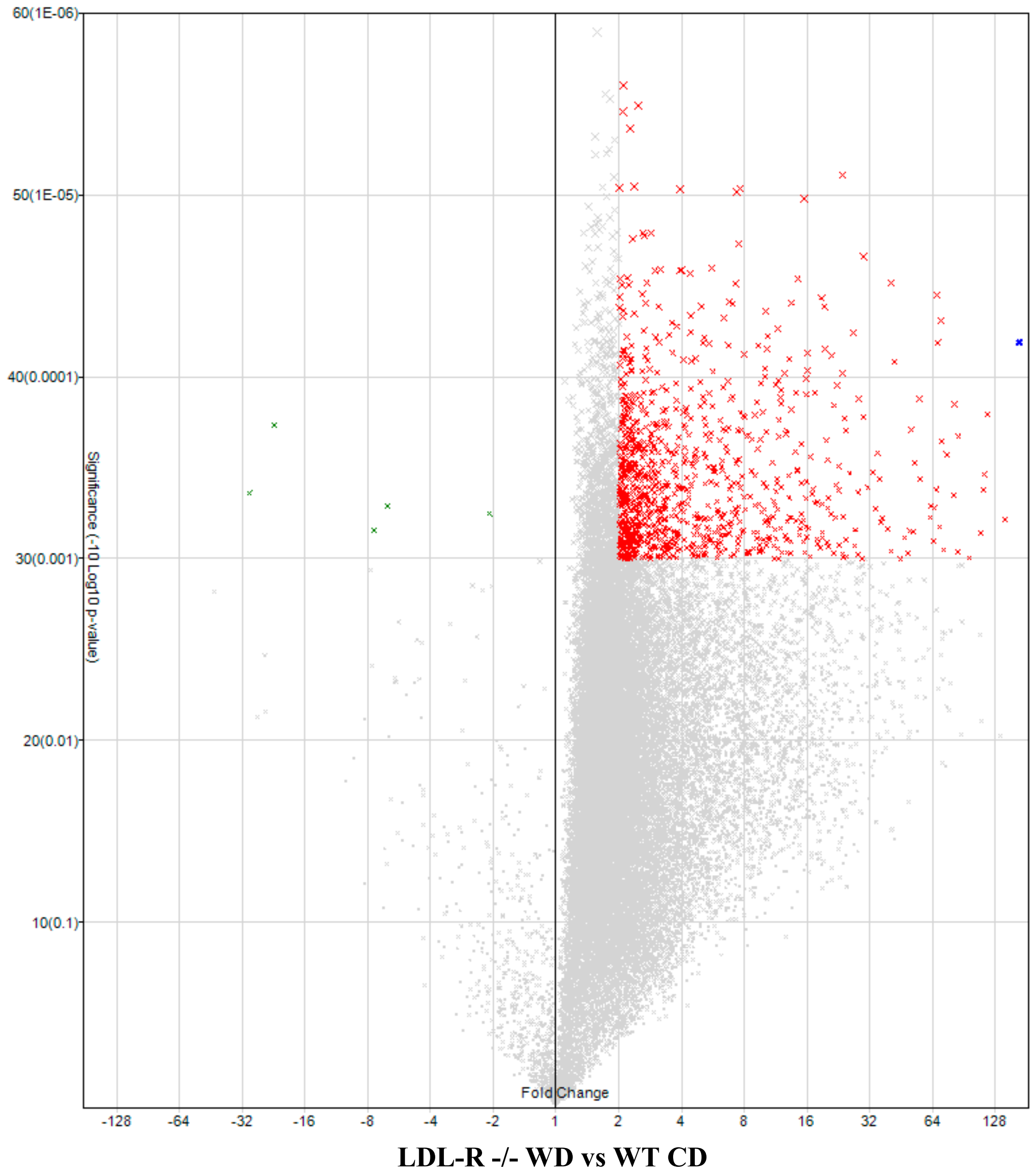

Supplement Figure S3C: Volcano plot of gene expression changes in hippocampal microvessels in LDL-R -/- WD vs WT CD. The transcriptome of microvessels from WD fed LDL-R -/- mice compared to microvessels from CD fed WT mice. The x-axis specifies the fold-changes (FC) and the y-axis specifies the negative logarithm to the base 10 of the p-values. Red vertical and horizontal lines reflect the filtering criteria (FC = fold change, ±2.0 and p-value = 0.05). Red and green dots represent probe sets for transcripts expressed at significantly higher or lower levels than control mice (CD fed WT mice), respectively.

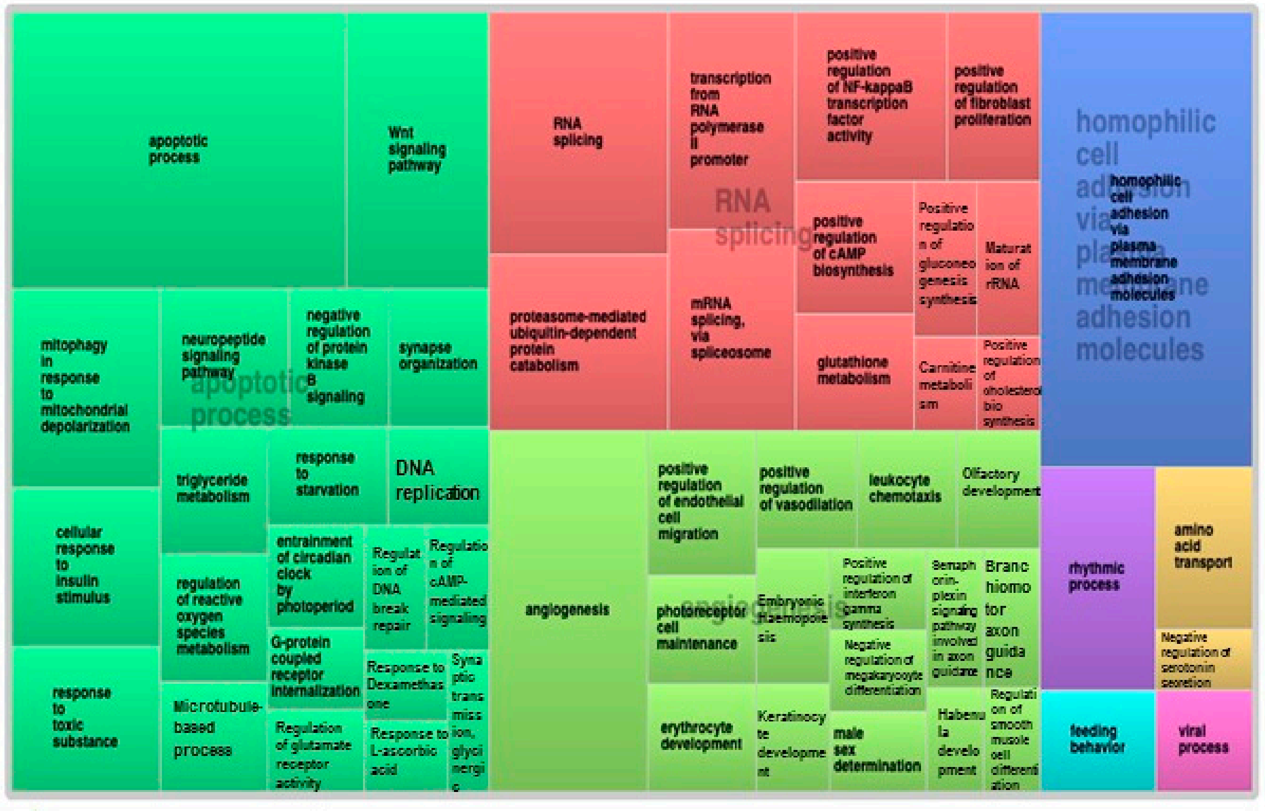

Supplement Figure S4: Gene ontology biological processes tree map of differentially expressed protein coding genes in hippocampal microvessels. Gene ontology (GO) biological processes of differentially expressed genes (DEG) in hippocampus microvessels from Western diet (WD) fed C57BL/6J (WT) mice compared to control diet (CD) fed WT mice, CD fed LDL-R -/- mice compared to CD fed WT mice, and WD fed LDL-R -/- mice compared to CD fed WT mice was identified using David bioinformatics database and tree map was constructed using Revigo. DEG are involved in various biological functions such as angiogenesis (light green rectangle boxes), apoptotic processes (dark green rectangle boxes), RNA splicing (red rectangle boxes) and cell adhesion (blue rectangle box). Sizes of the rectangle boxes in the tree map are adjusted based on the frequency of the GO biological process.

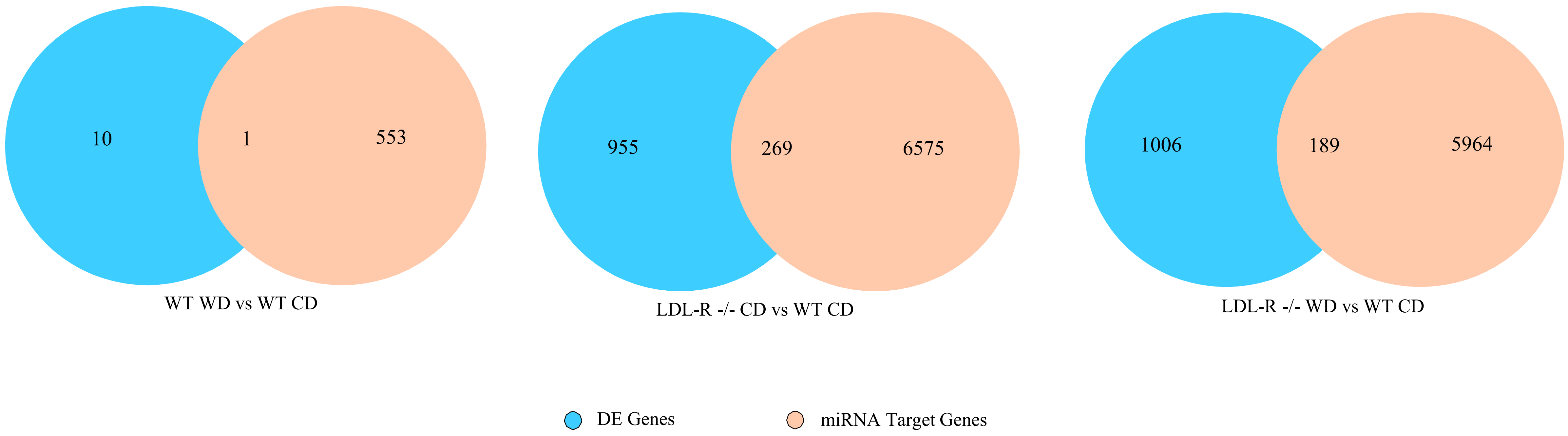

Supplement Figure S5A: Venn diagrams representing the number of differentially expressed (DE) genes, compared to miRNA target genes, affected by diet and genotype in hippocampal microvessels. Venn diagrams of differentially expressed (DE) genes and miRNA target genes showing 1, 269, and 189 genes in common for WD fed WT mice, CD fed LDL-R -/- mice, and WD fed LDL-R -/- mice, respectively, when compared to CD fed WT mice. Genes targeted by miRNAs were identified using the miRWalk database.

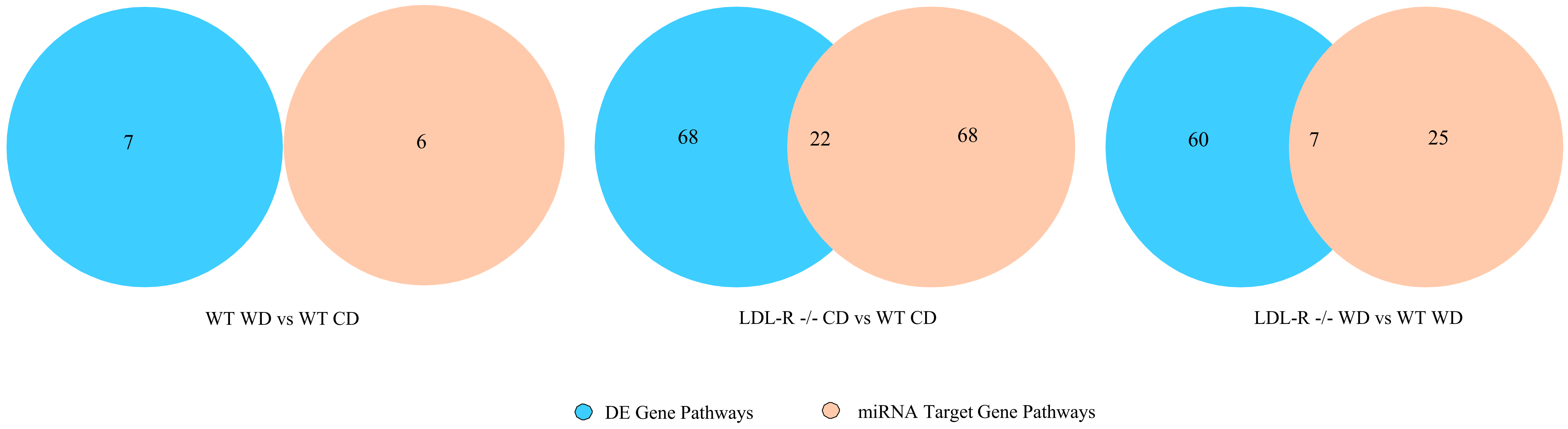

Supplement Figure S5B: Venn diagrams representing the pathways of differentially expressed (DE) genes, compared to pathways of miRNA target genes, affected by diet and genotype in hippocampal microvessels. Venn diagrams of differentially expressed (DE) gene pathways and miRNA target gene pathways showing 0, 22, and 7 pathways in common for WD fed WT mice, CD fed LDL-R -/- mice, and WD fed LDL-R -/- mice, respectively, when compared to CD fed WT mice. Pathways of DE genes and miRNA target genes were identified using the KEGG database and Genetrail2.

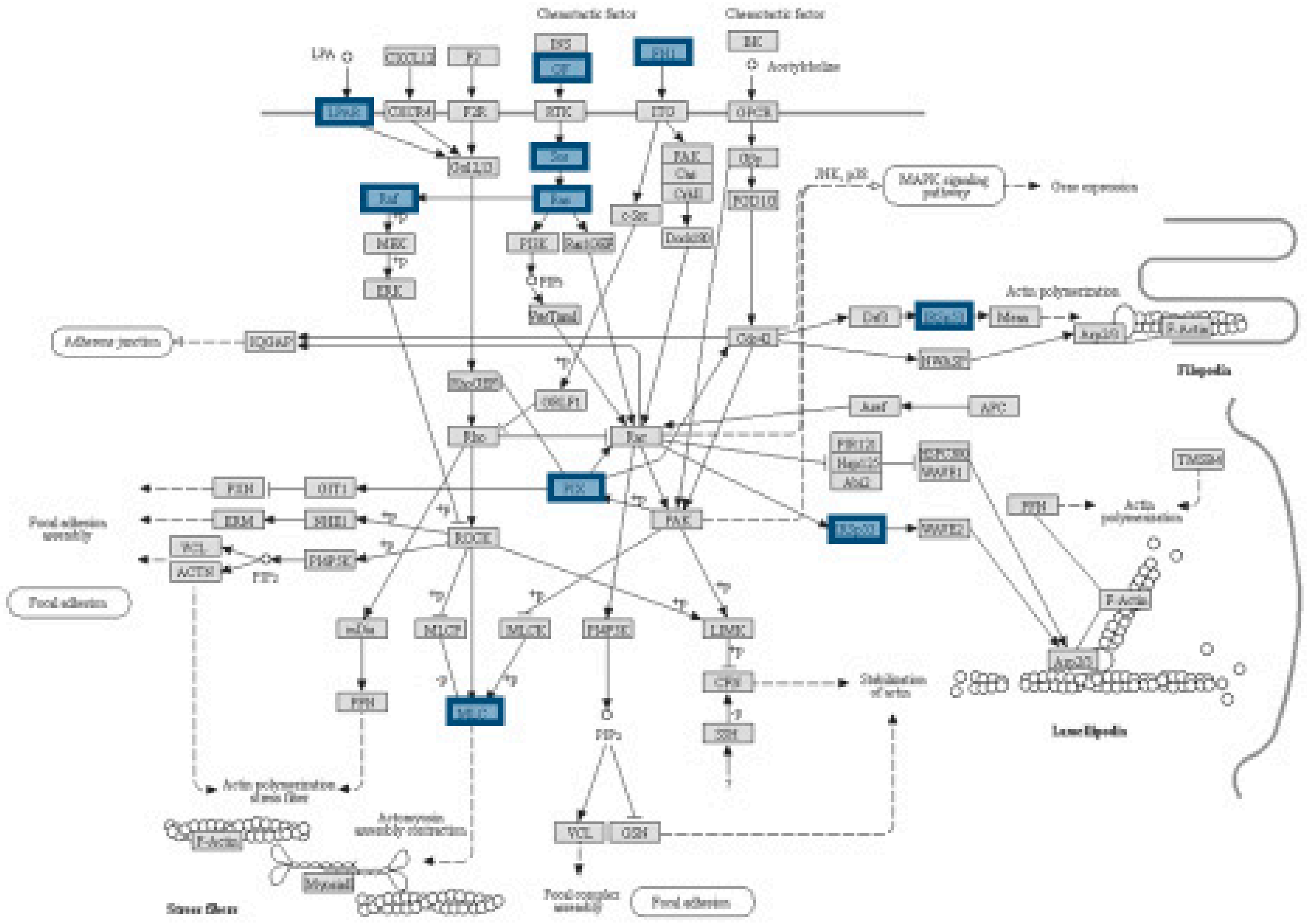

Supplement Figure S6A: Differentially expressed genes in regulation of the actin cytoskeleton pathway. Differentially expressed protein coding genes are shown in the blue boxes.

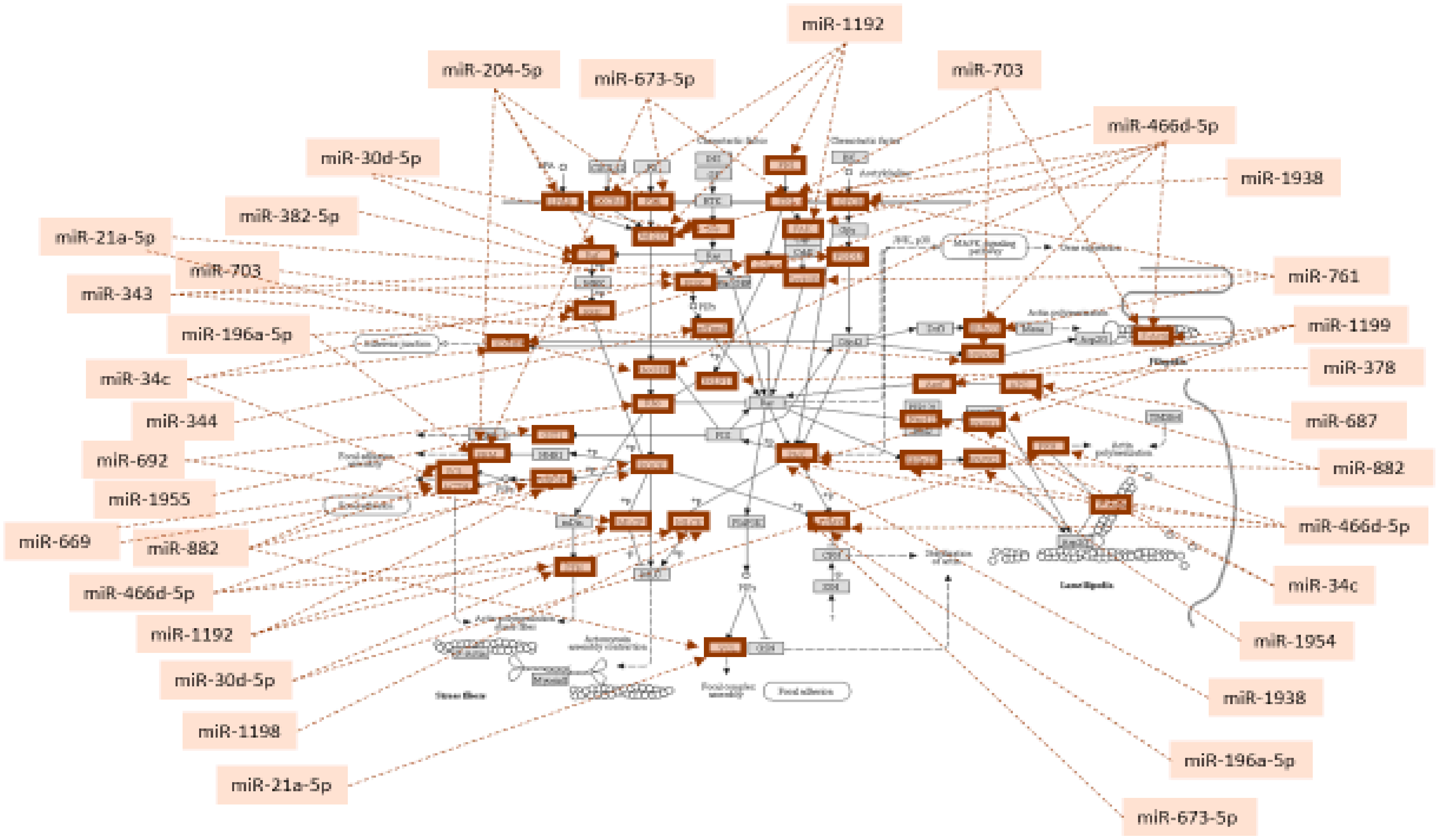

Supplement Figure S6B: Differentially expressed miRNAs and their targets in regulation of the actin cytoskeleton pathway. Differentially expressed miRNAs are shown in the brown boxes and their target by the brown broken arrows.

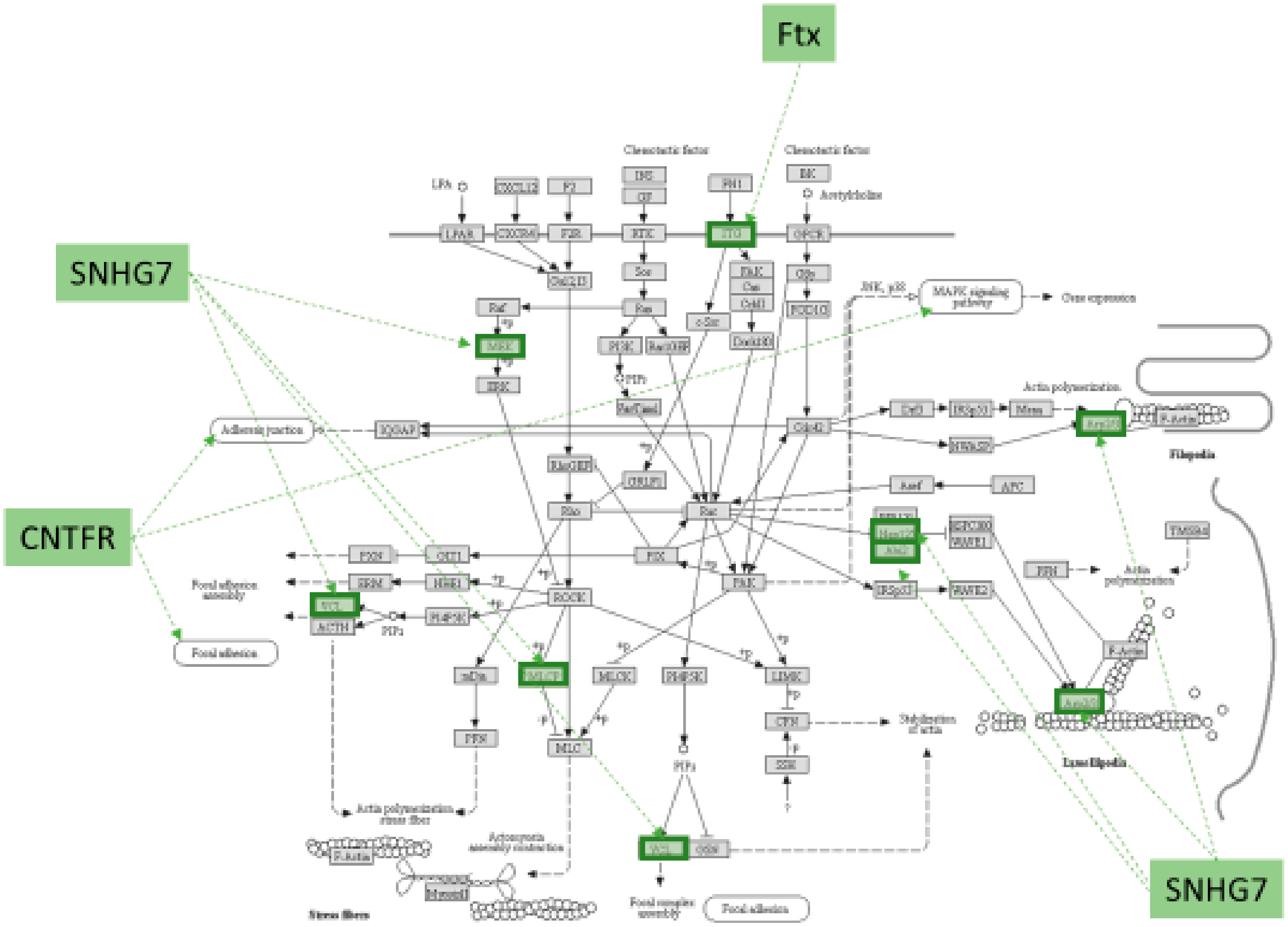

Supplement Figure S6C: Differentially expressed long non coding RNAs (lncRNAs) and their targets in regulation of the actin cytoskeleton pathway. Differentially expressed lncRNAs are shown in the green boxes and their targets by the green broken arrows.

Supplement Figure S7

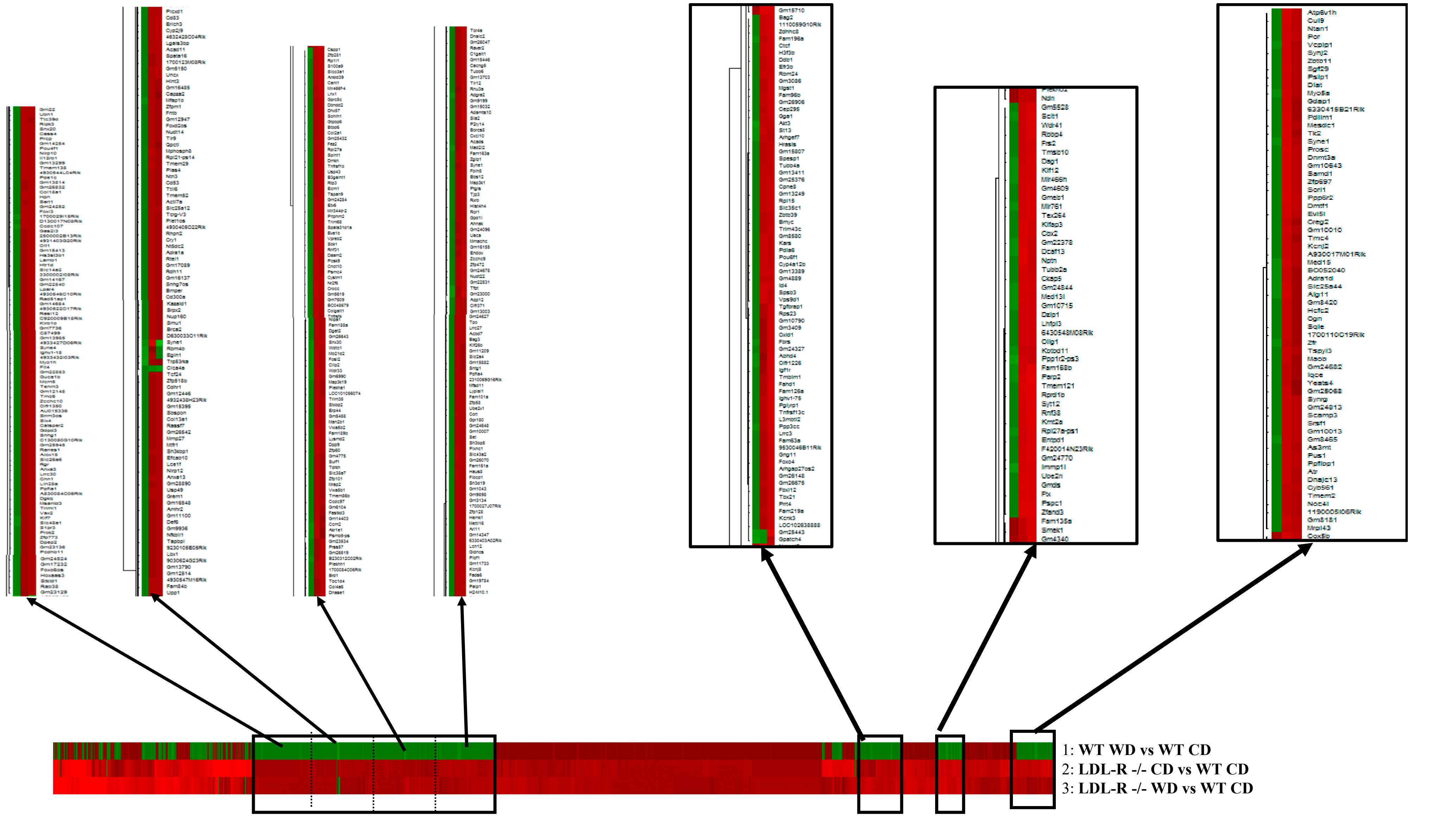

Supplement Figure S7: Effect of the western diet and LDL-R - /- genotype on differentially expressed protein-coding genes in hippocampal microvessels. Heat map showing the up-regulation (red) and down-regulation (green) of differentially expressed genes (DEG) compared to control C57BL/6J (WT) mice fed a control diet (CD). Individual DEG are represented in rows, and the three different experimental comparison groups in columns, as follows: column 1: WT mice on the western diet (WD) compared to WT mice on control diet (CD); column 2: LDL-R -/- mice on CD compared to WT mice on CD; column 3: LDL-R -/- mice on WD compared to WT mice on CD. Black rectangle boxes indicates DEG which are down regulated in the diet group (WT mice on WD compared to WT mice on CD) and up- regulated in the genotype groups (LDL-R -/- mice on CD and LDL-R -/- mice on WD compared to WT mice on CD.

WD WT CD

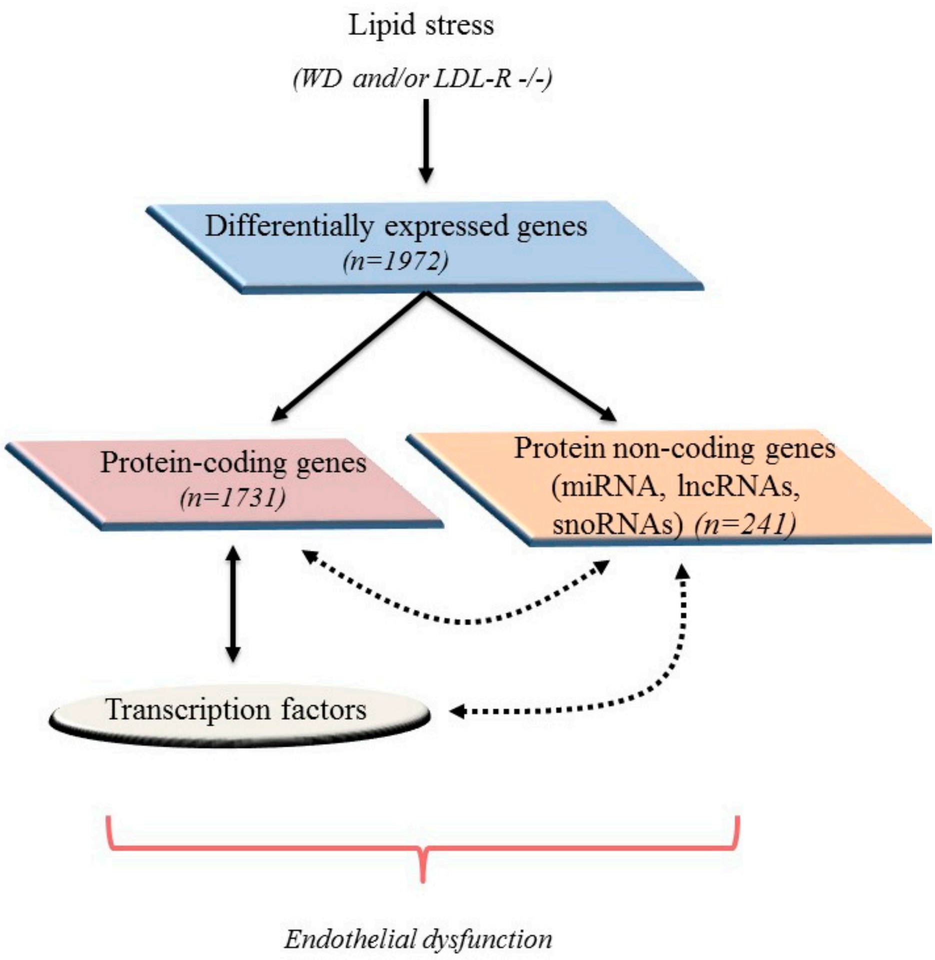

Supplement Figure S8: Schematic presentation of genomic modifications and interactions in brain hippocampal microvascular following western diet consumption of LDR knock-out.

Table S1: Primer sequences for genes tested by qRT-PCR were prepared by Primer3 software using Affymetrix transcript ID sequences.

Gene	Primer Sequence (5′-3′)
GAPDH	Sense- GCAACAGGGTGGTGGACCT
	Antisense- GGATAGGGCCTCTCTTGCTCA
Npy	Sense- CCGGTGGATCTCTTCTCTCA
	Antisense- CCCCATTCGCTTGTTACCTA
Taf1d	Sense- GCATGGTATCTGCACTCAGC
	Antisense- ATGCACAAAGCCAAGAAACC
Trp53rka	Sense- CCTACGTGGGTGTCTGGAGT
	Antisense- ACCACACTGGAGTCCTTTGG
Egln1	Sense- TACAGGATAAACGGCCGAAC
	Antisense- CGCATCTTCCATCTCCATTT
Arrb1	Sense- TCCTGGCACAGAGACACTTG
	Antisense- AATTCATTCCCAAGGTGCAG
Aph1a	Sense- TATGGCCTCCTGATTTTTGG
	Antisense- GATGCTAAGCCCTCATCTGC
Fabp5	Sense- TGCAACAAACAGCTTCACTTC
	Antisense- TCCTGGGTAAACCAAGTTTGA
Slc17a5	Sense- CCCCTGTTACAGCCACTGTT
	Antisense- TTTCCCTCGTGCTTGTCTCT
Rap1b	Sense- GCTCTGAGCCAGGTCTGAAG
	Antisense- CACCACAGGAAAGTCCGTTT
Clca4a	Sense- CATCCACTTCACCCCTGACT
	Antisense- AAATACTCCCCAGCGAAGGT
MAPK8	Sense- TTTTGCTGTGAAACTTTTGATTATCA
	Antisense- AACTTAACATGTGGTGCAATTTCTGT

Table S2A: Plasma lipid levels of wildtype (WT) and LDL-R -/- mice fed with control (CD) and western (WD) diet.

Plasma Lipids (Mean±SEM)	WT-CD	WT-WD	LDL-R -/- CD	LDL-R -/- WD
**TC (mg/dL)**	89.3±1.6	252.8±21.5*	285.6±41.1*	1151.8±38.1*
**TG (mg/dL)**	28.3±5.7	35.6±7.0	171.5±38.6*	262.2±59.7*
**LDL (mg/dl)**	6.1±0.8	44.6±7.2*	134.9±39.1*	983.1±17.0*
**HDL (mg/dL)**	77.6±1.5	201.0±16.7*	116.4±15.8	116.2±16.5

** p <0.05 for TC, TG, LDL and HDL compared to CD fed WT mice*

Table S2B. Plasma Glucose and Insulin levels of wildtype (WT) and LDL-R -/- mice fed with control (CD) and Western (WD) diet.

Mean±SEM	WT-CD	WT-WD	LDL-R -/- CD	LDL-R -/- WD
**Glucose (mg/dL)**	292.1±0.98	494.3±2.72*	345.1±14.06*	360.0±21.47*
**Insulin (pg/mL)**	232.0±9.89	918.1±57.69*	349.4±33.62*	462.7±80.44*

** p <0.05 for glucose and insulin compared to CD fed WT mice*

Table S3: Differentially expressed genes in Western diet (WD) fed WT mice when compared to control diet (CD) fed WT mice.

Transcript Cluster ID	Gene Symbol	Description	Fold Change
17458514	Npy	neuropeptide Y	5.61
17369948	Mir1954	microRNA 1954	3.28
17514135	Egln1	egl-9 family hypoxia-inducible factor 1	-2.22
17356279	Rbm4b	RNA binding motif protein 4B	-2.25
17357717	Gm25443	predicted gene, 25443 [Source:MGI Symbol;Acc:MGI:5455220]; (scaRNA from ensembl)	-2.32
17400549	Gm20634	predicted gene 20634 [Source:MGI Symbol;Acc:MGI:5313081]; (processed transcript from ensembl)	-2.89
17398915	Gpatch4	G patch domain containing 4	-3.03
17394478	Trp53rka	transformation related protein 53 regulating kinase A	-3.12
17514828	Taf1d	TATA box binding protein (Tbp)-associated factor, RNA polymerase I, D	-5.27
17541917	Snord61	small nucleolar RNA, C/D box 61	-39.34

Table S4: Differentially expressed genes in control diet (CD) fed LDL-R -/- mice when compared to control diet (CD) fed WT mice.

Transcript Cluster ID	Gene Symbol	Description	Fold Change
17278775	AF357355	snoRNA AF357355	123.27
17239182	Gm25635	predicted gene, 25635 [Source:MGI Symbol;Acc:MGI:5455412]	90.8
17238882	Syne1	spectrin repeat containing, nuclear envelope 1	73.78
17238832	Syne1	spectrin repeat containing, nuclear envelope 1	66.53
17446058	Gm24009	predicted gene, 24009 [Source:MGI Symbol;Acc:MGI:5453786]	63.73
17495541	Syt17	synaptotagmin XVII	58.05
17547614			57.35
17401530	AI504432	expressed sequence AI504432	56.21
17531181			56.12
17278793	Gm25856	predicted gene, 25856 [Source:MGI Symbol;Acc:MGI:5455633]	55.53
17238884	Syne1	spectrin repeat containing, nuclear envelope 1	53.87
17228860	Aph1a	anterior pharynx defective 1a homolog (C. elegans)	50.78
17245700	Tspan31	tetraspanin 31	50.59
17548717	Fabp5	fatty acid binding protein 5, epidermal	49.88
17244435	Cep83os	centrosomal protein 83, opposite strand	49.06
17491632	Gm22289	predicted gene, 22289 [Source:MGI Symbol;Acc:MGI:5452066]	45.23
17262193	Cdk2ap1	CDK2 (cyclin-dependent kinase 2)-associated protein 1	43.66
17548238	Fabp5	fatty acid binding protein 5, epidermal	43.16
17248539	Gabrb2	gamma-aminobutyric acid (GABA) A receptor, subunit beta 2	42.38
17378896			40.55
17287022	Gm24915	predicted gene, 24915 [Source:MGI Symbol;Acc:MGI:5454692]	38.84
17291355	Acot13	acyl-CoA thioesterase 13	37.17
17308598	Mir687	microRNA 687	36.93
17549910			36.31
17548102	Gm10053	predicted gene 10053 [Source:MGI Symbol;Acc:MGI:3704493]	35.27
17548541	Cycs	cytochrome c, somatic	35.27
17278789	Mir882	microRNA 882	35.17
17366926	Mir466n	microRNA 466n	32.87
17538186	Gm23199	predicted gene, 23199 [Source:MGI Symbol;Acc:MGI:5452976]	32.34
17525548	Ei24	etoposide induced 2.4 mRNA	30.98
17371374	Cers6	ceramide synthase 6	30.13
17415971			29.9
17278852	Mir382	microRNA 382	29.58
17516412	3110039I08Rik	RIKEN cDNA 3110039I08 gene	29.55
17480568	Arrb1	arrestin, beta 1	29.42
17512680	Chtf8	CTF8, chromosome transmission fidelity factor 8	29.38
17326814	Gm25908	predicted gene, 25908 [Source:MGI Symbol;Acc:MGI:5455685]	29.13
17243469	Zfp938	zinc finger protein 938	29.1
17237084	Ccdc59	coiled-coil domain containing 59	28.18
17248894	Snord95	small nucleolar RNA, C/D box 95; guanine nucleotide binding protein (G protein), beta polypeptide 2 like 1	27.98
17248894	Gnb2l1	small nucleolar RNA, C/D box 95; guanine nucleotide binding protein (G protein), beta polypeptide 2 like 1	27.98
17315546	Hnrnpa1	heterogeneous nuclear ribonucleoprotein A1	27.55

17512752	LOC102642963	40S ribosomal protein S26-like; ribosomal protein S26, pseudogene 1 [Source:MGI Symbol;Acc:MGI:3704322]	27.41
17512752	Rps26-ps1	40S ribosomal protein S26-like; ribosomal protein S26, pseudogene 1 [Source:MGI Symbol;Acc:MGI:3704322]	27.41
17289551	Gm16416	predicted gene 16416 [Source:MGI Symbol;Acc:MGI:3646635]	26.74
17547507			26.45
17443752	Cops6	COP9 (constitutive photomorphogenic) homolog, subunit 6 (Arabidopsis thaliana)	26.39
17288145	Habp4	hyaluronic acid binding protein 4	25.51
17391373	1500011K16Rik	RIKEN cDNA 1500011K16 gene	25.11
17499682			25.11
17280867	Mir1938	microRNA 1938	24.76
17465156	Gm24217	predicted gene, 24217 [Source:MGI Symbol;Acc:MGI:5453994]	24.57
17287891			24
17311152	Gm24098	predicted gene, 24098 [Source:MGI Symbol;Acc:MGI:5453875]; predicted gene, 25381 [Source:MGI Symbol;Acc:MGI:5455158]	23.99
17316737	Gm25381	predicted gene, 24098 [Source:MGI Symbol;Acc:MGI:5453875]; predicted gene, 25381 [Source:MGI Symbol;Acc:MGI:5455158]	23.99
17415700	Gm26154	predicted gene, 26154 [Source:MGI Symbol;Acc:MGI:5455931]	23.81
17500441	Purg	purine-rich element binding protein G	23.5
17415177	Rraga	Ras-related GTP binding A	23.26
17544734	Bex1	brain expressed gene 1	22.92
17300247	Trac	T cell receptor alpha constant	22.83
17411133			22.33
17547779	Gm2830	predicted gene 2830 [Source:MGI Symbol;Acc:MGI:3781002]	21.97
17548428	Gm16089	predicted gene 16089 [Source:MGI Symbol;Acc:MGI:3801884]	21.75
17548973			21.7
17458514	Npy	neuropeptide Y	21.57
17504074	Cpne2	copine II	21.08
17465449	Tnpo3	transportin 3	20.73
17212080	Pdcl3	phosducin-like 3	20.59
17499279	Lamp1	lysosomal-associated membrane protein 1	20.45
17364251	Ide	insulin degrading enzyme	20.43
17281748	Trim9	tripartite motif-containing 9	20.2
17364986	Gm24400	predicted gene, 24400 [Source:MGI Symbol;Acc:MGI:5454177]	20.1
17238834	Syne1	spectrin repeat containing, nuclear envelope 1	19.82
17234192	Zwint	ZW10 interactor	19.75
17363107	Zfp91; Cntf; Zfp91Cntf	zinc finger protein 91; ciliary neurotrophic factor; Zfp91-Cntf readthrough transcript (NMD candidate)	19.36
17538161	Prps1	phosphoribosyl pyrophosphate synthetase 1	19.28
17248331	Fbxw11	F-box and WD-40 domain protein 11	19.24
17352580	Ccny	cyclin Y	18.85
17478277	Zdhhc13	zinc finger, DHHC domain containing 13	18.81
17238908	Syne1	spectrin repeat containing, nuclear envelope 1	18.66
17278751	Mir1188	microRNA 1188	18.6
17239401	Pex3	peroxisomal biogenesis factor 3	18.46
17335168	Snrpc	U1 small nuclear ribonucleoprotein C	18.38
17406165	Glrb	glycine receptor, beta subunit	18.24
17506933	Coa6	cytochrome c oxidase assembly factor 6	18.14

17345706	Tomm6; Prickle4	translocase of outer mitochondrial membrane 6 homolog (yeast); prickle homolog 4 (Drosophila)	18.12
17346311	2410015M20Rik	RIKEN cDNA 2410015M20 gene	17.94
17308999	Rps3a2	ribosomal protein S3A2	17.63
17536026	Gm23121	predicted gene, 23121 [Source:MGI Symbol;Acc:MGI:5452898]	17.62
17288650	Mir692-3; Mir692-2	microRNA 692-3; microRNA 692-2	17.54
17378856	Snhg11	small nucleolar RNA host gene 11	17.47
17548525	Hnrnpf	heterogeneous nuclear ribonucleoprotein F	17.29
17523472			17.21
17434105	LOC100041504; Ccl21c; Gm10591; Ccl21b	C-C motif chemokine 21c; chemokine (C-C motif) ligand 21C (leucine) [Source:MGI Symbol;Acc:MGI:1891386]; predicted gene 10591 [Source:MGI Symbol;Acc:MGI:3711256]; chemokine (C-C motif) ligand 21B (leucine) [Source:MGI Symbol;Acc:MGI:1349182]	17.16
17463020	Ing4	inhibitor of growth family, member 4	17.04
17393764	Gm23134	predicted gene, 23134 [Source:MGI Symbol;Acc:MGI:5452911]	16.67
17310530	Cdh10	cadherin 10	16.43
17491541	LOC102640399	uncharacterized LOC102640399	16.41
17530059	Mras	muscle and microspikes RAS	16.33
17286575	Fars2	phenylalanine-tRNA synthetase 2 (mitochondrial)	16.11
17528850	Tmod3	tropomodulin 3	16.05
17279169	Mark3	MAP/microtubule affinity regulating kinase 3	15.57
17521681	Gpx1	glutathione peroxidase 1	15.4
17282268	Gm26777	predicted gene, 26777	15.18
17369948	Mir1954	microRNA 1954	15.13
17318603	Vps28	vacuolar protein sorting 28 (yeast)	14.92
17547859	Gm2573	predicted gene 2573	14.75
17319783	Cyb5r3	cytochrome b5 reductase 3	14.74
17461423	Arl8b	ADP-ribosylation factor-like 8B	14.45
17288898	Lysmd3	LysM, putative peptidoglycan-binding, domain containing 3	14.41
17225760	Stk25	serine/threonine kinase 25 (yeast)	14.15
17290074	Rpl34-ps2	ribosomal protein L34, pseudogene 2 [Source:MGI Symbol;Acc:MGI:3648994]	13.93
17220489	Rab3gap2	RAB3 GTPase activating protein subunit 2	13.89
17242449	Trappc10	trafficking protein particle complex 10	13.86
17519364	Fam214a	family with sequence similarity 214, member A	13.74
17485937			13.69
17242025	Prmt2; Mir678	protein arginine N-methyltransferase 2; microRNA 678	13.64
17327757	Pam16	presequence translocase-asssociated motor 16 homolog (S. cerevisiae)	13.57
17288812	2210408I21Rik	RIKEN cDNA 2210408I21 gene	13.49
17346461	Slc25a23	solute carrier family 25 (mitochondrial carrier; phosphate carrier), member 23	13.34
17296943	Gm10410; Gm8281	predicted gene 10410; predicted pseudogene 8281	13.25
17222156	Kansl3	KAT8 regulatory NSL complex subunit 3	13.22
17436536	Gm1673	predicted gene 1673	12.92
17308731			12.89
17548879	Gm14989	predicted gene 14989 [Source:MGI Symbol;Acc:MGI:3705366]	12.77
17314604	Ccdc184	coiled-coil domain containing 184	12.72
17516960	Cadm1	cell adhesion molecule 1	12.71
17417808	D4Ertd617e	DNA segment, Chr 4, ERATO Doi 617, expressed	12.6
17529928	Gm25607	predicted gene, 25607 [Source:MGI Symbol;Acc:MGI:5455384]	12.59
17259235	Baiap2	brain-specific angiogenesis inhibitor 1-associated protein 2	12.54

17332766	Zdhhc14	zinc finger, DHHC domain containing 14	12.31
17213179	Ndufb3	NADH dehydrogenase (ubiquinone) 1 beta subcomplex 3	12.28
17314693	Tuba1c	tubulin, alpha 1C	12.08
17492160	1810026B05Rik; Chd2	RIKEN cDNA 1810026B05 gene; chromodomain helicase DNA binding protein 2	12.06
17472364	Slco1a4	solute carrier organic anion transporter family, member 1a4	12.02
17383129	Snora17; Snhg7	small nucleolar RNA, H/ACA box 17; small nucleolar RNA host gene 7	11.93
17406921	Gm22935	predicted gene, 22935 [Source:MGI Symbol;Acc:MGI:5452712]	11.9
17480652	Pgm2l1	phosphoglucomutase 2-like 1	11.79
17278688	Mir673	microRNA 673	11.7
17358271	Gm10053	predicted gene 10053 [Source:MGI Symbol;Acc:MGI:3704493]	11.68
17548616	Gm6415	predicted pseudogene 6415 [Source:MGI Symbol;Acc:MGI:3648526]	11.53
17417697	Gm25559	predicted gene, 25559 [Source:MGI Symbol;Acc:MGI:5455336]	11.52
17288948	2810049E08Rik	RIKEN cDNA 2810049E08 gene	11.51
17314910	Larp4	La ribonucleoprotein domain family, member 4	11.51
17282484	Gm5436	predicted pseudogene 5436 [Source:MGI Symbol;Acc:MGI:3643291]	11.39
17484710	B4galnt4	beta-1,4-N-acetyl-galactosaminyl transferase 4	11.32
17467624	Kdm3a	lysine (K)-specific demethylase 3A	11.21
17442970	Vkorc1l1	vitamin K epoxide reductase complex, subunit 1-like 1	11.15
17505367	Txnl4b	thioredoxin-like 4B	11.13
17308603	Itm2b	integral membrane protein 2B	11.09
17354189	Mcc	mutated in colorectal cancers	11.09
17342999	Cpne5	copine V	11.06
17550246			11.01
17540332	Cask	calcium/calmodulin-dependent serine protein kinase (MAGUK family)	10.97
17474983	Cadm4	cell adhesion molecule 4	10.93
17544960	Psmd10	proteasome (prosome, macropain) 26S subunit, non-ATPase, 10	10.89
17414296			10.8
17454049	Gnb2; Mir6418; Epo	guanine nucleotide binding protein (G protein), beta 2; microRNA 6418; erythropoietin	10.54
17243247	Mrpl54	mitochondrial ribosomal protein L54	10.43
17550440			10.4
17452456	Camkk2	calcium/calmodulin-dependent protein kinase kinase 2, beta	10.38
17310808	Dap	death-associated protein	10.38
17322289	Calcoco1	calcium binding and coiled coil domain 1	10.34
17366012	Shtn1	shootin 1	10.3
17233457	Ranbp2	RAN binding protein 2	10.27
17340756	Qk	quaking	10.16
17476641	Gm25817; Gm24766	predicted gene, 25817 [Source:MGI Symbol;Acc:MGI:5455594]; predicted gene, 24766 [Source:MGI Symbol;Acc:MGI:5454543]	10.11
17476646	Gm25817; Gm24766	predicted gene, 25817 [Source:MGI Symbol;Acc:MGI:5455594]; predicted gene, 24766 [Source:MGI Symbol;Acc:MGI:5454543]	10.11
17533690	Araf	v-raf murine sarcoma 3611 viral oncogene homolog	10.1
17305662	Fermt2	fermitin family homolog 2 (Drosophila)	10
17521825	Qrich1	glutamine-rich 1	9.99
17270322	Eftud2	elongation factor Tu GTP binding domain containing 2	9.98
17501544	Npy1r	neuropeptide Y receptor Y1	9.96

17334902	Gm23123	predicted gene, 23123 [Source:MGI Symbol;Acc:MGI:5452900]	9.93
17253376	Sez6	seizure related gene 6	9.87
17242747	R3hdm4	R3H domain containing 4	9.87
17296084	Zswim6	zinc finger SWIM-type containing 6	9.86
17342167	Gnptg	N-acetylglucosamine-1-phosphotransferase, gamma subunit	9.84
17332228	Rcan1	regulator of calcineurin 1	9.82
17527806	Rps11-ps1	ribosomal protein S11, pseudogene 1	9.77
17447504	Gm24878	predicted gene, 24878 [Source:MGI Symbol;Acc:MGI:5454655]	9.74
17365072	Bloc1s2	biogenesis of lysosomal organelles complex-1, subunit 2	9.72
17434095	LOC100041536; Gm13303	protein FAM205A-like; predicted gene 13303 [Source:MGI Symbol;Acc:MGI:3705775]	9.7
17287437	Simc1	SUMO-interacting motifs containing 1	9.69
17278882	Mir377	microRNA 377	9.62
17373482	Mir1955	microRNA 1955	9.62
17500808	Gm6180	predicted pseudogene 6180 [Source:MGI Symbol;Acc:MGI:3643972]	9.59
17322827	Gm22932	predicted gene, 22932 [Source:MGI Symbol;Acc:MGI:5452709]	9.59
17550052			9.59
17323110	2900011O08Rik	RIKEN cDNA 2900011O08 gene	9.5
17437350	Gm20475	predicted gene 20475 [Source:MGI Symbol;Acc:MGI:5141940]	9.48
17540011	Wdr13	WD repeat domain 13	9.46
17431451	Gm15979	predicted gene 15979 [Source:MGI Symbol;Acc:MGI:3801882]	9.37
17315349	Mfsd5	major facilitator superfamily domain containing 5	9.35
17318923	Cacng2	calcium channel, voltage-dependent, gamma subunit 2	9.27
17515248	Carm1	coactivator-associated arginine methyltransferase 1	9.26
17483337	Srcap; Tmem265	Snf2-related CREBBP activator protein; transmembrane protein 265	9.15
17413090	Gm13308; Gm20878; Gm21586	predicted gene 13308; predicted gene, 20878; predicted gene, 21586	9.1
17413103	Gm13308; Gm20878; Gm21586	predicted gene 13308; predicted gene, 20878; predicted gene, 21586	9.1
17525892	Gm25401	predicted gene, 25401 [Source:MGI Symbol;Acc:MGI:5455178]	9.05
17330373	Adprh	ADP-ribosylarginine hydrolase	9.05
17243604	Nuak1	NUAK family, SNF1-like kinase, 1	8.98
17449958	Mir703	microRNA 703	8.96
17481723	Snora23	small nucleolar RNA, H/ACA box 23	8.96
17550288			8.95
17443430	Polr2j	polymerase (RNA) II (DNA directed) polypeptide J	8.93
17382013	Dnajc1; Gm39761	DnaJ (Hsp40) homolog, subfamily C, member 1; predicted gene, 39761	8.89
17406031	Rapgef2	Rap guanine nucleotide exchange factor (GEF) 2	8.62
17253810	Rab11fip4	RAB11 family interacting protein 4 (class II)	8.6
17490481	Cpt1c	carnitine palmitoyltransferase 1c	8.6
17344472	Atat1	alpha tubulin acetyltransferase 1	8.59
17539303	Phka2	phosphorylase kinase alpha 2	8.55
17353923	Pcdh1	protocadherin 1	8.47
17253108	Ywhae	tyrosine 3-monooxygenase/tryptophan 5-monooxygenase activation protein, epsilon polypeptide	8.44
17324762	Rnf168	ring finger protein 168	8.16
17493034			8.14
17360068	Wbp1l	WW domain binding protein 1 like	7.92

17226435	Lypd1	Ly6/Plaur domain containing 1	7.91
17477424	Snord88c	small nucleolar RNA, C/D box 88C	7.89
17503181	Trmt1	tRNA methyltransferase 1	7.88
17429886	Maneal	mannosidase, endo-alpha-like	7.82
17269246	Krtap4-13	keratin associated protein 4-13	7.75
17343897	Ppt2	palmitoyl-protein thioesterase 2	7.74
17281582	Sos2	son of sevenless homolog 2 (Drosophila)	7.73
17420373	Eif4g3; Gm32394	eukaryotic translation initiation factor 4 gamma, 3; predicted gene, 32394	7.61
17490050	Zfp141	zinc finger protein 141	7.59
17449647	Gm15710	predicted gene 15710 [Source:MGI Symbol;Acc:MGI:3783151]	7.57
17340084	Dync2li1	dynein cytoplasmic 2 light intermediate chain 1	7.54
17399333	Gba	glucosidase, beta, acid	7.5
17457802	Trbj2-7; Tcrb-J; Trbv13-2; Trbv29	T cell receptor beta joining 2-7; T cell receptor beta, joining region; T cell receptor beta, variable 13-2; T cell receptor beta, variable 29	7.38
17311199	Dcaf13	DDB1 and CUL4 associated factor 13	7.34
17535390	Prrg3	proline rich Gla (G-carboxyglutamic acid) 3 (transmembrane)	7.31
17280897	Stxbp6; Gm38487	syntaxin binding protein 6 (amisyn); predicted gene, 38487	7.29
17517914	Nptn	neuroplastin	7.26
17366918	Mir466d	microRNA 466d	7.15
17510922	Pkn1	protein kinase N1	7.09
17512151			7.08
17243392	Gna11	guanine nucleotide binding protein, alpha 11	7.06
17302289	Pcdh17	protocadherin 17	7
17257835	Amz2	archaelysin family metallopeptidase 2	6.99
17232444	Hddc2	HD domain containing 2	6.96
17312127	Ptp4a3	protein tyrosine phosphatase 4a3	6.96
17315713	Atp5h	ATP synthase, H+ transporting, mitochondrial F0 complex, subunit D	6.95
17473439	Zfp524	zinc finger protein 524	6.94
17433602	Tprgl	transformation related protein 63 regulated like	6.93
17379011	Top1	topoisomerase (DNA) I	6.91
17335616	Zfand3	zinc finger, AN1-type domain 3	6.88
17268755	Med1	mediator complex subunit 1	6.83
17488752	Catsperg1	catsper channel auxiliary subunit gamma 1	6.8
17301718	Egr3	early growth response 3	6.79
17307080	Pspc1	paraspeckle protein 1	6.79
17246672	Gm24013	predicted gene, 24013 [Source:MGI Symbol;Acc:MGI:5453790]	6.77
17491497	C230091D08Rik	RIKEN cDNA C230091D08 gene	6.71
17457876	Gstk1	glutathione S-transferase kappa 1	6.71
17519384	Arpp19	cAMP-regulated phosphoprotein 19	6.69
17284114	Ckb	creatine kinase, brain	6.64
17248691	Ebf1	early B cell factor 1	6.62
17269194	Gm14190	predicted gene 14190 [Source:MGI Symbol;Acc:MGI:3651811]	6.56
17353171	Tpgs2	tubulin polyglutamylase complex subunit 2	6.51
17525894	Sorl1	sortilin-related receptor, LDLR class A repeats-containing	6.51
17543795	Ftx	Ftx transcript, Xist regulator (non-protein coding)	6.43
17365070	Gm24336	predicted gene, 24336 [Source:MGI Symbol;Acc:MGI:5454113]	6.38
17238936	Syne1	spectrin repeat containing, nuclear envelope 1	6.37
17342483	Fam195a	family with sequence similarity 195, member A	6.32
17318403	Gm25720	predicted gene, 25720 [Source:MGI Symbol;Acc:MGI:5455497]	6.28

17400875	Zfp697	zinc finger protein 697	6.27
17300153	Traj50	T cell receptor alpha joining 50	6.26
17262521	Sec24a	Sec24 related gene family, member A (S. cerevisiae)	6.25
17271257	Gm22378	predicted gene, 22378 [Source:MGI Symbol;Acc:MGI:5452155]	6.25
17519965	Gm25125	predicted gene, 25125 [Source:MGI Symbol;Acc:MGI:5454902]	6.24
17361099	Aip	aryl-hydrocarbon receptor-interacting protein	6.21
17392572	Cd93	CD93 antigen	6.21
17279835	Gm17541	predicted gene, 17541 [Source:MGI Symbol;Acc:MGI:4937175]	6.21
17273059	LOC105246895; Gm11772	uncharacterized LOC105246895; predicted gene 11772 [Source:MGI Symbol;Acc:MGI:3649468]	6.2
17489645	4931406P16Rik	RIKEN cDNA 4931406P16 gene	6.2
17515090	Icam5	intercellular adhesion molecule 5, telencephalin	6.16
17258947	Cbx2	chromobox 2	6.15
17503043	Samd1	sterile alpha motif domain containing 1	6.15
17286670	Snrnp48	small nuclear ribonucleoprotein 48 (U11/U12)	6.08
17511014	Gm10643	predicted gene 10643 [Source:MGI Symbol;Acc:MGI:3642507]	6.04
17314085	Ppp6r2	protein phosphatase 6, regulatory subunit 2	6.03
17273652	Dnmt3a	DNA methyltransferase 3A	6.03
17236739	Ube2n	ubiquitin-conjugating enzyme E2N	6.02
17364474	Pdlim1	PDZ and LIM domain 1 (elfin)	5.99
17512181	Tk2	thymidine kinase 2, mitochondrial	5.98
17489420	Fxyd1	FXYD domain-containing ion transport regulator 1	5.94
17398903	Nes	nestin	5.93
17380340	Nelfcd	negative elongation factor complex member C/D, Th1l	5.91
17492943	Mesdc1	mesoderm development candidate 1	5.91
17299518	Parp2	poly (ADP-ribose) polymerase family, member 2	5.91
17302356	Gm24770	predicted gene, 24770 [Source:MGI Symbol;Acc:MGI:5454547]	5.9
17489713	Lrp3	low density lipoprotein receptor-related protein 3	5.89
17526382	Kmt2a	lysine (K)-specific methyltransferase 2A	5.88
17233663	Mir466j	microRNA 466j	5.82
17223277	Gm6644	Akr1b3 pseudogene	5.82
17424850	Rnf38	ring finger protein 38	5.81
17445568	Dmtf1	cyclin D binding myb-like transcription factor 1	5.77
17292426	1110007C09Rik	RIKEN cDNA 1110007C09 gene	5.72
17498760	Evi5l	ecotropic viral integration site 5 like	5.69
17210887	Atp6v1h	ATPase, H+ transporting, lysosomal V1 subunit H	5.67
17467963	6330415B21Rik	RIKEN cDNA 6330415B21 gene	5.62
17373984	Immp1l	IMP1 inner mitochondrial membrane peptidase-like (S. cerevisiae)	5.58
17235757	Tle2	transducin-like enhancer of split 2, homolog of Drosophila E(spl)	5.57
17464503	2810474O19Rik	RIKEN cDNA 2810474O19 gene	5.55
17237952	F420014N23Rik	RIKEN cDNA F420014N23 gene	5.55
17291767	Tubb2a	tubulin, beta 2A class IIA	5.54
17475231	Tmem145	transmembrane protein 145	5.51
17211294	Gdap1	ganglioside-induced differentiation-associated-protein 1	5.51
17281607	Gm24449	predicted gene, 24449 [Source:MGI Symbol;Acc:MGI:5454226]	5.46
17338571	Kat2b	K(lysine) acetyltransferase 2B	5.44
17529307	Elovl4	elongation of very long chain fatty acids (FEN1/Elo2, SUR4/Elo3, yeast)-like 4	5.4

17216010	Gm17415	predicted gene, 17415 [Source:MGI Symbol;Acc:MGI:4937049]	5.38
17441540	Med13l	mediator complex subunit 13-like	5.35
17472983	Gm23456	predicted gene, 23456 [Source:MGI Symbol;Acc:MGI:5453233]	5.32
17211850	Cox5b	cytochrome c oxidase subunit Vb	5.31
17263011	Glra1	glycine receptor, alpha 1 subunit	5.3
17316019	Gm24844	predicted gene, 24844 [Source:MGI Symbol;Acc:MGI:5454621]	5.3
17519394	Myo5a	myosin VA	5.28
17373328	Ckap5	cytoskeleton associated protein 5	5.27
17345422	Cul9	cullin 9	5.27
17483061	Sgf29	SAGA complex associated factor 29	5.26
17499849	Alg11	asparagine-linked glycosylation 11 (alpha-1,2-mannosyltransferase)	5.25
17303623			5.24
17416811	Mir761	microRNA 761	5.22
17326250	Zbtb11	zinc finger and BTB domain containing 11	5.19
17319339	Npcd; Cbx6; Nptxr	neuronal pentraxin chromo domain; chromobox 6; neuronal pentraxin receptor	5.17
17411547	Chchd7	coiled-coil-helix-coiled-coil-helix domain containing 7	5.14
17336906	Ddah2	dimethylarginine dimethylaminohydrolase 2	5.12
17514355	Gm10715	predicted gene 10715 [Source:MGI Symbol;Acc:MGI:3642376]	5.09
17258653	Mettl23; Mfsd11	methyltransferase like 23; major facilitator superfamily domain containing 11	5.07
17323082	Ntan1	N-terminal Asn amidase	5.05
17526861	Dlat	dihydrolipoamide S-acetyltransferase (E2 component of pyruvate dehydrogenase complex)	5.04
17547933			5.02
17233699	Rpl27a-ps1	ribosomal protein L27A, pseudogene 1 [Source:MGI Symbol;Acc:MGI:3645354]	5.02
17221177	Vcpip1	valosin containing protein (p97)/p47 complex interacting protein 1	5.02
17222788			4.99
17423901	Zfp292	zinc finger protein 292	4.97
17430777	Gmeb1	glucocorticoid modulatory element binding protein 1	4.97
17366932	Mir466h	microRNA 466h	4.95
17406752	Slc25a44	solute carrier family 25, member 44	4.94
17282171	Zfyve26	zinc finger, FYVE domain containing 26	4.93
17266095	Nxn; Gm36384	nucleoredoxin; predicted gene, 36384	4.93
17235470	Sppl2b	signal peptide peptidase like 2B	4.91
17404616	Mrpl47	mitochondrial ribosomal protein L47	4.87
17409953	Gm4609	predicted gene 4609 [Source:MGI Symbol;Acc:MGI:3782792]	4.87
17321351	Prkag1	protein kinase, AMP-activated, gamma 1 non-catalytic subunit	4.86
17257146	Rprml	reprimo-like	4.85
17431049	Sfn	stratifin	4.85
17506230	6430548M08Rik	RIKEN cDNA 6430548M08 gene	4.85
17283422	Atxn3	ataxin 3	4.84
17530742	Tex264	testis expressed gene 264	4.79
17514601	Gm29376	predicted gene 29376	4.78
17513886	Vps9d1	VPS9 domain containing 1	4.74
17370804	Bloc1s2-ps	biogenesis of lysosomal organelles complex-1, subunit 2, pseudogene	4.72
17287081	Id4	inhibitor of DNA binding 4	4.71

17516558	H2afx	H2A histone family, member X	4.7
17460958	Ccdc174	coiled-coil domain containing 174	4.7
17361073	Gstp1	glutathione S-transferase, pi 1	4.65
17471274	Gm10010	predicted gene 10010 [Source:MGI Symbol;Acc:MGI:3641978]	4.65
17435180	Lhfpl3	lipoma HMGIC fusion partner-like 3	4.65
17255907	Rpl19	ribosomal protein L19	4.63
17327035	Olig1	oligodendrocyte transcription factor 1	4.63
17328756	Med15	mediator complex subunit 15	4.63
17250330	Rai1	retinoic acid induced 1	4.59
17399302	Scamp3	secretory carrier membrane protein 3	4.59
17230273	4930527J03Rik	RIKEN cDNA 4930527J03 gene	4.58
17485553	Tmc4	transmembrane channel-like gene family 4	4.57
17222568	Creg2	cellular repressor of E1A-stimulated genes 2	4.55
17248196	Asb3	ankyrin repeat and SOCS box-containing 3	4.54
17499480	Kbtbd11	kelch repeat and BTB (POZ) domain containing 11	4.53
17516613			4.51
17498080	Gm10013	predicted gene 10013	4.51
17530348	Dnajc13	DnaJ (Hsp40) homolog, subfamily C, member 13	4.51
17391899	Adra1d	adrenergic receptor, alpha 1d	4.51
17257937	Kcnj2	potassium inwardly-rectifying channel, subfamily J, member 2	4.5
17495851	Usp31	ubiquitin specific peptidase 31	4.48
17339079	Gm24813	predicted gene, 24813 [Source:MGI Symbol;Acc:MGI:5454590]	4.47
17520490	Atr	ataxia telangiectasia and Rad3 related	4.46
17254916	Srsf1	serine/arginine-rich splicing factor 1	4.46
17311441	A930017M01Rik	Smg-5 homolog, nonsense mediated mRNA decay factor pseudogene	4.45
17524994	Rp9	retinitis pigmentosa 9 (human)	4.44
17530967	Slc38a3	solute carrier family 38, member 3	4.43
17472063	Hebp1	heme binding protein 1	4.43
17254366	Synrg	synergin, gamma	4.42
17371939			4.4
17431880	Pqlc2	PQ loop repeat containing 2	4.38
17394318	Zfp335	zinc finger protein 335	4.38
17328870	Zdhhc8	zinc finger, DHHC domain containing 8	4.37
17385684	Rbms1	RNA binding motif, single stranded interacting protein 1	4.35
17531168	Dag1	dystroglycan 1	4.35
17362689	Sdhaf2	succinate dehydrogenase complex assembly factor 2	4.34
17394102	Kcns1	K+ voltage-gated channel, subfamily S, 1	4.34
17457796	Tcrb-J; Trbj2-4	T cell receptor beta, joining region; T cell receptor beta joining 2-4	4.33
17483802	Wdr11	WD repeat domain 11	4.32
17374406	BC052040	cDNA sequence BC052040	4.31
17497811	Mir210	microRNA 210	4.3
17241420	Kif1bp	KIF1 binding protein	4.29
17231248	G0s2	G0/G1 switch gene 2	4.29
17548277			4.28
17548726	Gm7265	predicted gene 7265	4.25
17451128	Pus1	pseudouridine synthase 1	4.25
17532030	Cmc1	COX assembly mitochondrial protein 1	4.24
17513209	Kars	lysyl-tRNA synthetase	4.24
17474377	Nova2	neuro-oncological ventral antigen 2	4.23
17366623	Gm13389	predicted gene 13389 [Source:MGI Symbol;Acc:MGI:3649902]	4.21
17463709	Crebl2	cAMP responsive element binding protein-like 2	4.2
17266903	Gas2l2	growth arrest-specific 2 like 2	4.16

17274249	Pdia6	protein disulfide isomerase associated 6	4.16
17385374	Nr4a2	nuclear receptor subfamily 4, group A, member 2	4.15
17222876	Gm8420	predicted gene 8420 [Source:MGI Symbol;Acc:MGI:3645594]	4.15
17360084	As3mt	arsenic (+3 oxidation state) methyltransferase	4.15
17282008	Zbtb25	zinc finger and BTB domain containing 25	4.14
17358210	Mir204	microRNA 204	4.11
17457555	Agk	acylglycerol kinase	4.1
17236487			4.09
17286998	Rbm24	RNA binding motif protein 24	4.09
17319465	St13	suppression of tumorigenicity 13	4.08
17515062	Mrpl4	mitochondrial ribosomal protein L4	4.07
17333188	1700110C19Rik	RIKEN cDNA 1700110C19 gene	4.07
17333076	Gm8465	predicted gene 8465	4.06
17253991	Cdk5r1	cyclin-dependent kinase 5, regulatory subunit 1 (p35)	4.05
17354810	Afap1l1	actin filament associated protein 1-like 1	4.04
17388215	1110051M20Rik	RIKEN cDNA 1110051M20 gene	4.04
17321740	Pou6f1	POU domain, class 6, transcription factor 1	4.04
17235890	Hcfc2	host cell factor C2	4.04
17464367	Ppfibp1	PTPRF interacting protein, binding protein 1 (liprin beta 1)	4.03
17417195	Cyp4a12b	cytochrome P450, family 4, subfamily a, polypeptide 12B	4.03
17287175	Ogn	osteoglycin	4.02
17245188	Frs2	fibroblast growth factor receptor substrate 2	4.01
17474268	Gm25068	predicted gene, 25068 [Source:MGI Symbol;Acc:MGI:5454845]	4
17548890	Gm4997	predicted gene 4997 [Source:MGI Symbol;Acc:MGI:3643651]	3.97
17287576			3.97
17404570	Slc7a14	solute carrier family 7 (cationic amino acid transporter, y+ system), member 14	3.97
17350166	Gm8181	predicted gene 8181 [Source:MGI Symbol;Acc:MGI:3643329]	3.96
17540378	Maob	monoamine oxidase B	3.96
17311807	Sqle	squalene epoxidase	3.95
17393032	Tspyl3	TSPY-like 3	3.94
17230256	Adss	adenylosuccinate synthetase, non muscle	3.92
17245202	Yeats4	YEATS domain containing 4	3.92
17279764	Efr3b	EFR3 homolog B (S. cerevisiae)	3.92
17444047	Elfn1	leucine rich repeat and fibronectin type III, extracellular 1	3.9
17269498	Nt5c3b	5-nucleotidase, cytosolic IIIB	3.9
17341679	Gm25092	predicted gene, 25092 [Source:MGI Symbol;Acc:MGI:5454869]	3.89
17547552			3.87
17476528	Sbsn	suprabasin	3.86
17514603	Tmem123	transmembrane protein 123	3.85
17231287	Cd46	CD46 antigen, complement regulatory protein	3.85
17365160	Mrpl43	mitochondrial ribosomal protein L43	3.85
17513534	1190005I06Rik	RIKEN cDNA 1190005I06 gene	3.85
17451043	Noc4l	nucleolar complex associated 4 homolog (S. cerevisiae)	3.83
17531671	Tmie	transmembrane inner ear	3.8
17444364	Fam220a	family with sequence similarity 220, member A	3.79
17255775	Socs7	suppressor of cytokine signaling 7	3.78
17230231	Akt3	thymoma viral proto-oncogene 3	3.78
17286487	Bphl	biphenyl hydrolase-like (serine hydrolase, breast epithelial mucin-associated antigen)	3.77
17478789	Ndn	necdin	3.77

17491642	Snord107; Gm38393	small nucleolar RNA, C/D box 107 [Source:MGI Symbol;Acc:MGI:4358283]; predicted gene, 38393 [Source:MGI Symbol;Acc:MGI:5613898]	3.77
17500532	Gm4889	predicted gene 4889 [Source:MGI Symbol;Acc:MGI:3647233]	3.77
17358138	Tmem2	transmembrane protein 2	3.76
17219286	Dedd	death effector domain-containing	3.74
17518312	Snord16a	small nucleolar RNA, C/D box 16A	3.73
17528274	Plekho2	pleckstrin homology domain containing, family O member 2	3.73
17300390	Mhrt	myosin heavy chain associated RNA transcript	3.72
17373155	Psmc3; Gm39875	proteasome (prosome, macropain) 26S subunit, ATPase 3; predicted gene, 39875	3.72
17520396	Slc9a9	solute carrier family 9 (sodium/hydrogen exchanger), member 9	3.71
17268576	Gm11613	predicted gene 11613 [Source:MGI Symbol;Acc:MGI:3651819]	3.7
17298088	Gm22485	predicted gene, 22485 [Source:MGI Symbol;Acc:MGI:5452262]	3.66
17499038	Arhgef7	Rho guanine nucleotide exchange factor (GEF7)	3.66
17442328	Gm24682	predicted gene, 24682 [Source:MGI Symbol;Acc:MGI:5454459]	3.65
17454590	Iqce	IQ motif containing E	3.64
17366359	Fam171a1	family with sequence similarity 171, member A1	3.63
17270703	Cyb561	cytochrome b-561	3.63
17422662	Ssu72	Ssu72 RNA polymerase II CTD phosphatase homolog (yeast)	3.61
17444398	Eif2ak1	eukaryotic translation initiation factor 2 alpha kinase 1	3.6
17231680	Gm22546	predicted gene, 22546 [Source:MGI Symbol;Acc:MGI:5452323]	3.59
17346185	Dpp9	dipeptidylpeptidase 9	3.59
17280452	Sntg2	syntrophin, gamma 2	3.58
17270721	Gm11646	predicted gene 11646 [Source:MGI Symbol;Acc:MGI:3649612]	3.58
17367683	Gm13411	predicted gene 13411 [Source:MGI Symbol;Acc:MGI:3651013]	3.58
17388406	Slc35c1	solute carrier family 35, member C1	3.57
17475685	Zfp60	zinc finger protein 60	3.57
17310404	Zfr	zinc finger RNA binding protein	3.54
17407396	Adrm1	adhesion regulating molecule 1	3.51
17539626	Trappc2	trafficking protein particle complex 2	3.49
17255335	Samd14	sterile alpha motif domain containing 14	3.49
17519533	Lysmd2	LysM, putative peptidoglycan-binding, domain containing 2	3.49
17323968	Vwa5b2	von Willebrand factor A domain containing 5B2	3.48
17297490	Fut11	fucosyltransferase 11	3.46
17283532	9330161L09Rik	RIKEN cDNA 9330161L09 gene [Source:MGI Symbol;Acc:MGI:1924475]	3.45
17250549	Kcnj12	potassium inwardly-rectifying channel, subfamily J, member 12	3.45
17230166	Opn3	opsin 3	3.44
17548367	Gm13249	predicted gene 13249	3.44
17466386	Fam131b	family with sequence similarity 131, member B	3.42
17531900	Cnot10	CCR4-NOT transcription complex, subunit 10	3.42
17303353	Gm3558; Gm10406; Gm5796	predicted gene 3558; predicted gene 10406; predicted gene 5796	3.39
17262478	Hnrnpab	heterogeneous nuclear ribonucleoprotein A/B	3.38
17529290	Lca5	Leber congenital amaurosis 5 (human)	3.38
17488352	Psmc4	proteasome (prosome, macropain) 26S subunit, ATPase, 4	3.38

17547656			3.37
17509146	Snx25	sorting nexin 25	3.35
17399314	Fam189b	family with sequence similarity 189, member B	3.35
17427769	Pcsk9	proprotein convertase subtilisin/kexin type 9	3.33
17519009	Gtf2a2	general transcription factor II A, 2	3.32
17333672	Riok2	RIO kinase 2 (yeast)	3.31
17320583	Cpne8	copine VIII	3.31
17349181	Wdr33	WD repeat domain 33	3.31
17458718	Prr15	proline rich 15	3.3
17222878	Gm3940	predicted gene 3940 [Source:MGI Symbol;Acc:MGI:3782114]	3.29
17510280	Nr2f6	nuclear receptor subfamily 2, group F, member 6	3.29
17289860	Gm15323; Gm15322	predicted gene 15323; predicted gene 15322	3.28
17550422			3.28
17280247	LOC101056074	zinc finger protein 124-like	3.27
17360331	Mxi1	MAX interactor 1, dimerization protein	3.27
17451223	Sez6l	seizure related 6 homolog like	3.26
17324998	Osbpl11	oxysterol binding protein-like 11	3.25
17349634	Cystm1	cysteine-rich transmembrane module containing 1	3.25
17483891	Plekha1	pleckstrin homology domain containing, family A (phosphoinositide binding specific) member 1	3.24
17506249	Gse1	genetic suppressor element 1	3.23
17425160	Erp44	endoplasmic reticulum protein 44	3.23
17238150	Zbtb39	zinc finger and BTB domain containing 39	3.23
17394349	Ncoa5	nuclear receptor coactivator 5	3.22
17482021	Pde3b	phosphodiesterase 3B, cGMP-inhibited	3.22
17360004	Tmem180; Gm36493	transmembrane protein 180; predicted gene, 36493	3.22
17498699	Stxbp2	syntaxin binding protein 2	3.22
17345830	Daam2	dishevelled associated activator of morphogenesis 2	3.21
17409721	Hiat1	hippocampus abundant gene transcript 1	3.2
17380555	Gm14403	predicted gene 14403	3.2
17423939	Lingo2	leucine rich repeat and Ig domain containing 2	3.19
17231619	Gm10944	predicted gene 10944 [Source:MGI Symbol;Acc:MGI:3779154]	3.19
17550192			3.18
17527988	Spesp1	sperm equatorial segment protein 1	3.18
17338103	Crip3	cysteine-rich protein 3	3.16
17263506	Med9os	mediator complex subunit 9, opposite strand	3.15
17261016	Commd1	COMM domain containing 1	3.15
17301478	Trim35	tripartite motif-containing 35	3.15
17310816	Cmbl	carboxymethylenebutenolidase-like (Pseudomonas)	3.14
17414482	Snx30	sorting nexin family member 30	3.14
17391670	Fastkd5; Ubox5	FAST kinase domains 5; U box domain containing 5	3.13
17226489	Map3k19	mitogen-activated protein kinase kinase kinase 19	3.13
17251516	Gm25371	predicted gene, 25371 [Source:MGI Symbol;Acc:MGI:5455148]	3.12
17548587			3.12
17515058	Ppan	peter pan homolog (Drosophila)	3.11
17256323	Krtap31-1	keratin associated protein 31-1	3.11
17368171	Bmyc	brain expressed myelocytomatosis oncogene	3.11
17492803	BC048679	cDNA sequence BC048679	3.11
17284426	Ighv2-9	immunoglobulin heavy variable 2-9	3.1
17511927	Kifc3	kinesin family member C3	3.1
17268210	Ngfr	nerve growth factor receptor (TNFR superfamily, member 16)	3.1
17474529	Gpr4	G protein-coupled receptor 4	3.1
17487249	Mark4	MAP/microtubule affinity regulating kinase 4	3.09

17502280	Colgalt1	collagen beta(1-O)galactosyltransferase 1	3.09
17418560	Eva1b	eva-1 homolog B (C. elegans)	3.09
17351482			3.09
17334275	Caskin1	CASK interacting protein 1	3.08
17293258	Spata31d1a	spermatogenesis associated 31 subfamily D, member 1A	3.08
17287889	Gm24284	predicted gene, 24284 [Source:MGI Symbol;Acc:MGI:5454061]	3.07
17491699	Mir344d-2	microRNA 344d-2	3.07
17463673	Etv6	ets variant 6	3.07
17288370	Fastkd3	FAST kinase domains 3	3.07
17431772	Vwa5b1	von Willebrand factor A domain containing 5B1	3.07
17473155	Cacng7	calcium channel, voltage-dependent, gamma subunit 7	3.07
17435660	Nom1	nucleolar protein with MIF4G domain 1	3.06
17313322	L3mbtl2	l(3)mbt-like 2 (Drosophila)	3.06
17389736	Ankrd63	ankyrin repeat domain 63	3.05
17499087			3.05
17424674	Tln1	talin 1	3.04
17324576	Hrasls	HRAS-like suppressor	3.04
17349884	Pcdhb6	protocadherin beta 6	3.03
17284645	Ighv1-75	immunoglobulin heavy variable 1-75	3.03
17485943	Shisa7	shisa family member 7	3.02
17266146	Gosr1	golgi SNAP receptor complex member 1	3.02
17503397	Man2b1	mannosidase 2, alpha B1	3.02
17430997	Wdtc1	WD and tetratricopeptide repeats 1	3.02
17491454	Nipa1	non imprinted in Prader-Willi/Angelman syndrome 1 homolog (human)	3.01
17332133	Dnajc28	DnaJ (Hsp40) homolog, subfamily C, member 28	3
17436237	Fosl2	fos-like antigen 2	3
17309905	Snord72	small nucleolar RNA, C/D box 72	2.99
17221093	Gm6104	predicted gene 6104 [Source:MGI Symbol;Acc:MGI:3648587]	2.99
17480041	AI314278	expressed sequence AI314278	2.98
17323627	Vpreb2	pre-B lymphocyte gene 2	2.98
17549488			2.98
17362717	Tkfc	triokinase, FMN cyclase	2.97
17432783	Zfp933	zinc finger protein 933	2.96
17309347	Gm4775	predicted gene 4775 [Source:MGI Symbol;Acc:MGI:3647362]	2.96
17318428	Oplah	5-oxoprolinase (ATP-hydrolysing)	2.94
17242316	Gm3238	predicted gene 3238	2.93
17240971	Edar	ectodysplasin-A receptor	2.93
17452741	Pitpnm2	phosphatidylinositol transfer protein, membrane-associated 2	2.93
17444930	Gm15409	predicted gene 15409 [Source:MGI Symbol;Acc:MGI:3705229]	2.92
17489039	Lrfn3; Gm42382	leucine rich repeat and fibronectin type III domain containing 3; predicted gene, 42382	2.92
17485849	Tmem86b	transmembrane protein 86B	2.92
17504878	Rltpr	RGD motif, leucine rich repeats, tropomodulin domain and proline-rich containing	2.91
17356356	Rin1	Ras and Rab interactor 1	2.9
17359008	5-Mar	membrane-associated ring finger (C3HC4) 5	2.9
17548369	LOC102637947; Gm13035	60S acidic ribosomal protein P1-like; predicted gene 13035 [Source:MGI Symbol;Acc:MGI:3650251]	2.9
17529218	Htr1b	5-hydroxytryptamine (serotonin) receptor 1B	2.89
17221923	n-R5s209	nuclear encoded rRNA 5S 209 [Source:MGI Symbol;Acc:MGI:4422074]	2.89
17488001	Ccdc97	coiled-coil domain containing 97	2.89

17217373	Fmod	fibromodulin	2.88
17388284	Mdk	midkine	2.88
17453383	Clip2	CAP-GLY domain containing linker protein 2	2.88
17437850	LOC102635521; Gm26725	uncharacterized LOC102635521; predicted gene, 26725 [Source:MGI Symbol;Acc:MGI:5477219]	2.87
17288946	Mir9-2; C130071C03Rik	microRNA 9-2; RIKEN cDNA C130071C03 gene	2.87
17422859	Tnfrsf4	tumor necrosis factor receptor superfamily, member 4	2.87
17477930	Rasip1; Izumo1	Ras interacting protein 1; izumo sperm-egg fusion 1	2.86
17381752	Fam188a	family with sequence similarity 188, member A	2.86
17329527	Mb21d2	Mab-21 domain containing 2	2.86
17357213	Zbtb3	zinc finger and BTB domain containing 3	2.85
17211090	Cspp1	centrosome and spindle pole associated protein 1	2.85
17247158	Ccm2	cerebral cavernous malformation 2	2.85
17230127	Grem2	gremlin 2 homolog, cysteine knot superfamily (Xenopus laevis)	2.84
17432790	Miip	migration and invasion inhibitory protein	2.84
17540584	Gm26593	predicted gene, 26593 [Source:MGI Symbol;Acc:MGI:5477087]	2.84
17275869			2.84
17350088			2.84
17393110	Cdk5rap1	CDK5 regulatory subunit associated protein 1	2.83
17518342	Megf11	multiple EGF-like-domains 11	2.83
17512088	Slc38a7	solute carrier family 38, member 7	2.83
17213548	Nrp2	neuropilin 2	2.82
17494117	Trim68	tripartite motif-containing 68	2.82
17483048	Apobr	apolipoprotein B receptor	2.81
17462872	C1ra	complement component 1, r subcomponent A	2.81
17447451	Tbc1d14	TBC1 domain family, member 14	2.81
17520063	Mrap2	melanocortin 2 receptor accessory protein 2	2.8
17493632	Dgat2	diacylglycerol O-acyltransferase 2	2.8
17333946	Zfp948	zinc finger protein 948	2.79
17447610	Msx1	msh homeobox 1	2.79
17383173	Surf1	surfeit gene 1	2.79
17424263	Fam219a	family with sequence similarity 219, member A	2.79
17333709	Spaca6	sperm acrosome associated 6	2.78
17486223	Vmn2r33	vomeronasal 2, receptor 33	2.78
17400072	Tdrkh	tudor and KH domain containing protein	2.78
17327262			2.77
17415764	LOC102643030	uncharacterized LOC102643030	2.77
17343568	Zfp101	zinc finger protein 101	2.77
17547016	Gm37044; Gm38369	predicted gene, 37044 [Source:MGI Symbol;Acc:MGI:5610272]; predicted gene, 38369 [Source:MGI Symbol;Acc:MGI:5611597]	2.76
17284554	Ighv1-37	immunoglobulin heavy variable 1-37	2.75
17363156	Gm22323	predicted gene, 22323 [Source:MGI Symbol;Acc:MGI:5452100]	2.75
17363158	Gm23931	predicted gene, 23931 [Source:MGI Symbol;Acc:MGI:5453708]	2.75
17411352	Tyw3	tRNA-yW synthesizing protein 3 homolog (S. cerevisiae)	2.75
17334113	9530082P21Rik	RIKEN cDNA 9530082P21 gene	2.75
17290301	Akr1e1	aldo-keto reductase family 1, member E1	2.75
17345666	Gm16494	predicted gene 16494 [Source:MGI Symbol;Acc:MGI:3641930]	2.74
17530669	Acy1	aminoacylase 1	2.72
17248470	Gm26070	predicted gene, 26070 [Source:MGI Symbol;Acc:MGI:5455847]	2.72

17317930	Mroh5	maestro heat-like repeat family member 5	2.71
17545291	Tmem29	transmembrane protein 29	2.71
17248406	Gm12121	predicted gene 12121	2.7
17270303	Psmb5-ps	proteasome (prosome, macropain) subunit, beta type 5, pseudogene	2.7
17212406	Bivm	basic, immunoglobulin-like variable motif containing	2.69
17244439	Plxnc1	plexin C1	2.69
17510264	Haus8	4HAUS augmin-like complex, subunit 8	2.69
17521469			2.69
17424515	Fancg	Fanconi anemia, complementation group G	2.68
17413213	Gm26643	predicted gene, 26643 [Source:MGI Symbol;Acc:MGI:5477137]	2.68
17452038	Dtx1	deltex 1 homolog (Drosophila)	2.67
17517576	Hmg20a	high mobility group 20A	2.66
17497953	Polr2l	polymerase (RNA) II (DNA directed) polypeptide L	2.66
17266830	Nle1	notchless homolog 1 (Drosophila)	2.66
17390424	Lcmt2	leucine carboxyl methyltransferase 2	2.65
17493824	Relt	RELT tumor necrosis factor receptor	2.65
17383798	Fibcd1	fibrinogen C domain containing 1	2.65
17300839	Mphosph8	M-phase phosphoprotein 8	2.64
17349807	Pcdhac1; Pcdhac2; Gm37013; Gm38666; Pcdha4; Pcdha6; Pcdha7; Pcdha5; Pcdha11; Pcdha10; Pcdhgb1; Pcdhgb2; Pcdhgb4; Pcdhgb5; Pcdhgb6; Pcdhgb7; Pcdhgb8; Pcdhgc3; Pcdhgc4; Pcdhgc5; Pcdhga1; Pcdhga2; Pcdhga3; Pcdhga4; Pcdhga5; Pcdhga6; Pcdhga7; Pcdhga8; Pcdhga9; Pcdhga10; Pcdhga11; Pcdhga12; Pcdha1; Pcdha9; Pcdha3; Pcdha12; Pcdha2; Pcdha8	protocadherin alpha subfamily C, 1; protocadherin alpha subfamily C, 2; predicted gene, 37013; predicted gene, 38666; protocadherin alpha 4; protocadherin alpha 6; protocadherin alpha 7; protocadherin alpha 5; protocadherin alpha 11; protocadherin alpha 10; protocadherin gamma subfamily B, 1; protocadherin gamma subfamily B, 2; protocadherin gamma subfamily B, 4; protocadherin gamma subfamily B, 5; protocadherin gamma subfamily B, 6; protocadherin gamma subfamily B, 7; protocadherin gamma subfamily B, 8; protocadherin gamma subfamily C, 3; protocadherin gamma subfamily C, 4; protocadherin gamma subfamily C, 5; protocadherin gamma subfamily A, 1; protocadherin gamma subfamily A, 2; protocadherin gamma subfamily A, 3; protocadherin gamma subfamily A, 4; protocadherin gamma subfamily A, 5; protocadherin gamma subfamily A, 6; protocadherin gamma subfamily A, 7; protocadherin gamma subfamily A, 8; protocadherin gamma subfamily A, 9; protocadherin gamma subfamily A, 10; protocadherin gamma subfamily A, 11; protocadherin gamma subfamily A, 12; protocadherin alpha 1; protocadherin alpha 9; protocadherin alpha 3; protocadherin alpha 12; protocadherin alpha 2; protocadherin alpha 8	2.64
17433023	Cort	cortistatin	2.64
17308280	Ppp3cc	protein phosphatase 3, catalytic subunit, gamma isoform	2.64
17364828	Crtac1	cartilage acidic protein 1	2.63
17448880	Lnx1	ligand of numb-protein X 1	2.63
17292562	Sema4d	sema domain, immunoglobulin domain (Ig), transmembrane domain (TM) and short cytoplasmic domain, (semaphorin) 4D	2.62
17342042	Nubp2	nucleotide binding protein 2	2.62
17279427	Btbd6	BTB (POZ) domain containing 6	2.62
17548394	Gm1043	predicted gene 1043	2.62
17471252	Tspan9	tetraspanin 9	2.62
17498872	Rpl21-ps14	ribosomal protein L21, pseudogene 14 [Source:MGI Symbol;Acc:MGI:3648110]	2.61
17264363	Usp43	ubiquitin specific peptidase 43	2.6

17296836	Gm10410; Gm3047; Gm8159; Gm3239	predicted gene 10410; predicted gene 3047; predicted gene 8159; predicted gene 3239	2.6
17405819	B3galnt1	UDP-GalNAc:betaGlcNAc beta 1,3-galactosaminyltransferase, polypeptide 1	2.59
17217566	Ptpn7	protein tyrosine phosphatase, non-receptor type 7	2.58
17355058	Gm24504	predicted gene, 24504 [Source:MGI Symbol;Acc:MGI:5454281]	2.57
17240529	Foxo3; Gm38485	forkhead box O3; predicted gene, 38485	2.57
17317015	Ext1	exostoses (multiple) 1	2.56
17409594	Rnpc3	RNA-binding region (RNP1, RRM) containing 3	2.56
17248666	Rnf145	ring finger protein 145	2.56
17247675	Fam161a	family with sequence similarity 161, member A	2.56
17455748	Gm8579	spermatogenesis associated glutamate (E)-rich protein 4a pseudogene	2.55
17343628	Angptl4	angiopoietin-like 4	2.55
17386477			2.55
17288440	Mir466f-4	microRNA 466f-4	2.55
17213894	Cps1	carbamoyl-phosphate synthetase 1	2.54
17221122	Sntg1	syntrophin, gamma 1	2.54
17293988	Zfp58	zinc finger protein 58	2.54
17398634	Sh3d19	SH3 domain protein D19	2.54
17254089	Zfp830	zinc finger protein 830	2.53
17397497	Foxo1	forkhead box O1	2.52
17253937	Rhbdl3	rhomboid, veinlet-like 3 (Drosophila)	2.52
17442173	A930024E05Rik	RIKEN cDNA A930024E05 gene	2.52
17465740	3110062M04Rik	RIKEN cDNA 3110062M04 gene	2.52
17548090			2.52
17302675	Gpr180	G protein-coupled receptor 180	2.52
17407850	Ecm1	extracellular matrix protein 1	2.52
17448577	Gabra2	gamma-aminobutyric acid (GABA) A receptor, subunit alpha 2	2.51
17548197			2.5
17299849	Trav12d-2; Trav12n-2	T cell receptor alpha variable 12D-2; T cell receptor alpha variable 12N-2	2.5
17299926	Trav12d-2; Trav12n-2	T cell receptor alpha variable 12D-2; T cell receptor alpha variable 12N-2	2.5
17429840	1700021L23Rik; 4933435F18Rik	RIKEN cDNA 1700021L23 gene; RIKEN cDNA 4933435F18 gene	2.5
17514541	Mmp10	matrix metallopeptidase 10	2.5
17260855	Cep68	centrosomal protein 68	2.5
17394718	Ube2v1	ubiquitin-conjugating enzyme E2 variant 1	2.5
17230823	Lyplal1	lysophospholipase-like 1	2.5
17497633	Olfr522	olfactory receptor 522	2.49
17268445	Tbx21	T-box 21	2.49
17545904			2.48
17503862	Capns2	calpain, small subunit 2	2.48
17501905	Gdf1; Cers1	growth differentiation factor 1; ceramide synthase 1	2.48
17224395	Mir375	microRNA 375	2.47
17481770	Adm	adrenomedullin	2.47
17531693	Rtp3	receptor transporter protein 3	2.47
17445303	Mterf1a	mitochondrial transcription termination factor 1a	2.46
17503810	Irx5	Iroquois related homeobox 5 (Drosophila)	2.46
17389962	Rpap1	RNA polymerase II associated protein 1	2.46
17347267	Fez2	fasciculation and elongation protein zeta 2 (zygin II)	2.46
17481673	Rpl27a	ribosomal protein L27A	2.46
17214578	Tmem198	transmembrane protein 198	2.45

17452018	Rita1; Gm38700; Gm38465	RBPJ interacting and tubulin associated 1; predicted gene, 38700; predicted gene, 38465	2.45
17292541	9430083A17Rik	RIKEN cDNA 9430083A17 gene	2.45
17445543	Crot	carnitine O-octanoyltransferase	2.45
17442701	Fam101a	family with sequence similarity 101, member A	2.45
17301108	Arl11	ADP-ribosylation factor-like 11	2.44
17426230	Gm11209	predicted gene 11209 [Source:MGI Symbol;Acc:MGI:3650632]	2.44
17369060	Set	SET nuclear oncogene	2.44
17320240	1810021B22Rik	RIKEN cDNA 1810021B22 gene	2.43
17458885	Ppp1r17	protein phosphatase 1, regulatory subunit 17	2.43
17341044	Tcte2	t-complex-associated testis expressed 2	2.43
17545051	Acsl4	acyl-CoA synthetase long-chain family member 4	2.43
17504632	Ces4a	carboxylesterase 4A	2.43
17285157	Heatr1	HEAT repeat containing 1	2.43
17304740	Sh3bp5	SH3-domain binding protein 5 (BTK-associated)	2.43
17523161	Slc25a38	solute carrier family 25, member 38	2.42
17521171	Tlr9	toll-like receptor 9	2.42
17284280	Nudt14	nudix (nucleoside diphosphate linked moiety X)-type motif 14	2.41
17219997	Kif26b	kinesin family member 26B	2.41
17296705	Gm3095; Gm3029	predicted gene 3095; predicted gene 3029	2.41
17510351	Tmem221	transmembrane protein 221	2.4
17241137	Unc5b	unc-5 homolog B (C. elegans)	2.4
17222566	Gm23722	predicted gene, 23722 [Source:MGI Symbol;Acc:MGI:5453499]	2.4
17311437	2310069G16Rik	RIKEN cDNA 2310069G16 gene	2.4
17532710	Gm14347	predicted gene 14347	2.4
17237701	Wif1	Wnt inhibitory factor 1	2.39
17227105	Ppfia4	protein tyrosine phosphatase, receptor type, f polypeptide (PTPRF), interacting protein (liprin), alpha 4	2.39
17424057	Smu1	smu-1 suppressor of mec-8 and unc-52 homolog (C. elegans)	2.39
17363828	Ermp1	endoplasmic reticulum metallopeptidase 1	2.38
17369974	Tor2a	torsin family 2, member A	2.38
17218233	Colgalt2	collagen beta(1-O)galactosyltransferase 2	2.38
17341595	Kremen2	kringle containing transmembrane protein 2	2.38
17260440	Snora5c; Tbrg4	small nucleolar RNA, H/ACA box 5C; transforming growth factor beta regulated gene 4	2.38
17412879	Gm12404	predicted gene 12404	2.37
17235211	Midn	midnolin	2.37
17231229	Hsd11b1	hydroxysteroid 11-beta dehydrogenase 1	2.36
17341712	Amdhd2	amidohydrolase domain containing 2	2.36
17437803	N4bp2	NEDD4 binding protein 2	2.36
17484353	Lrrc27	leucine rich repeat containing 27	2.36
17374765	Spint1	serine protease inhibitor, Kunitz type 1	2.36
17332966	Gm1604b	predicted gene 1604b	2.35
17445162	Brca2	breast cancer 2, early onset	2.35
17265096	Slc2a4	solute carrier family 2 (facilitated glucose transporter), member 4	2.35
17398565	Gm6525	ribosomal protein L36a pseudogene	2.34
17501314	Gm15882	predicted gene 15882 [Source:MGI Symbol;Acc:MGI:3801770]	2.34
17444309	Grid2ip	glutamate receptor, ionotropic, delta 2 (Grid2) interacting protein 1	2.33
17378035	Bpifb6	BPI fold containing family B, member 6	2.33
17483733	Bag3	BCL2-associated athanogene 3	2.33

17364150	Pank1	pantothenate kinase 1	2.32
17349894	Pcdhb9	protocadherin beta 9	2.32
17337895	Aars2	alanyl-tRNA synthetase 2, mitochondrial (putative)	2.32
17503705	Gm3134	predicted gene 3134	2.32
17371964	Sp3os	trans-acting transcription factor 3, opposite strand	2.31
17526633	Gm10680	predicted gene 10680 [Source:MGI Symbol;Acc:MGI:3704335]	2.31
17499436	Cln8	ceroid-lipofuscinosis, neuronal 8	2.31
17487161	Qpctl	glutaminyl-peptide cyclotransferase-like	2.31
17280721	4921508M14Rik	RIKEN cDNA 4921508M14 gene [Source:MGI Symbol;Acc:MGI:1918094]	2.3
17526978	Mir34c	microRNA 34c	2.3
17516434	D630033O11Rik	RIKEN cDNA D630033O11 gene	2.3
17295649	Gtf2h2	general transcription factor II H, polypeptide 2	2.29
17527678	Pml	promyelocytic leukemia	2.29
17383723	Tor1a	torsin family 1, member A (torsin A)	2.29
17439922	Lrrc8d; D830014E11Rik	leucine rich repeat containing 8D; RIKEN cDNA D830014E11 gene	2.29
17549204			2.28
17426251	Kif12	kinesin family member 12	2.27
17387742	Olfr259	olfactory receptor 259	2.27
17536997	Zcchc13	zinc finger, CCHC domain containing 13	2.27
17537878	Bex4	brain expressed gene 4	2.27
17233032	1700027J07Rik	RIKEN cDNA 1700027J07 gene	2.27
17341758	Ntn3	netrin 3	2.27
17222072	Ccdc115	coiled-coil domain containing 115	2.26
17318140			2.26
17501855	Sugp2; Gm38427	SURP and G patch domain containing 2; predicted gene, 38427	2.25
17528741			2.25
17537592	Srpx2	sushi-repeat-containing protein, X-linked 2	2.25
17243198	Pias4	protein inhibitor of activated STAT 4	2.25
17517752	Ulk3	unc-51-like kinase 3	2.24
17239136	Gm25410	predicted gene, 25410 [Source:MGI Symbol;Acc:MGI:5455187]	2.24
17497729	Sigirr	single immunoglobulin and toll-interleukin 1 receptor (TIR) domain	2.24
17520607	4921534H16Rik	RIKEN cDNA 4921534H16 gene	2.24
17499739	Defa22	defensin, alpha, 22	2.24
17372984	Nup160	nucleoporin 160	2.24
17390614	Mfap1b	microfibrillar-associated protein 1B	2.24
17333686	Lix1	limb expression 1 homolog (chicken)	2.23
17308796	Gm1587	predicted gene 1587	2.23
17234744	1700009J07Rik	RIKEN cDNA 1700009J07 gene	2.23
17302955	1700024B18Rik	RIKEN cDNA 1700024B18 gene	2.23
17440633	Mn1	meningioma 1	2.23
17403806	Erich3	glutamate rich 3	2.23
17290121	Paip1	polyadenylate binding protein-interacting protein 1	2.23
17313852	AU022754	expressed sequence AU022754	2.22
17295005	Bhmt	betaine-homocysteine methyltransferase	2.22
17284288	Gm26583	predicted gene, 26583	2.22
17359816	Kazald1	Kazal-type serine peptidase inhibitor domain 1	2.22
17442203	Setd1b; Gm38488	SET domain containing 1B; predicted gene, 38488	2.22
17481634	Olfr512	olfactory receptor 512	2.21
17339840	Gpatch11	G patch domain containing 11	2.21
17258771	Gm11733	predicted gene 11733	2.21
17346666	4930405O22Rik	RIKEN cDNA 4930405O22 gene	2.21

17398428	Gm22531	predicted gene, 22531 [Source:MGI Symbol;Acc:MGI:5452308]	2.21
17288548	Slc12a7	solute carrier family 12, member 7	2.2
17434705	Gm8906	predicted gene 8906 [Source:MGI Symbol;Acc:MGI:3779820]	2.2
17214731	1700016L21Rik	RIKEN cDNA 1700016L21 gene	2.2
17238549	Pym1	PYM homolog 1, exon junction complex associated factor	2.2
17384202	9430024E24Rik	RIKEN cDNA 9430024E24 gene	2.2
17215879	Aqp12	aquaporin 12	2.2
17391909	5330413P13Rik	RIKEN cDNA 5330413P13 gene	2.19
17410127	Gm15551	predicted gene 15551	2.19
17430037	Tekt2	tektin 2	2.19
17439842	Pkd2	polycystic kidney disease 2	2.19
17322026	Krt2	keratin 2	2.19
17439021	Ereg	epiregulin	2.19
17258107	Cd300a	CD300A antigen	2.19
17271842	Fads6	fatty acid desaturase domain family, member 6	2.19
17260236	Myl7	myosin, light polypeptide 7, regulatory	2.18
17397120	Bbs12	Bardet-Biedl syndrome 12 (human)	2.18
17476752	Rhpn2	rhophilin, Rho GTPase binding protein 2	2.18
17328124	Litaf; Gm9861	LPS-induced TN factor; predicted gene 9861	2.17
17386396	Slc25a12	solute carrier family 25 (mitochondrial carrier, Aralar), member 12	2.17
17422565	Tmem52	transmembrane protein 52	2.17
17515869	Gm23702	predicted gene, 23702 [Source:MGI Symbol;Acc:MGI:5453479]	2.16
17509985	Armc6	armadillo repeat containing 6	2.16
17464704	Dlx6os2	distal-less homeobox 6, opposite strand 2	2.16
17265062	2810408A11Rik	RIKEN cDNA 2810408A11 gene	2.16
17469412			2.16
17408960	Cd53	CD53 antigen	2.16
17212888	Mob4	MOB family member 4, phocein	2.15
17252635	Shpk	sedoheptulokinase	2.15
17549576			2.15
17526809	Plet1os	placenta expressed transcript 1, opposite strand	2.15
17255511	Ttll6	tubulin tyrosine ligase-like family, member 6	2.15
17527206	Gldnos	gliomedin, opposite strand	2.15
17506356	Zfpm1	zinc finger protein, multitype 1	2.15
17430521	Tmem39b	transmembrane protein 39b	2.14
17350790	Prrc1	proline-rich coiled-coil 1	2.14
17433942	Gm25982	predicted gene, 25982 [Source:MGI Symbol;Acc:MGI:5455759]	2.14
17235243	Efna2	ephrin A2	2.14
17463169	Scnn1a	sodium channel, nonvoltage-gated 1 alpha	2.14
17429331	4930538K18Rik; AU022252	RIKEN cDNA 4930538K18 gene; expressed sequence AU022252	2.14
17355231	Ptpn2	protein tyrosine phosphatase, non-receptor type 2	2.14
17440361	Plcxd1	phosphatidylinositol-specific phospholipase C, X domain containing 1	2.14
17230457	6330403A02Rik	RIKEN cDNA 6330403A02 gene	2.14
17509801	Lzts1	leucine zipper, putative tumor suppressor 1	2.13
17344626	C920025E04Rik	RIKEN cDNA C920025E04 gene	2.13
17232763	Ddo	D-aspartate oxidase	2.13
17538452	Gm15080	predicted gene 15080	2.13
17376883	Gm14061	predicted gene 14061	2.13
17387210	Cwc22	CWC22 spliceosome-associated protein homolog (S. cerevisiae)	2.13

17285527	Tcrg-V3	T cell receptor gamma, variable 3	2.13
17323816	Gm10088	predicted gene 10088 [Source:MGI Symbol;Acc:MGI:3641695]	2.12
17490218	1700008O03Rik	RIKEN cDNA 1700008O03 gene	2.12
17524590	Zglp1	zinc finger, GATA-like protein 1	2.12
17314562			2.12
17365718	Gm16299	predicted gene 16299	2.11
17538431	Mir1912	microRNA 1912	2.11
17254547	Bcas3	breast carcinoma amplified sequence 3	2.11
17501919	Comp	cartilage oligomeric matrix protein	2.11
17392317	Pcsk2os2	proprotein convertase subtilisin/kexin type 2, opposite strand 2	2.11
17274310	Hpcal1	hippocalcin-like 1	2.11
17414327	Actl7a	actin-like 7a	2.11
17474676	Gm16175	predicted gene 16175 [Source:MGI Symbol;Acc:MGI:3801805]	2.1
17337497	Zfp57	zinc finger protein 57	2.1
17368359	Snhg7os	small nucleolar RNA host gene 7, opposite strand	2.1
17274963	Gm24429	predicted gene, 24429 [Source:MGI Symbol;Acc:MGI:5454206]	2.09
17515703	Adamts8	a disintegrin-like and metallopeptidase (reprolysin type) with thrombospondin type 1 motif, 8	2.09
17414385	Musk	muscle, skeletal, receptor tyrosine kinase	2.09
17407522	Gm10697; Tdpoz5; Gm9107	predicted gene 10697; TD and POZ domain containing 5; predicted gene 9107	2.09
17407529	Gm10697; Tdpoz5	predicted gene 10697; TD and POZ domain containing 5	2.09
17525964	Gm26272	predicted gene, 26272 [Source:MGI Symbol;Acc:MGI:5456049]	2.09
17409994			2.09
17476311	Thap8	THAP domain containing 8 [Source:MGI Symbol;Acc:MGI:1922879]	2.08
17405057	1700027H10Rik	RIKEN cDNA 1700027H10 gene	2.08
17220866	Rd3	retinal degeneration 3	2.08
17501270	Gm25992	predicted gene, 25992 [Source:MGI Symbol;Acc:MGI:5455769]	2.08
17394582	Prex1	phosphatidylinositol-3,4,5-trisphosphate-dependent Rac exchange factor 1	2.08
17444389	D130017N08Rik	RIKEN cDNA D130017N08 gene	2.08
17327602	1700037C18Rik	RIKEN cDNA 1700037C18 gene	2.07
17403255	Gbp2b	guanylate binding protein 2b	2.07
17342308	Chtf18	CTF18, chromosome transmission fidelity factor 18	2.07
17289656	Htr1a	5-hydroxytryptamine (serotonin) receptor 1A	2.07
17319029	Sstr3	somatostatin receptor 3	2.07
17311128	Gm16137	predicted gene 16137 [Source:MGI Symbol;Acc:MGI:3802136]	2.07
17276622	Fntb	farnesyltransferase, CAAX box, beta	2.07
17243644	Cry1	cryptochrome 1 (photolyase-like)	2.07
17240045	Hint3	histidine triad nucleotide binding protein 3	2.07
17380631	4930591A17Rik	RIKEN cDNA 4930591A17 gene	2.06
17417107	Foxd2os	forkhead box D2, opposite strand	2.06
17394012	Gm14254	predicted gene 14254 [Source:MGI Symbol;Acc:MGI:3650986]	2.06
17380969	Rtel1	regulator of telomere elongation helicase 1	2.06
17514653	Gm16485	predicted gene 16485 [Source:MGI Symbol;Acc:MGI:3642771]	2.06
17459449	Foxi3	forkhead box I3	2.06

17493378	Gm25860	predicted gene, 25860 [Source:MGI Symbol;Acc:MGI:5455637]	2.05
17267443	Gdpd1	glycerophosphodiester phosphodiesterase domain containing 1	2.05
17322642	Ubn1	ubinuclein 1	2.05
17292828	Mxd3	Max dimerization protein 3	2.04
17513281	Gm16118	predicted gene 16118	2.04
17337945	Gm17080	predicted gene 17080 [Source:MGI Symbol;Acc:MGI:4937907]	2.04
17533755	Slc6a14	solute carrier family 6 (neurotransmitter transporter), member 14	2.04
17440134	Gm26387	predicted gene, 26387 [Source:MGI Symbol;Acc:MGI:5456164]	2.04
17430231	Gm12947	predicted gene 12947 [Source:MGI Symbol;Acc:MGI:3649527]	2.04
17282158	Rdh11	retinol dehydrogenase 11	2.04
17380119	Cass4	Cas scaffolding protein family member 4	2.04
17267329	Vmp1; Mir21a	vacuole membrane protein 1; microRNA 21a	2.04
17471990	Gm17089	predicted gene 17089	2.04
17247225	Upp1	uridine phosphorylase 1	2.04
17515103	1700084C06Rik	RIKEN cDNA 1700084C06 gene [Source:MGI Symbol;Acc:MGI:1923868]	2.04
17456204	Capza2	capping protein (actin filament) muscle Z-line, alpha 2	2.04
17289031	Rps23	ribosomal protein S23	2.04
17416481	Ndc1	NDC1 transmembrane nucleoporin	2.03
17305198	Sh2d4b	SH2 domain containing 4B	2.03
17306906	Ripk3	receptor-interacting serine-threonine kinase 3	2.03
17506353	Gm22	predicted gene 22	2.03
17418057	Gm24678	predicted gene, 24678 [Source:MGI Symbol;Acc:MGI:5454455]	2.03
17515478	Bmper	BMP-binding endothelial regulator	2.03
17354356	Hdhd1a	haloacid dehalogenase-like hydrolase domain containing 1A	2.02
17316754	Lrp12	low density lipoprotein-related protein 12	2.02
17504207	Adgrg3	adhesion G protein-coupled receptor G3	2.02
17549536			2.02
17549700			2.02
17378387	LOC102636309; Gssos2; Gssos1	uncharacterized LOC102636309; glutathione synthase, opposite strand 2 [Source:MGI Symbol;Acc:MGI:3702171]; glutathione synthase, opposite strand 1 [Source:MGI Symbol;Acc:MGI:1915623]	2.02
17269785	Plekhh3	pleckstrin homology domain containing, family H (with MyTH4 domain) member 3	2.02
17327524	Igsf5	immunoglobulin superfamily, member 5	2.02
17314699	Prph	peripherin	2.02
17547569			2.02
17332932	Sytl3	synaptotagmin-like 3	2.02
17301502	Adra1a	adrenergic receptor, alpha 1a	2.02
17309287	Pou4f1	POU domain, class 4, transcription factor 1	2.02
17298364	Nt5dc2	5-nucleotidase domain containing 2	2.02
17353271	Myo7b	myosin VIIB	2.01
17257599	Milr1	mast cell immunoglobulin like receptor 1	2.01
17441924	Tmem116	transmembrane protein 116	2.01
17241840	Lrrc75b	leucine rich repeat containing 75B	2.01
17339923	Ttc39d	tetratricopeptide repeat domain 39D	2.01
17494822	Nlrp10	NLR family, pyrin domain containing 10	2.01
17338231	Gm26216	predicted gene, 26216 [Source:MGI Symbol;Acc:MGI:5455993]	-15.55

17541917	Snord61	small nucleolar RNA, C/D box 61	-27.1

Table S5: Differentially expressed genes in western diet (WD) fed LDL-R -/- mice when compared to control diet (CD) fed WT mice.

Transcript Cluster ID	Gene Symbol	Description	Fold Change
17320842	Gm24668	predicted gene, 24668 [Source:MGI Symbol;Acc:MGI:5454445]	167.27
17350916	Gm4951	predicted gene 4951	142.6
17348840	Rnf125	ring finger protein 125	117.1
17278822	Mir679	microRNA 679	113.69
17285225	LOC105245453	nidogen-1-like; predicted gene 2399 [Source:MGI Symbol;Acc:MGI:3780567]	112.35
17285225	Gm2399	nidogen-1-like; predicted gene 2399 [Source:MGI Symbol;Acc:MGI:3780567]	112.35
17472192	Mgp	matrix Gla protein	109.3
17345664			96.35
17308731			85.21
17245302	Rap1b	RAS related protein 1b	84.68
17312829	Lgals1	lectin, galactose binding, soluble 1	81.38
17278688	Mir673	microRNA 673	81.06
17423289	Gm22940	predicted gene, 22940 [Source:MGI Symbol;Acc:MGI:5452717]	75.5
17385902	Slc38a11	solute carrier family 38, member 11	72.21
17324110	Snord66	small nucleolar RNA, C/D box 66	70.95
17245700	Tspan31	tetraspanin 31	70.47
17317708	Mir30d	microRNA 30d	69.19
17401999	Mir669n	microRNA 669n	67.85
17318403	Gm25720	predicted gene, 25720 [Source:MGI Symbol;Acc:MGI:5455497]	67.3
17384173	Gm23546	predicted gene, 23546 [Source:MGI Symbol;Acc:MGI:5453323]	66.56
17446058	Gm24009	predicted gene, 24009 [Source:MGI Symbol;Acc:MGI:5453786]	65.3
17383129	Snora17	small nucleolar RNA, H/ACA box 17; small nucleolar RNA host gene 7	64.98
17383129	Snhg7	small nucleolar RNA, H/ACA box 17; small nucleolar RNA host gene 7	64.98
17355437	Gm23119	predicted gene, 23119 [Source:MGI Symbol;Acc:MGI:5452896]	63.09
17494221	Hbb-bt	hemoglobin, beta adult t chain; hemoglobin, beta adult minor chain	62.45
17494221	Hbb-b2	hemoglobin, beta adult t chain; hemoglobin, beta adult minor chain	62.45
17400549	Gm20634	predicted gene 20634 [Source:MGI Symbol;Acc:MGI:5313081]	55.73
17348602	Gm36490	predicted gene, 36490	55.42
17476641	Gm25817	predicted gene, 25817 [Source:MGI Symbol;Acc:MGI:5455594]; predicted gene, 24766 [Source:MGI Symbol;Acc:MGI:5454543]	52.55
17476646	Gm24766	predicted gene, 25817 [Source:MGI Symbol;Acc:MGI:5455594]; predicted gene, 24766 [Source:MGI Symbol;Acc:MGI:5454543]	52.55
17532509	Slc6a20a	solute carrier family 6 (neurotransmitter transporter), member 20A	51.93
17287022	Gm24915	predicted gene, 24915 [Source:MGI Symbol;Acc:MGI:5454692]	50.66
17238882	Syne1	spectrin repeat containing, nuclear envelope 1	49.88
17238860	Syne1	spectrin repeat containing, nuclear envelope 1	48.94
17480568	Arrb1	arrestin, beta 1	47.16
17408021	Hist2h4	histone cluster 2, H4	44.97
17547859	Gm2573	predicted gene 2573	42.31
17233294	Nus1	nuclear undecaprenyl pyrophosphate synthase 1 homolog (S. cerevisiae)	40.45
17340599	Dynlt1f	dynein light chain Tctex-type 1F	40.33
17291355	Acot13	acyl-CoA thioesterase 13	39
17548983			36.88
17328895	Gm25777	predicted gene, 25777 [Source:MGI Symbol;Acc:MGI:5455554]	36.23
17378896			35.71
17512752	LOC102642963	40S ribosomal protein S26-like; ribosomal protein S26, pseudogene 1 [Source:MGI Symbol;Acc:MGI:3704322]	34.97
17512752	Rps26-ps1	40S ribosomal protein S26-like; ribosomal protein S26, pseudogene 1 [Source:MGI Symbol;Acc:MGI:3704322]	34.97
17329079	Parl	presenilin associated, rhomboid-like	33.99
17365070	Gm24336	predicted gene, 24336 [Source:MGI Symbol;Acc:MGI:5454113]	33.24
17238908	Syne1	spectrin repeat containing, nuclear envelope 1	31.35
17287891			29.93
17418211	Gm25788	predicted gene, 25788 [Source:MGI Symbol;Acc:MGI:5455565]	29.72
17458514	Npy	neuropeptide Y	29.5
17273562	Gm12590	predicted gene 12590 [Source:MGI Symbol;Acc:MGI:3650370]	28.43
17430140	Ncdn	neurochondrin	28.14
17238884	Syne1	spectrin repeat containing, nuclear envelope 1	28.05
17254508	Car4	carbonic anhydrase 4	27.29

17363107	Zfp91	zinc finger protein 91; ciliary neurotrophic factor; Zfp91-Cntf readthrough transcript (NMD candidate)	27.1
17363107	Cntf	zinc finger protein 91; ciliary neurotrophic factor; Zfp91-Cntf readthrough transcript (NMD candidate)	27.1
17363107	Zfp91Cntf	zinc finger protein 91; ciliary neurotrophic factor; Zfp91-Cntf readthrough transcript (NMD candidate)	27.1
17236800	Dcn	decorin	26.62
17483190	Kctd13	potassium channel tetramerisation domain containing 13	25.72
17250365	Gid4	GID complex subunit 4, VID24 homolog (S. cerevisiae)	24.87
17303433	Fam107a	family with sequence similarity 107, member A	24.58
17547507			24.53
17306856	Dhrs1	dehydrogenase/reductase (SDR family) member 1	24.18
17336896	Clic1	chloride intracellular channel 1	23.97
17547931			23.85
17542392	Idh3g	isocitrate dehydrogenase 3 (NAD+), gamma	23.71
17382361	Gm24328	predicted gene, 24328 [Source:MGI Symbol;Acc:MGI:5454105]	23.68
17347953	Fbxo11	F-box protein 11	23.63
17289787	Rps3a3	ribosomal protein S3A3; ribosomal protein S3A1	22.78
17289787	Rps3a1	ribosomal protein S3A3; ribosomal protein S3A1	22.78
17282104	Atp6v1d	ATPase, H+ transporting, lysosomal V1 subunit D	22.75
17549910			22.72
17266038	Fam101b	family with sequence similarity 101, member B	22.29
17335168	Snrpc	U1 small nuclear ribonucleoprotein C	22.09
17488951	Zfp146	zinc finger protein 146	21.94
17213189	Gm20257	caspase 8 pseudogene	21.53
17340629	Mir692-1	microRNA 692-1	21.43
17308999	Rps3a2	ribosomal protein S3A2	20.98
17338872	Ndufa11	NADH dehydrogenase (ubiquinone) 1 alpha subcomplex 11	20.91
17392718	Platr15	pluripotency associated transcript 15 [Source:MGI Symbol;Acc:MGI:3650105]	20.31
17340522	Gm15590	predicted gene 15590 [Source:MGI Symbol;Acc:MGI:3831433]; predicted gene, 22774 [Source:MGI Symbol;Acc:MGI:5452551]	20.29
17340522	Gm22774	predicted gene 15590 [Source:MGI Symbol;Acc:MGI:3831433]; predicted gene, 22774 [Source:MGI Symbol;Acc:MGI:5452551]	20.29
17418483	Mir692-3	microRNA 692-3; microRNA 692-2; ferritin light polypeptide 1; ferritin light polypeptide 2, pseudogene	20.29
17418483	Mir692-2	microRNA 692-3; microRNA 692-2; ferritin light polypeptide 1; ferritin light polypeptide 2, pseudogene	20.29
17418483	Ftl1	microRNA 692-3; microRNA 692-2; ferritin light polypeptide 1; ferritin light polypeptide 2, pseudogene	20.29
17418483	Ftl2-ps	microRNA 692-3; microRNA 692-2; ferritin light polypeptide 1; ferritin light polypeptide 2, pseudogene	20.29
17369948	Mir1954	microRNA 1954	20.12
17340197	Epas1	endothelial PAS domain protein 1	20.12
17327757	Pam16	presequence translocase-asssociated motor 16 homolog (S. cerevisiae)	19.91
17468520	Snrnp27	small nuclear ribonucleoprotein 27 (U4/U6.U5)	19.79
17391373	1500011K16Rik	RIKEN cDNA 1500011K16 gene	19.57
17505673	St3gal2	ST3 beta-galactoside alpha-2,3-sialyltransferase 2	19.43
17281748	Trim9	tripartite motif-containing 9	18.95
17522430	Ccdc12	coiled-coil domain containing 12	18.92
17216777	Gm23734	predicted gene, 23734 [Source:MGI Symbol;Acc:MGI:5453511]	18.87
17495667	Thumpd1	THUMP domain containing 1	18.79
17289551	Gm16416	predicted gene 16416 [Source:MGI Symbol;Acc:MGI:3646635]	18.55
17548131	Gm10862	predicted gene 10862 [Source:MGI Symbol;Acc:MGI:3641622]	18.49
17548190			18.01
17548525	Hnrnpf	heterogeneous nuclear ribonucleoprotein F	17.99
17237978	Ddit3	DNA-damage inducible transcript 3	17.89
17325719	BC002163	NADH dehydrogenase Fe-S protein 5 pseudogene; NADH dehydrogenase (ubiquinone) Fe-S protein 5	17.57
17325719	Ndufs5	NADH dehydrogenase Fe-S protein 5 pseudogene; NADH dehydrogenase (ubiquinone) Fe-S protein 5	17.57
17550288			17.46
17373776	Gm22757;Gm13875	predicted gene, 22757 [Source:MGI Symbol;Acc:MGI:5452534]; predicted gene 13875 [Source:MGI Symbol;Acc:MGI:3649350]	17.11
17393764	Gm23134	predicted gene, 23134 [Source:MGI Symbol;Acc:MGI:5452911]	16.89

17437043	Cytl1	cytokine-like 1	16.61
17514122	2810004N23Rik	RIKEN cDNA 2810004N23 gene	16.61
17300381	Cmtm5	CKLF-like MARVEL transmembrane domain containing 5	16.23
17379873	1500012F01Rik	RIKEN cDNA 1500012F01 gene	16.11
17248331	Fbxw11	F-box and WD-40 domain protein 11	16.07
17548238	Fabp5	fatty acid binding protein 5, epidermal	15.99
17273292	Dcxr	dicarbonyl L-xylulose reductase	15.91
17361988	Arl2	ADP-ribosylation factor-like 2	15.91
17282905	Snw1	SNW domain containing 1	15.86
17238842	Syne1	spectrin repeat containing, nuclear envelope 1	15.83
17490791	Snrnp70	small nuclear ribonucleoprotein 70 (U1)	15.8
17499279	Lamp1	lysosomal-associated membrane protein 1	15.64
17494408	Trim30d	tripartite motif-containing 30D	15.5
17493730	Rnf169	ring finger protein 169	15.49
17311179	Fzd6	frizzled homolog 6 (Drosophila)	15.34
17547767			15.12
17216010	Gm17415	predicted gene, 17415 [Source:MGI Symbol;Acc:MGI:4937049]	15.06
17358219	Mir1192	microRNA 1192	15.06
17497718	Ifitm3	interferon induced transmembrane protein 3	15.04
17516607	Rps25	ribosomal protein S25	14.89
17532597	mt-Tv	mitochondrially encoded tRNA valine [Source:MGI Symbol;Acc:MGI:102472]	14.78
17391903			14.47
17349713	Ankhd1	ankyrin repeat and KH domain containing 1	14.44
17367438	Gm23970	predicted gene, 23970 [Source:MGI Symbol;Acc:MGI:5453747]	14.37
17245445	Gm15961	predicted gene 15961 [Source:MGI Symbol;Acc:MGI:3802142]	14.25
17300119	Trdv2-2	T cell receptor delta variable 2-2	14.18
17334819	0610011F06Rik	RIKEN cDNA 0610011F06 gene	14.05
17346311	2410015M20Rik	RIKEN cDNA 2410015M20 gene	13.54
17474983	Cadm4	cell adhesion molecule 4	13.48
17340500	Atp5g2	ATP synthase, H+ transporting, mitochondrial F0 complex, subunit C2 (subunit 9)	13.46
17475671	Ttc9b	tetratricopeptide repeat domain 9B	13.42
17288145	Habp4	hyaluronic acid binding protein 4	13.38
17402981	Emcn	endomucin	13.19
17287454	Arl10	ADP-ribosylation factor-like 10	13.15
17452456	Camkk2	calcium/calmodulin-dependent protein kinase kinase 2, beta	13.1
17320474	Sco2; Tymp	SCO cytochrome oxidase deficient homolog 2 (yeast); thymidine phosphorylase	13.09
17547933			12.92
17223277	Gm6644	Akr1b3 pseudogene	12.85
17257175	Crhr1	corticotropin releasing hormone receptor 1	12.79
17262990	Sparc	secreted acidic cysteine rich glycoprotein	12.73
17294338	Exoc3	exocyst complex component 3	12.68
17438969	Pf4	platelet factor 4	12.58
17400682	Ankrd35	ankyrin repeat domain 35	12.49
17479450	9330171B17Rik	RIKEN cDNA 9330171B17 gene [Source:MGI Symbol;Acc:MGI:3696417]	12.15
17279835	Gm17541	predicted gene, 17541 [Source:MGI Symbol;Acc:MGI:4937175]	12.08
17304512	Gm24916	predicted gene, 24916 [Source:MGI Symbol;Acc:MGI:5454693]	11.99
17388599	Ttc17	tetratricopeptide repeat domain 17	11.97
17218377	Acbd6	acyl-Coenzyme A binding domain containing 6	11.96
17227278	Rnpep	arginyl aminopeptidase (aminopeptidase B)	11.95
17516030	Tmem218	transmembrane protein 218	11.75
17514601	Gm29376	predicted gene 29376	11.73
17481863	Mical2	microtubule associated monooxygenase, calponin and LIM domain containing 2	11.71
17378596	Gm22759;Gm14251	predicted gene, 22759 [Source:MGI Symbol;Acc:MGI:5452536]; predicted gene 14251 [Source:MGI Symbol;Acc:MGI:3650992]	11.66
17339989	Rpl21-ps7	ribosomal protein L21, pseudogene 7 [Source:MGI Symbol;Acc:MGI:3646135]	11.65
17269246	Krtap4-13	keratin associated protein 4-13	11.59
17515248	Carm1	coactivator-associated arginine methyltransferase 1	11.55
17269638	Rab5c	RAB5C, member RAS oncogene family	11.51
17472983	Gm23456	predicted gene, 23456 [Source:MGI Symbol;Acc:MGI:5453233]	11.45
17424075	Gm12396	predicted gene 12396 [Source:MGI Symbol;Acc:MGI:3649201]	11.37
17295212	Polk	polymerase (DNA directed), kappa	11.35
17242842	Gamt	guanidinoacetate methyltransferase	11.32
17497811	Mir210	microRNA 210	11.24
17494643	Gm4070	predicted gene 4070	11.22

17358225	Gm22144	predicted gene, 22144 [Source:MGI Symbol;Acc:MGI:5451921]	11.18
17324436			11.05
17311058	Gm24771	predicted gene, 24771 [Source:MGI Symbol;Acc:MGI:5454548]	11.03
17488179	Ltbp4	latent transforming growth factor beta binding protein 4	11
17450527	Rps15a-ps5	ribosomal protein S15A, pseudogene 5	10.93
17493034			10.57
17332447	Hmgn1	high mobility group nucleosomal binding domain 1	10.54
17440998	Ankrd13a	ankyrin repeat domain 13a	10.5
17436427	Maea	macrophage erythroblast attacher	10.49
17548616	Gm6415	predicted pseudogene 6415 [Source:MGI Symbol;Acc:MGI:3648526]	10.48
17360068	Wbp11	WW domain binding protein 1 like	10.42
17238862	Syne1	spectrin repeat containing, nuclear envelope 1	10.33
17415292	Gm13271	predicted gene 13271	10.33
17262836	Cdc42se2	CDC42 small effector 2	10.28
17427117	Gm16686	predicted gene, 16686 [Source:MGI Symbol;Acc:MGI:4439610]	10.26
17383199	Rexo4	REX4, RNA exonuclease 4 homolog (S. cerevisiae)	10.24
17470597	Slc2a3	solute carrier family 2 (facilitated glucose transporter), member 3	10.21
17483337	Srcap; Tmem265	Snf2-related CREBBP activator protein; transmembrane protein 265	10.1
17402604	Gm9396	predicted gene 9396 [Source:MGI Symbol;Acc:MGI:3645563]	10.09
17521681	Gpx1	glutathione peroxidase 1	10.07
17224071	Fn1	fibronectin 1	10.07
17356401	Eif1ad	eukaryotic translation initiation factor 1A domain containing	9.96
17290074	Rpl34-ps2	ribosomal protein L34, pseudogene 2 [Source:MGI Symbol;Acc:MGI:3648994]	9.91
17513603	Zcchc14	zinc finger, CCHC domain containing 14	9.88
17229451			9.79
17457876	Gstk1	glutathione S-transferase kappa 1	9.76
17301582	Nefl	neurofilament, light polypeptide	9.72
17274471	Iah1	isoamyl acetate-hydrolyzing esterase 1 homolog (S. cerevisiae)	9.58
17279169	Mark3	MAP/microtubule affinity regulating kinase 3	9.57
17257085	Hexim1	hexamethylene bis-acetamide inducible 1	9.56
17476637	Gm5116	predicted gene 5116 [Source:MGI Symbol;Acc:MGI:3649164]	9.5
17405174	Cog6	component of oligomeric golgi complex 6	9.49
17435867	Tmem214	transmembrane protein 214	9.43
17520492	Xrn1	5-3 exoribonuclease 1	9.43
17333092	Sft2d1	SFT2 domain containing 1	9.35
17463298	Gm16303	predicted gene 16303 [Source:MGI Symbol;Acc:MGI:3826584]	9.3
17429976	Thrap3	thyroid hormone receptor associated protein 3	9.29
17394292	Neurl2	neuralized E3 ubiquitin protein ligase 2	9.18
17550246			9.15
17463031	Acrbp	proacrosin binding protein	9.02
17238866	Syne1	spectrin repeat containing, nuclear envelope 1	9
17333932	Zfp53	zinc finger protein 53	8.99
17269206	Gm14180	predicted gene 14180 [Source:MGI Symbol;Acc:MGI:3650017]	8.9
17326394	Filip1l	filamin A interacting protein 1-like	8.89
17234667	Pttg1ip	pituitary tumor-transforming 1 interacting protein	8.7
17271531	Gm11677	predicted gene 11677	8.68
17374045			8.54
17494574	Gm22504	predicted gene, 22504 [Source:MGI Symbol;Acc:MGI:5452281]	8.42
17323909	Dvl3	dishevelled 3, dsh homolog (Drosophila)	8.4
17382822	Nacc2	nucleus accumbens associated 2, BEN and BTB (POZ) domain containing	8.37
17222096	Fam168b	family with sequence similarity 168, member B	8.3
17338637	Zfp959	zinc finger protein 959	8.23
17500808	Gm6180	predicted pseudogene 6180 [Source:MGI Symbol;Acc:MGI:3643972]	8.19
17378784	Rprd1b	regulation of nuclear pre-mRNA domain containing 1B	8.1
17304162	Tmem254b; Tmem254c; Tmem254a	transmembrane protein 254b; transmembrane protein 254c; transmembrane protein 254a	8.09
17279528	Tmem121	transmembrane protein 121	7.99
17355825	Cndp2	CNDP dipeptidase 2 (metallopeptidase M20 family)	7.96
17437702	Mir574	microRNA 574	7.94
17387514	Gm19426	predicted gene, 19426	7.93
17424850	Rnf38	ring finger protein 38	7.85
17231248	G0s2	G0/G1 switch gene 2	7.8
17500876	Sorbs2	sorbin and SH3 domain containing 2	7.73
17263837	Epn2	epsin 2	7.69

17526382	Kmt2a	lysine (K)-specific methyltransferase 2A	7.68
17301899	Sucla2; Gm41209	succinate-Coenzyme A ligase, ADP-forming, beta subunit; predicted gene, 41209	7.67
17540521	Cfp	complement factor properdin	7.67
17460405	Tia1	cytotoxic granule-associated RNA binding protein 1	7.59
17320457	Lmf2	lipase maturation factor 2	7.55
17495412	Snord14a; Rps13	small nucleolar RNA, C/D box 14A; ribosomal protein S13	7.54
17311796	Ndufb9	NADH dehydrogenase (ubiquinone) 1 beta subcomplex, 9	7.52
17361285	Syt12	synaptotagmin XII	7.49
17399333	Gba	glucosidase, beta, acid	7.47
17240164	Rwdd1	RWD domain containing 1	7.45
17294368	Pdcd6	programmed cell death 6	7.45
17341769	1600002H07Rik	RIKEN cDNA 1600002H07 gene	7.45
17289239	Tbca	tubulin cofactor A	7.36
17328810	Dgcr14	DiGeorge syndrome critical region gene 14	7.36
17537722	Hnrnph2	heterogeneous nuclear ribonucleoprotein H2	7.33
17356897	Rasgrp2	RAS, guanyl releasing protein 2	7.29
17449958	Mir703	microRNA 703	7.27
17535296	n-R5s8	nuclear encoded rRNA 5S 8 [Source:MGI Symbol;Acc:MGI:4421742]	7.26
17353962	Fgf1	fibroblast growth factor 1	7.24
17366338			7.15
17359344	Entpd1	ectonucleoside triphosphate diphosphohydrolase 1	7.14
17233699	Rpl27a-ps1	ribosomal protein L27A, pseudogene 1 [Source:MGI Symbol;Acc:MGI:3645354]	7.07
17406908	Fdps	farnesyl diphosphate synthetase	7.05
17327950	AU021092	expressed sequence AU021092	7
17422521	Cfap74	cilia and flagella associated protein 74	6.93
17430354	Rbbp4	retinoblastoma binding protein 4	6.93
17474143	Slc1a5	solute carrier family 1 (neutral amino acid transporter), member 5	6.92
17289249	Wdr41	WD repeat domain 41	6.9
17496397	Ppp4c	protein phosphatase 4, catalytic subunit	6.9
17237952	F420014N23Rik	RIKEN cDNA F420014N23 gene	6.86
17271763	Cd300lh	CD300 antigen like family member H	6.81
17302356	Gm24770	predicted gene, 24770 [Source:MGI Symbol;Acc:MGI:5454547]	6.8
17478883	Mcee	methylmalonyl CoA epimerase	6.8
17243247	Mrpl54	mitochondrial ribosomal protein L54	6.74
17216659	3110009E18Rik	RIKEN cDNA 3110009E18 gene	6.74
17303623			6.72
17250549	Kcnj12	potassium inwardly-rectifying channel, subfamily J, member 12	6.71
17456736	Ahcyl2	S-adenosylhomocysteine hydrolase-like 2	6.68
17405021	Sclt1	sodium channel and clathrin linker 1	6.62
17296943	Gm10410; Gm8281	predicted gene 10410; predicted pseudogene 8281	6.6
17474377	Nova2	neuro-oncological ventral antigen 2	6.56
17492066	Nr2f2	nuclear receptor subfamily 2, group F, member 2	6.56
17513995	Acta1	actin, alpha 1, skeletal muscle	6.51
17275555	Psma6	proteasome (prosome, macropain) subunit, alpha type 6	6.49
17237074	Gm4340	predicted gene 4340	6.44
17496338	Il27	interleukin 27	6.39
17214023	Gm5528	predicted gene 5528 [Source:MGI Symbol;Acc:MGI:3643453]	6.38
17388803	Cat	catalase	6.37
17373984	Immp1l	IMP1 inner mitochondrial membrane peptidase-like (S. cerevisiae)	6.36
17362111	Esrra; Mir6990	estrogen related receptor, alpha; microRNA 6990	6.36
17548806	Gm14567	predicted gene 14567 [Source:MGI Symbol;Acc:MGI:3705732]	6.33
17236739	Ube2n	ubiquitin-conjugating enzyme E2N	6.31
17233394	Smpdl3a	sphingomyelin phosphodiesterase, acid-like 3A	6.31
17221792	Fam135a	family with sequence similarity 135, member A	6.28
17283345	Smek1	SMEK homolog 1, suppressor of mek1 (Dictyostelium)	6.25
17334902	Gm23123	predicted gene, 23123 [Source:MGI Symbol;Acc:MGI:5452900]	6.21
17362689	Sdhaf2	succinate dehydrogenase complex assembly factor 2	6.17
17409953	Gm4609	predicted gene 4609 [Source:MGI Symbol;Acc:MGI:3782792]	6.16
17550412			6.15
17274389			6.14
17400630	Rbm8a	RNA binding motif protein 8a	6.12
17448021	Dhx15	DEAH (Asp-Glu-Ala-His) box polypeptide 15	6.11
17342038	Fahd1	fumarylacetoacetate hydrolase domain containing 1	6.05
17366536	Ccdc3	coiled-coil domain containing 3	6.04

17269210			6.02
17446100	Fam126a	family with sequence similarity 126, member A	6.02
17242025	Prmt2; Mir678	protein arginine N-methyltransferase 2; microRNA 678	6.01
17300251	Abhd4	abhydrolase domain containing 4	6
17341619	Flywch2	FLYWCH family member 2	5.97
17291653	Gmds	GDP-mannose 4, 6-dehydratase	5.94
17324420	St6gal1	beta galactoside alpha 2,6 sialyltransferase 1	5.94
17323138	Abcc1	ATP-binding cassette, sub-family C (CFTR/MRP), member 1	5.92
17235597	Atcayos	ataxia, cerebellar, Cayman type homolog (human), opposite strand	5.91
17449647	Gm15710	predicted gene 15710 [Source:MGI Symbol;Acc:MGI:3783151]	5.86
17309099	Klf12	Kruppel-like factor 12	5.77
17467831	Tmsb10	thymosin, beta 10	5.75
17474371	Pglyrp1	peptidoglycan recognition protein 1	5.71
17366932	Mir466h	microRNA 466h	5.7
17224270	Tmbim1	transmembrane BAX inhibitor motif containing 1	5.7
17284645	Ighv1-75	immunoglobulin heavy variable 1-75	5.68
17470029	Gm23527	predicted gene, 23527 [Source:MGI Symbol;Acc:MGI:5453304]	5.66
17235511	Gadd45b	growth arrest and DNA-damage-inducible 45 beta	5.6
17314164	Mapk8ip2	mitogen-activated protein kinase 8 interacting protein 2	5.58
17319588	Tnfrsf13c	tumor necrosis factor receptor superfamily, member 13c	5.57
17335060	Itpr3	inositol 1,4,5-triphosphate receptor 3	5.54
17479099	Igf1r	insulin-like growth factor I receptor	5.53
17392414	Dzank1	double zinc ribbon and ankyrin repeat domains 1	5.51
17387912	Olfr1226	olfactory receptor 1226	5.45
17353169	Gm9955	predicted gene 9955 [Source:MGI Symbol;Acc:MGI:3642397]	5.44
17361344	Gm21992; Rbm14	predicted gene 21992; RNA binding motif protein 14	5.43
17431049	Sfn	stratifin	5.42
17399044	Smg5	Smg-5 homolog, nonsense mediated mRNA decay factor (C. elegans)	5.4
17313322	L3mbtl2	l(3)mbt-like 2 (Drosophila)	5.38
17328870	Zdhhc8	zinc finger, DHHC domain containing 8	5.37
17515062	Mrpl4	mitochondrial ribosomal protein L4	5.36
17541493	Enox2	ecto-NOX disulfide-thiol exchanger 2	5.29
17266074	Gemin4; Glod4	gem (nuclear organelle) associated protein 4; glyoxalase domain containing 4	5.27
17279737	Rab10	RAB10, member RAS oncogene family	5.24
17527421	Tspan3	tetraspanin 3	5.24
17443430	Polr2j	polymerase (RNA) II (DNA directed) polypeptide J	5.22
17338571	Kat2b	K(lysine) acetyltransferase 2B	5.21
17218765	Kifap3	kinesin-associated protein 3	5.21
17246790	Gatsl3	GATS protein-like 3	5.2
17361683	Sipa1	signal-induced proliferation associated gene 1	5.2
17289031	Rps23	ribosomal protein S23	5.17
17550192			5.16
17286808	Gm10790	predicted gene 10790	5.16
17504861	Ctcf	CCCTC-binding factor	5.16
17308280	Ppp3cc	protein phosphatase 3, catalytic subunit, gamma isoform	5.14
17222020	Bag2	BCL2-associated athanogene 2	5.14
17309457	Dzip1	DAZ interacting protein 1	5.14
17222625	Tgfbrap1	transforming growth factor, beta receptor associated protein 1	5.13
17497303	Fam196a	family with sequence similarity 196, member A	5.13
17548012	Gm6594	predicted pseudogene 6594 [Source:MGI Symbol;Acc:MGI:3643873]	5.12
17514355	Gm10715	predicted gene 10715 [Source:MGI Symbol;Acc:MGI:3642376]	5.1
17320854	LOC102634389; Gm17546	uncharacterized LOC102634389; predicted gene, 17546 [Source:MGI Symbol;Acc:MGI:4937180]	5.08
17418177	Heyl	hairy/enhancer-of-split related with YRPW motif-like	5.06
17441540	Med13l	mediator complex subunit 13-like	4.98
17532455	1110059G10Rik	RIKEN cDNA 1110059G10 gene	4.97
17463909	Mgst1	microsomal glutathione S-transferase 1	4.93
17512329	Fam96b	family with sequence similarity 96, member B	4.93
17400291	Fam63a	family with sequence similarity 63, member A	4.91
17483325	Fbrs	fibrosin	4.9
17444778	Gm3409	predicted gene 3409	4.89
17502525	Mir28b	microRNA 28b	4.87
17275976	Gm3086	RuvB-like protein 1 pseudogene	4.83
17357529	Ddb1	damage specific DNA binding protein 1	4.82

17352985	B4galt6	UDP-Gal:betaGlcNAc beta 1,4-galactosyltransferase, polypeptide 6	4.81
17218277	Nmnat2	nicotinamide nucleotide adenylyltransferase 2	4.8
17430319	S100pbp	S100P binding protein	4.79
17242330	Lrrc3	leucine rich repeat containing 3	4.76
17273116	Oxld1	oxidoreductase like domain containing 1	4.75
17424276	Enho	energy homeostasis associated	4.74
17394765	Gm24327	predicted gene, 24327 [Source:MGI Symbol;Acc:MGI:5454104]	4.73
17402025	Dbt	dihydrolipoamide branched chain transacylase E2	4.72
17547610			4.69
17272095	H3f3b	H3 histone, family 3B	4.68
17287576			4.67
17533469	Cypt8; Cypt7; Cypt1	cysteine-rich perinuclear theca 8; cysteine-rich perinuclear theca 7; cysteine-rich perinuclear theca 1	4.67
17354101	Dpysl3	dihydropyrimidinase-like 3	4.66
17319770	Poldip3	polymerase (DNA-directed), delta interacting protein 3	4.65
17524170	Cep295	centrosomal protein 295	4.64
17432790	Miip	migration and invasion inhibitory protein	4.62
17536657	Foxo4	forkhead box O4	4.62
17549270			4.61
17549306			4.61
17439741	Aff1	AF4/FMR2 family, member 1	4.6
17423461	Gm26148	predicted gene, 26148 [Source:MGI Symbol;Acc:MGI:5455925]	4.58
17549928			4.56
17283955	Gm26906	predicted gene, 26906 [Source:MGI Symbol;Acc:MGI:5477400]	4.55
17312780	Gga1	golgi associated, gamma adaptin ear containing, ARF binding protein 1	4.55
17455793	Gng11	guanine nucleotide binding protein (G protein), gamma 11	4.54
17257140	Arhgap27os2	Rho GTPase activating protein 27, opposite strand 2 [Source:MGI Symbol;Acc:MGI:3650160]	4.51
17256607	Ramp2	receptor (calcitonin) activity modifying protein 2	4.5
17261825	Mat2b	methionine adenosyltransferase II, beta	4.48
17517914	Nptn	neuroplastin	4.46
17499480	Kbtbd11	kelch repeat and BTB (POZ) domain containing 11	4.46
17363893	Ppp1r2-ps3	protein phosphatase 1, regulatory (inhibitor) subunit 2, pseudogene 3	4.46
17528079	2300009A05Rik	RIKEN cDNA 2300009A05 gene	4.46
17382681	Gm996	predicted gene 996	4.44
17441361	9530046B11Rik	RIKEN cDNA 9530046B11 gene [Source:MGI Symbol;Acc:MGI:1925671]	4.42
17304147	Zcchc24	zinc finger, CCHC domain containing 24	4.41
17524426	Fbxl12	F-box and leucine-rich repeat protein 12	4.41
17322473	Gm26675	predicted gene, 26675 [Source:MGI Symbol;Acc:MGI:5477169]	4.4
17252969	Rilp	Rab interacting lysosomal protein	4.33
17338200	Tbcc	tubulin-specific chaperone C	4.33
17445713			4.33
17245661	Mir378d	microRNA 378d	4.28
17237033	Gm25376	predicted gene, 25376 [Source:MGI Symbol;Acc:MGI:5455153]	4.27
17403237	Gbp3	guanylate binding protein 3	4.27
17360004	Tmem180; Gm36493	transmembrane protein 180; predicted gene, 36493	4.26
17311199	Dcaf13	DDB1 and CUL4 associated factor 13	4.24
17444047	Elfn1	leucine rich repeat and fibronectin type III, extracellular 1	4.24
17535191	Gm23000	predicted gene, 23000 [Source:MGI Symbol;Acc:MGI:5452777]	4.21
17527993	LOC102638888	uncharacterized LOC102638888	4.19
17390424	Lcmt2	leucine carboxyl methyltransferase 2	4.16
17371912	Zak	sterile alpha motif and leucine zipper containing kinase AZK	4.16
17539611	Gpm6b	glycoprotein m6b	4.16
17435811	Kcnk3	potassium channel, subfamily K, member 3	4.15
17269218	Gm14188	predicted gene 14188 [Source:MGI Symbol;Acc:MGI:3651817]	4.14
17465365	Prrt4	proline-rich transmembrane protein 4	4.13
17215817	Olfr12	olfactory receptor 12	4.11
17482719	Ubfd1	ubiquitin family domain containing 1	4.11
17214433	Ankzf1	ankyrin repeat and zinc finger domain containing 1	4.09
17303636	Rpl15	ribosomal protein L15	4.09
17304049	Dlg5	discs, large homolog 5 (Drosophila)	4.08
17362216	Nudt22	nudix (nucleoside diphosphate linked moiety X)-type motif 22	4.07
17531671	Tmie	transmembrane inner ear	4.05
17533282	Mid1ip1	Mid1 interacting protein 1 (gastrulation specific G12-like (zebrafish))	4.05

17294318	Gm10126	predicted gene 10126 [Source:MGI Symbol;Acc:MGI:3809072]	4.02
17297913	Arf4	ADP-ribosylation factor 4	4.01
17520146	Trim43c	tripartite motif-containing 43C	4
17336036	Zfp472	zinc finger protein 472	3.94
17383540	Zdhhc12	zinc finger, DHHC domain containing 12	3.94
17544779	Rab9b	RAB9B, member RAS oncogene family	3.94
17353796	Taf7	TAF7 RNA polymerase II, TATA box binding protein (TBP)-associated factor	3.93
17498867	Gm6410	predicted gene 6410	3.92
17341631	Prss33	protease, serine 33	3.9
17388901	D430041D05Rik	RIKEN cDNA D430041D05 gene	3.9
17418057	Gm24678	predicted gene, 24678 [Source:MGI Symbol;Acc:MGI:5454455]	3.88
17255949	Ppp1r1b	protein phosphatase 1, regulatory (inhibitor) subunit 1B	3.87
17533002	Mir1198	microRNA 1198	3.86
17244369	Gm8580	ribosomal protein L29 pseudogene	3.83
17539519	Pir	pirin	3.83
17549564			3.83
17549720			3.83
17500532	Gm4889	predicted gene 4889 [Source:MGI Symbol;Acc:MGI:3647233]	3.82
17340323	Foxn2	forkhead box N2	3.82
17377621	Tmem74b	transmembrane protein 74B	3.81
17222205	Ankrd39	ankyrin repeat domain 39	3.79
17451886	Fbxw8	F-box and WD-40 domain protein 8	3.79
17490532	Scaf1	SR-related CTD-associated factor 1	3.79
17309172	Mycbp2	MYC binding protein 2	3.78
17215291	3110079O15Rik	RIKEN cDNA 3110079O15 gene	3.77
17271719	Cd300c	CD300C antigen	3.77
17447610	Msx1	msh homeobox 1	3.76
17499255	Cul4a	cullin 4A	3.76
17510986	2210011C24Rik; Mir1199	RIKEN cDNA 2210011C24 gene; microRNA 1199	3.76
17294805	Zcchc9	zinc finger, CCHC domain containing 9	3.75
17336086	Adamts10	a disintegrin-like and metallopeptidase (reprolysin type) with thrombospondin type 1 motif, 10	3.74
17259157	Endov	endonuclease V	3.73
17419718	E130218I03Rik	RIKEN cDNA E130218I03 gene	3.7
17236705	Gm16155	predicted gene 16155 [Source:MGI Symbol;Acc:MGI:3801971]	3.68
17547769			3.68
17268846	LOC102634154; Gm12356	uncharacterized LOC102634154; predicted gene 12356 [Source:MGI Symbol;Acc:MGI:3651195]	3.67
17372894	Olfr1158	olfactory receptor 1158	3.67
17476620	Gm6669	predicted pseudogene 6669 [Source:MGI Symbol;Acc:MGI:3646884]	3.67
17343688	B3galt4	UDP-Gal:betaGlcNAc beta 1,3-galactosyltransferase, polypeptide 4	3.66
17269845	Becn1	beclin 1, autophagy related	3.64
17301849	Gm16549	predicted gene 16549 [Source:MGI Symbol;Acc:MGI:4414969]	3.62
17272798	Cant1	calcium activated nucleotidase 1	3.61
17295005	Bhmt	betaine-homocysteine methyltransferase	3.6
17334474	Spsb3	splA/ryanodine receptor domain and SOCS box containing 3	3.59
17431053	Zdhhc18;Gm13213	zinc finger, DHHC domain containing 18; predicted gene 13213	3.58
17468895	Chst13	carbohydrate (chondroitin 4) sulfotransferase 13	3.56
17328333	Gm15807	predicted gene 15807 [Source:MGI Symbol;Acc:MGI:3802044]	3.53
17309345	Gm6219	predicted gene 6219	3.5
17344221	Csnk2b	casein kinase 2, beta polypeptide	3.49
17280315	Rnf144a	ring finger protein 144A	3.48
17485528	Tfpt	TCF3 (E2A) fusion partner	3.48
17523279			3.48
17374669	Bahd1	bromo adjacent homology domain containing 1	3.47
17393579	Sla2	Src-like-adaptor 2	3.46
17460861	Slc41a3	solute carrier family 41, member 3	3.45
17448880	Lnx1	ligand of numb-protein X 1	3.44
17405062	Pabpc4l	poly(A) binding protein, cytoplasmic 4-like	3.44
17443286	Por	P450 (cytochrome) oxidoreductase	3.43
17388284	Mdk	midkine	3.42
17372013	Chn1os3	chimerin 1, opposite strand 3	3.42
17228490	Fam163a	family with sequence similarity 163, member A	3.4
17346503	Tubb4a	tubulin, beta 4A class IVA	3.4

17366244	LOC100041057	sp110 nuclear body protein-like	3.39
17363156	Gm22323	predicted gene, 22323 [Source:MGI Symbol;Acc:MGI:5452100]	3.36
17363158	Gm23931	predicted gene, 23931 [Source:MGI Symbol;Acc:MGI:5453708]	3.36
17455801	Col1a2	collagen, type I, alpha 2	3.36
17458385	Gimap5	GTPase, IMAP family member 5	3.36
17492196	Slco3a1	solute carrier organic anion transporter family, member 3a1	3.36
17549488			3.35
17301262	Rp1l1	retinitis pigmentosa 1 homolog (human)-like 1	3.35
17358043	D030056L22Rik	RIKEN cDNA D030056L22 gene	3.35
17362753	Cd5	CD5 antigen	3.35
17382574	BC029214	cDNA sequence BC029214	3.35
17238898	Syne1	spectrin repeat containing, nuclear envelope 1	3.34
17296174	Map3k1	mitogen-activated protein kinase kinase kinase 1	3.34
17324303			3.34
17407363	S100a9	S100 calcium binding protein A9 (calgranulin B)	3.34
17476521	Tmem147os; Sbsn	transmembrane protein 147, opposite strand; suprabasin	3.34
17549792			3.34
17367683	Gm13411	predicted gene 13411 [Source:MGI Symbol;Acc:MGI:3651013]	3.32
17377744	Rem1	rad and gem related GTP binding protein 1	3.32
17390980	Shc4	SHC (Src homology 2 domain containing) family, member 4	3.32
17446832	Ucn	urocortin	3.32
17413191	Dnajb5	DnaJ (Hsp40) homolog, subfamily B, member 5	3.31
17217821	Zfp281	zinc finger protein 281	3.3
17394679	Ptgis	prostaglandin I2 (prostacyclin) synthase	3.3
17273948	Sdc1	syndecan 1	3.29
17393743	Ghrh	growth hormone releasing hormone	3.28
17421571	Mad2l2	MAD2 mitotic arrest deficient-like 2	3.28
17248406	Gm12121	predicted gene 12121	3.27
17236733	5730420D15Rik	RIKEN cDNA 5730420D15 gene	3.27
17283380	Fbln5	fibulin 5	3.27
17434087	LOC100043918	C-C motif chemokine 19-like	3.27
17531001	Sema3f	sema domain, immunoglobulin domain (Ig), short basic domain, secreted, (semaphorin) 3F	3.25
17405463	P2ry14	purinergic receptor P2Y, G-protein coupled, 14	3.24
17459960	Rtkn	rhotekin	3.23
17467527	Gm24096	predicted gene, 24096 [Source:MGI Symbol;Acc:MGI:5453873]	3.22
17501818	Borcs8	BLOC-1 related complex subunit 8	3.22
17313647	Bik	BCL2-interacting killer	3.21
17449718	Cxcl10	chemokine (C-X-C motif) ligand 10	3.21
17397120	Bbs12	Bardet-Biedl syndrome 12 (human)	3.2
17508775	Gm16793	predicted gene, 16793	3.2
17356425	Gm25432	predicted gene, 25432	3.18
17428702	Mmachc	methylmalonic aciduria cblC type, with homocystinuria	3.18
17350790	Prrc1	proline-rich coiled-coil 1	3.17
17289860	Gm15323;Gm15322	predicted gene 15323; predicted gene 15322	3.16
17357389	Ahnak	AHNAK nucleoprotein (desmoyokin)	3.16
17231188	Diexf	digestive organ expansion factor homolog (zebrafish)	3.15
17320337	Plxnb2	plexin B2	3.15
17513965	Dbndd1	dysbindin (dystrobrevin binding protein 1) domain containing 1	3.15
17483007	Sbk1	SH3-binding kinase 1	3.14
17243252	Tjp3	tight junction protein 3	3.13
17347505	Dhx57	DEAH (Asp-Glu-Ala-Asp/His) box polypeptide 57	3.13
17518109	Uaca	uveal autoantigen with coiled-coil domains and ankyrin repeats	3.13
17547998			3.13
17384008	Cfap157	cilia and flagella associated protein 157	3.12
17549368			3.12
17549746			3.12
17301061	Mipep	mitochondrial intermediate peptidase	3.11
17406433	Prss48	protease, serine 48	3.11
17326844	Gm25715	predicted gene, 25715 [Source:MGI Symbol;Acc:MGI:5455492]	3.1
17242531	Gm9978	predicted gene 9978 [Source:MGI Symbol;Acc:MGI:3641806]	3.09
17336322	Rxrb	retinoid X receptor beta	3.09
17490282	Pold1	polymerase (DNA directed), delta 1, catalytic subunit	3.09
17548561	Hist4h4	histone cluster 4, H4	3.09

17415835	Ror1	receptor tyrosine kinase-like orphan receptor 1	3.07
17451952	Gm10390	predicted gene 10390	3.07
17224395	Mir375	microRNA 375	3.06
17379427	Dbndd2	dysbindin (dystrobrevin binding protein 1) domain containing 2	3.06
17270684	10-Mar	membrane-associated ring finger (C3HC4) 10	3.05
17451649	Acads	acyl-Coenzyme A dehydrogenase, short chain	3.05
17256323	Krtap31-1	keratin associated protein 31-1	3.04
17531939	Gpd1l	glycerol-3-phosphate dehydrogenase 1-like	3.03
17343672			3.02
17258093	Gprc5c	G protein-coupled receptor, family C, group 5, member C	3.01
17432347	Fhad1	forkhead-associated (FHA) phosphopeptide binding domain 1	3.01
17461312	Trnt1	tRNA nucleotidyl transferase, CCA-adding, 1	3.01
17506949	Pard3	par-3 family cell polarity regulator	3.01
17476443	Arhgap33os	Rho GTPase activating protein 33, opposite strand	3
17432082	Crocc	ciliary rootlet coiled-coil, rootletin	2.99
17548369	LOC102637947; Gm13035	60S acidic ribosomal protein P1-like; predicted gene 13035 [Source:MGI Symbol;Acc:MGI:3650251]	2.98
17397497	Foxo1	forkhead box O1	2.98
17300565	Rnf31	ring finger protein 31	2.98
17395640	B230312C02Rik	RIKEN cDNA B230312C02 gene	2.98
17439897	Gm26519	predicted gene, 26519	2.98
17450952	Gtpbp6	GTP binding protein 6 (putative)	2.97
17324619	4632428C04Rik	RIKEN cDNA 4632428C04 gene	2.96
17500060	Mir486	microRNA 486	2.96
17529358	Gm5619	predicted gene 5619 [Source:MGI Symbol;Acc:MGI:3643807]	2.96
17293961	Zfp429	zinc finger protein 429	2.95
17313998	Creld2	cysteine-rich with EGF-like domains 2	2.95
17227532	Gm23534	predicted gene, 23534 [Source:MGI Symbol;Acc:MGI:5453311]	2.94
17321094	Col2a1	collagen, type II, alpha 1	2.94
17547682			2.94
17320163	Brd1	bromodomain containing 1	2.93
17299196			2.92
17273276	Gm17178	predicted gene 17178 [Source:MGI Symbol;Acc:MGI:4938005]	2.91
17247064	Gm11967	predicted gene 11967	2.9
17309118	Tbc1d4	TBC1 domain family, member 4	2.89
17380199	Rbm38	RNA binding motif protein 38	2.89
17279427	Btbd6	BTB (POZ) domain containing 6	2.88
17242700	Prss57	protease, serine 57	2.88
17276743	Plekhh1	pleckstrin homology domain containing, family H (with MyTH4 domain) member 1	2.88
17301852	Gm9199	glycine cleavage system protein H (aminomethyl carrier) pseudogene	2.88
17322507	Dnase1	deoxyribonuclease I	2.88
17382767	Sohlh1	spermatogenesis and oogenesis specific basic helix-loop-helix 1	2.88
17407716	Tnfaip8l2	tumor necrosis factor, alpha-induced protein 8-like 2	2.88
17427767	Gm26047	predicted gene, 26047 [Source:MGI Symbol;Acc:MGI:5455824]	2.88
17432674	Tnfrsf1b	tumor necrosis factor receptor superfamily, member 1b	2.88
17403806	Erich3	glutamate rich 3	2.87
17234711	Gm10142	predicted gene 10142	2.87
17382384	Tor4a	torsin family 4, member A	2.87
17480566	n-R5s156	nuclear encoded rRNA 5S 156 [Source:MGI Symbol;Acc:MGI:4422019]	2.87
17544969	Col4a6	collagen, type IV, alpha 6	2.86
17266424	Foxn1	forkhead box N1	2.85
17500301	Adgra2	adhesion G protein-coupled receptor A2	2.85
17545075	Gm15032	predicted gene 15032	2.85
17339079	Gm24813	predicted gene, 24813 [Source:MGI Symbol;Acc:MGI:5454590]	2.84
17258041	Dnaic2	dynein, axonemal, intermediate chain 2	2.84
17336681	Tnxb	tenascin XB	2.84
17398571	Tmem154	transmembrane protein 154	2.83
17491699	Mir344d-2	microRNA 344d-2	2.82
17232800	Gm25526	predicted gene, 25526 [Source:MGI Symbol;Acc:MGI:5455303]	2.81
17295981	Fam159b	family with sequence similarity 159, member B	2.81
17418976	Sync	syncoilin	2.81
17476535	Dmkn	dermokine	2.81
17286905	Cd83	CD83 antigen	2.8
17374632	Knstrn	kinetochore-localized astrin/SPAG5 binding	2.8

17510462	Cyp4f18	cytochrome P450, family 4, subfamily f, polypeptide 18	2.8
17224614	Gm15178	predicted gene 15178 [Source:MGI Symbol;Acc:MGI:3705132]	2.78
17408088			2.78
17264363	Usp43	ubiquitin specific peptidase 43	2.77
17248646	Gm12153	predicted gene 12153	2.77
17418271	Fhl3	four and a half LIM domains 3	2.77
17427401	Cyp2j9	cytochrome P450, family 2, subfamily j, polypeptide 9	2.77
17500289	Prosc	proline synthetase co-transcribed	2.77
17494117	Trim68	tripartite motif-containing 68	2.76
17232731	Rnu3a	U3A small nuclear RNA	2.76
17548398			2.76
17415897	Raver2	ribonucleoprotein, PTB-binding 2	2.75
17357914	Olfr1467	olfactory receptor 1467	2.74
17371990	Gm13703	predicted gene 13703 [Source:MGI Symbol;Acc:MGI:3651307]	2.74
17440342	Gm15446	predicted gene 15446	2.73
17339499	Snord53	small nucleolar RNA, C/D box 53	2.72
17430270	Tlr12	toll-like receptor 12	2.72
17516617	LOC102641249; C030014I23Rik	uncharacterized LOC102641249; RIKEN cDNA C030014I23 gene [Source:MGI Symbol;Acc:MGI:1924631]	2.72
17341803	Abca17	ATP-binding cassette, sub-family A (ABC1), member 17	2.71
17379533	Snx21	sorting nexin family member 21	2.71
17444027	Uncx	UNC homeobox	2.71
17455962	C1galt1	core 1 synthase, glycoprotein-N-acetylgalactosamine 3-beta-galactosyltransferase, 1	2.71
17351465	Tubb6	tubulin, beta 6 class V	2.7
17404230	Gm5150	predicted gene 5150	2.7
17450989	Pxmp2	peroxisomal membrane protein 2	2.7
17549366			2.7
17549760			2.7
17306625	Zfhx2	zinc finger homeobox 2	2.68
17547141	Gm20994	predicted gene, 20994	2.68
17292562	Sema4d	sema domain, immunoglobulin domain (Ig), transmembrane domain (TM) and short cytoplasmic domain, (semaphorin) 4D	2.67
17253937	Rhbdl3	rhomboid, veinlet-like 3 (Drosophila)	2.67
17309011	Gm24127	predicted gene, 24127 [Source:MGI Symbol;Acc:MGI:5453904]	2.67
17352114			2.67
17352116			2.67
17457892	Casp2	caspase 2	2.67
17548456			2.67
17326814	Gm25908	predicted gene, 25908 [Source:MGI Symbol;Acc:MGI:5455685]	2.66
17547656			2.66
17248992	Flt4	FMS-like tyrosine kinase 4	2.66
17461112			2.66
17547652			2.66
17271158	Cacng5	calcium channel, voltage-dependent, gamma subunit 5	2.65
17348666	4933424G05Rik	RIKEN cDNA 4933424G05 gene	2.65
17401345	Ppm1j	protein phosphatase 1J	2.65
17547475			2.65
17548553			2.65
17365566			2.64
17525836	Gm16095	predicted gene 16095 [Source:MGI Symbol;Acc:MGI:3801984]	2.63
17538281	Gm15295	predicted gene 15295 [Source:MGI Symbol;Acc:MGI:3705286]	2.63
17485943	Shisa7	shisa family member 7	2.62
17245044	Tmem19	transmembrane protein 19	2.62
17319150	Baiap2l2	BAI1-associated protein 2-like 2	2.62
17438903	Afp	alpha fetoprotein	2.62
17440361	Plcxd1	phosphatidylinositol-specific phospholipase C, X domain containing 1	2.6
17219182			2.6
17474544	1700058P15Rik	RIKEN cDNA 1700058P15 gene [Source:MGI Symbol;Acc:MGI:1920627]	2.6
17355139	Gm19784	predicted gene, 19784	2.59
17415375	Gm12603	predicted gene 12603	2.59
17550442			2.59
17221934	Gm9898	predicted gene 9898 [Source:MGI Symbol;Acc:MGI:3642391]	2.58
17411871	1700123M08Rik	RIKEN cDNA 1700123M08 gene	2.57
17487249	Mark4	MAP/microtubule affinity regulating kinase 4	2.56

17255626	Skap1; Gm38448	src family associated phosphoprotein 1; predicted gene, 38448	2.56
17267482	Gm22883	predicted gene, 22883 [Source:MGI Symbol;Acc:MGI:5452660]	2.56
17365690	Gm10197	predicted gene 10197 [Source:MGI Symbol;Acc:MGI:3704501]	2.56
17526923	Hspb2	heat shock protein 2	2.56
17341712	Amdhd2	amidohydrolase domain containing 2	2.55
17459327	Igkv2-116	immunoglobulin kappa variable 2-116	2.55
17466386	Fam131b	family with sequence similarity 131, member B	2.54
17272785	Lgals3bp	lectin, galactoside-binding, soluble, 3 binding protein	2.54
17392549	9030622O22Rik	RIKEN cDNA 9030622O22 gene	2.54
17402410			2.54
17444851	D5Ertd605e	DNA segment, Chr 5, ERATO Doi 605, expressed	2.54
17299575	Ang; Rnase4	angiogenin, ribonuclease, RNase A family, 5; ribonuclease, RNase A family 4	2.53
17327450	Kcnj15	potassium inwardly-rectifying channel, subfamily J, member 15	2.53
17396349	Spata16	spermatogenesis associated 16	2.53
17293465			2.52
17361558	Sart1	squamous cell carcinoma antigen recognized by T cells 1	2.52
17425384	Gm12514	predicted gene 12514	2.52
17521014	Acad11	acyl-Coenzyme A dehydrogenase family, member 11	2.52
17545904			2.51
17301108	Arl11	ADP-ribosylation factor-like 11	2.51
17265062	2810408A11Rik	RIKEN cDNA 2810408A11 gene	2.51
17344728	H2-M10.1	histocompatibility 2, M region locus 10.1	2.51
17428223	Dmrta2os	doublesex and mab-3 related transcription factor like family A2, opposite strand	2.51
17471270	9330102E08Rik	RIKEN cDNA 9330102E08 gene	2.51
17538141	Pih1h3b	PIH1 domain containing 3B	2.51
17233032	1700027J07Rik	RIKEN cDNA 1700027J07 gene	2.5
17458669	9430076C15Rik; Creb5	RIKEN cDNA 9430076C15 gene; cAMP responsive element binding protein 5	2.5
17365106	Sec31b	Sec31 homolog B (S. cerevisiae)	2.49
17425855	Inip	INTS3 and NABP interacting protein	2.49
17492495	Cib1	calcium and integrin binding 1 (calmyrin)	2.49
17530884	Hemk1	HemK methyltransferase family member 1	2.48
17305738	Gm10101	predicted gene 10101 [Source:MGI Symbol;Acc:MGI:3641713]	2.47
17326905	2810407A14Rik	RIKEN cDNA 2810407A14 gene [Source:MGI Symbol;Acc:MGI:1917461]	2.47
17407452	Lce1f	late cornified envelope 1F	2.47
17473779	Zfp128	zinc finger protein 128	2.47
17509315	Tenm3	teneurin transmembrane protein 3	2.47
17532409	Cyp8b1	cytochrome P450, family 8, subfamily b, polypeptide 1	2.47
17235211	Midn	midnolin	2.46
17252800	Mettl16	methyltransferase like 16	2.46
17280287	9030624G23Rik	RIKEN cDNA 9030624G23 gene	2.46
17321811	Fignl2	fidgetin-like 2	2.46
17365193	Lbx1	ladybird homeobox homolog 1 (Drosophila)	2.46
17485497	Nlrp12	NLR family, pyrin domain containing 12	2.46
17284105	Gm10425	predicted gene 10425 [Source:MGI Symbol;Acc:MGI:3642471]	2.45
17320553	LOC105246018; Gm15609	uncharacterized LOC105246018; predicted gene 15609 [Source:MGI Symbol;Acc:MGI:3783055]	2.45
17426890	Psip1	PC4 and SFRS1 interacting protein 1	2.45
17427792	Tmem61	transmembrane protein 61	2.45
17218736	Vamp4	vesicle-associated membrane protein 4	2.44
17527694	Loxl1	lysyl oxidase-like 1	2.44
17214899	Gm7609	predicted pseudogene 7609	2.43
17237681	9230105E05Rik	RIKEN cDNA 9230105E05 gene	2.43
17250855	9630013K17Rik	RIKEN cDNA 9630013K17 gene	2.43
17266882	LOC102635154	uncharacterized LOC102635154	2.43
17312260	Rhpn1	rhophilin, Rho GTPase binding protein 1	2.43
17454627	Card11	caspase recruitment domain family, member 11	2.43
17516915	Apoa5	apolipoprotein A-V	2.43
17519898	Sh3bgrl2	SH3 domain binding glutamic acid-rich protein like 2	2.43
17305105	4930596D02Rik	RIKEN cDNA 4930596D02 gene	2.42
17332804	Synj2	synaptojanin 2	2.42
17440971	Gm13790	predicted gene 13790	2.42
17467040	Crhr2	corticotropin releasing hormone receptor 2	2.42
17485932	Tmem238	transmembrane protein 238	2.42
17221923	n-R5s209	nuclear encoded rRNA 5S 209 [Source:MGI Symbol;Acc:MGI:4422074]	2.41

17365718	Gm16299	predicted gene 16299	2.41
17274942	Efcab10	EF-hand calcium binding domain 10	2.41
17338286	Usp49	ubiquitin specific peptidase 49	2.41
17502583	Mcm5	minichromosome maintenance deficient 5, cell division cycle 46 (S. cerevisiae)	2.41
17224386	Cryba2	crystallin, beta A2	2.4
17300591	Irf9	interferon regulatory factor 9	2.4
17338245	Guca1b	guanylate cyclase activator 1B	2.4
17414348	Gm12536	predicted gene 12536	2.4
17221197	Tcf24	transcription factor 24	2.39
17265277	Vmo1	vitelline membrane outer layer 1 homolog (chicken)	2.39
17272174	Trim65; Trim47	tripartite motif-containing 65; tripartite motif-containing 47	2.39
17250089	Olfr322	olfactory receptor 322	2.38
17267446	Smg8	smg-8 homolog, nonsense mediated mRNA decay factor (C. elegans)	2.38
17389467	Grem1	gremlin 1	2.38
17255372	Fam117a	family with sequence similarity 117, member A	2.37
17272509	Mxra7	matrix-remodelling associated 7	2.37
17284527	Ighv1-18	immunoglobulin heavy variable V1-18	2.37
17301576	Gnrh1	gonadotropin releasing hormone 1	2.37
17317284	Anxa13	annexin A13	2.37
17382603	Lcn12	lipocalin 12	2.37
17382737	Fcna	ficolin A	2.37
17447689	Zfp518b	zinc finger protein 518B	2.37
17472530	Kcnj8	potassium inwardly-rectifying channel, subfamily J, member 8	2.37
17378035	Bpifb6	BPI fold containing family B, member 6	2.36
17309157	4933432I03Rik	RIKEN cDNA 4933432I03 gene	2.36
17325044	Slc12a8	solute carrier family 12 (potassium/chloride transporters), member 8	2.36
17471062	Tapbpl	TAP binding protein-like	2.36
17492051	Arrdc4	arrestin domain containing 4	2.36
17505934	Gm15395	predicted gene 15395	2.36
17546620	Gm28890	predicted gene 28890 [Source:MGI Symbol;Acc:MGI:5579596]	2.36
17406358	4930565D16Rik	RIKEN cDNA 4930565D16 gene	2.35
17489441	Hpn	hepsin	2.35
17474676	Gm16175	predicted gene 16175 [Source:MGI Symbol;Acc:MGI:3801805]	2.34
17283303	Ccdc88c	coiled-coil domain containing 88C	2.34
17315422	Amhr2	anti-Mullerian hormone type 2 receptor	2.34
17332107	4932438H23Rik	RIKEN cDNA 4932438H23 gene	2.34
17365861	Gm6990	predicted pseudogene 6990 [Source:MGI Symbol;Acc:MGI:3648860]	2.34
17454659	Gm16036	predicted gene 16036	2.34
17489813	Pdcd5	programmed cell death 5	2.34
17545179	Gm26151	predicted gene, 26151 [Source:MGI Symbol;Acc:MGI:5455928]	2.34
17435660	Nom1	nucleolar protein with MIF4G domain 1	2.33
17229162	Gm16548	predicted gene 16548	2.33
17344322	Nfkbil1	nuclear factor of kappa light polypeptide gene enhancer in B cells inhibitor like 1	2.33
17440885	Myo1h	myosin 1H	2.33
17476349	Syne4	spectrin repeat containing, nuclear envelope family member 4	2.33
17515869	Gm23702	predicted gene, 23702 [Source:MGI Symbol;Acc:MGI:5453479]	2.32
17242232	Col18a1	collagen, type XVIII, alpha 1	2.32
17354463	3-Mar	membrane-associated ring finger (C3HC4) 3	2.32
17232112	C920009B18Rik	RIKEN cDNA C920009B18 gene	2.31
17282932			2.31
17302376	Pibf1	progesterone immunomodulatory binding factor 1	2.31
17381617	Gm13261	predicted gene 13261	2.31
17389672	Gm13985	predicted gene 13985	2.31
17425010	Gm12446	predicted gene 12446 [Source:MGI Symbol;Acc:MGI:3651433]	2.31
17444016	Gper1	G protein-coupled estrogen receptor 1	2.31
17447646	Slc2a9	solute carrier family 2 (facilitated glucose transporter), member 9	2.31
17549848			2.31
17391909	5330413P13Rik	RIKEN cDNA 5330413P13 gene	2.3
17305198	Sh2d4b	SH2 domain containing 4B	2.3
17305143	Cdhr1	cadherin-related family member 1	2.3
17454915	Gm17135	predicted gene 17135 [Source:MGI Symbol;Acc:MGI:4937962]	2.3
17456285	Lsm8	LSM8 homolog, U6 small nuclear RNA associated (S. cerevisiae)	2.3
17549252			2.3
17329774	0610012G03Rik	RIKEN cDNA 0610012G03 gene	2.29

17335281	Def6	differentially expressed in FDCP 6	2.29
17473985	Obox4-ps33	oocyte specific homeobox 4, pseudogene 33	2.29
17539210	Sh3kbp1	SH3-domain kinase binding protein 1	2.29
17549006			2.29
17549134			2.29
17389962	Rpap1	RNA polymerase II associated protein 1	2.28
17243379	S1pr4	sphingosine-1-phosphate receptor 4	2.28
17376856	Gm14055	predicted gene 14055	2.28
17381457	Echdc3	enoyl Coenzyme A hydratase domain containing 3	2.28
17422764	Cpsf3l	cleavage and polyadenylation specific factor 3-like	2.28
17427081	C87499	expressed sequence C87499	2.28
17454687	Papolb	poly (A) polymerase beta (testis specific)	2.28
17471464	Klrb1b	killer cell lectin-like receptor subfamily B member 1B	2.28
17514566	Mmp27	matrix metallopeptidase 27	2.28
17297189	Gm11100	predicted gene 11100	2.27
17460707	4933427D06Rik	RIKEN cDNA 4933427D06 gene	2.27
17484789	Rassf7	Ras association (RalGDS/AF-6) domain family (N-terminal) member 7	2.27
17511534	Snx20	sorting nexin 20	2.27
17304740	Sh3bp5	SH3-domain binding protein 5 (BTK-associated)	2.26
17306906	Ripk3	receptor-interacting serine-threonine kinase 3	2.26
17217789	Kif14	kinesin family member 14	2.26
17241257	Col13a1	collagen, type XIII, alpha 1	2.26
17248249	Mpg	N-methylpurine-DNA glycosylase	2.26
17249168	Gm26542	predicted gene, 26542 [Source:MGI Symbol;Acc:MGI:5477036]	2.26
17300149	Traj52	T cell receptor alpha joining 52	2.26
17302213	Mtrf1	mitochondrial translational release factor 1	2.26
17333891	Gm7736	predicted gene 7736 [Source:MGI Symbol;Acc:MGI:3647086]	2.26
17413847	Gm16731	predicted gene, 16731	2.26
17451622	Gm9936	predicted gene 9936	2.26
17497313	Gm24581	predicted gene, 24581 [Source:MGI Symbol;Acc:MGI:5454358]	2.26
17549066			2.26
17413339	Ccdc107	coiled-coil domain containing 107	2.25
17509537	Palld	palladin, cytoskeletal associated protein	2.25
17535627	Abcd1	ATP-binding cassette, sub-family D (ALD), member 1	2.25
17352401	Map3k8	mitogen-activated protein kinase kinase kinase 8	2.24
17360731	Gm10007	predicted gene 10007	2.24
17396205	Mtfr1	mitochondrial fission regulator 1	2.24
17405746	Rarres1	retinoic acid receptor responder (tazarotene induced) 1	2.24
17469016	Wnt7a	wingless-type MMTV integration site family, member 7A	2.24
17252038	Zmynd15	zinc finger, MYND-type containing 15	2.23
17347896	Gm24648	predicted gene, 24648 [Source:MGI Symbol;Acc:MGI:5454425]	2.23
17378216	Cbfa2t2	core-binding factor, runt domain, alpha subunit 2, translocated to, 2 (human)	2.23
17395182	Gm14617	predicted gene 14617 [Source:MGI Symbol;Acc:MGI:3705092]	2.23
17427441	Gm10192	predicted gene 10192 [Source:MGI Symbol;Acc:MGI:3642867]	2.23
17433461	Hes3	hairy and enhancer of split 3 (Drosophila)	2.23
17439464	Anxa3	annexin A3	2.23
17492179	Gm10619	predicted gene 10619	2.23
17549920			2.23
17280431	Tpo	thyroid peroxidase	2.22
17305585	Ptgdr	prostaglandin D receptor	2.22
17390561	Catsper2	cation channel, sperm associated 2	2.22
17518563	Rasl12	RAS-like, family 12	2.22
17384202	9430024E24Rik	RIKEN cDNA 9430024E24 gene	2.21
17217232	Tmem81	transmembrane protein 81	2.21
17221436	Sbspon	somatomedin B and thrombospondin, type 1 domain containing	2.21
17252995	Slc43a2	solute carrier family 43, member 2	2.21
17341365	Zfp820	zinc finger protein 820	2.21
17439092	Gm23031	predicted gene, 23031 [Source:MGI Symbol;Acc:MGI:5452808]	2.21
17459449	Foxi3	forkhead box I3	2.2
17312418	Hgh1	HGH1 homolog	2.2
17357126	Snhg1	small nucleolar RNA host gene 1	2.2
17400885	Wars2; Gm38468	tryptophanyl tRNA synthetase 2 (mitochondrial); predicted gene, 38468	2.2
17420811	LOC102636380; Gm1667	uncharacterized LOC102636380; predicted gene 1667 [Source:MGI Symbol;Acc:MGI:2686513]	2.2

17431166	Lin28a	lin-28 homolog A (C. elegans)	2.2
17457685	Trbv12-1	T cell receptor beta, variable 12-1	2.2
17476618	Gm29627	predicted gene 29627 [Source:MGI Symbol;Acc:MGI:5580333]	2.2
17503254	Klf1	Kruppel-like factor 1 (erythroid)	2.2
17523985	4930568E12Rik	RIKEN cDNA 4930568E12 gene	2.2
17369060	Set	SET nuclear oncogene	2.19
17374373	C130080G10Rik	RIKEN cDNA C130080G10 gene	2.19
17414184	4930522O17Rik	RIKEN cDNA 4930522O17 gene	2.19
17522955	Gm16295	predicted gene 16295 [Source:MGI Symbol;Acc:MGI:3826546]	2.19
17323968	Vwa5b2	von Willebrand factor A domain containing 5B2	2.18
17265329	Spag7	sperm associated antigen 7	2.18
17291074	Vmn1r193	vomeronasal 1 receptor 193	2.18
17293265	Spata31d1d	spermatogenesis associated 31 subfamily D, member 1D	2.18
17319707	Cyp2d26	cytochrome P450, family 2, subfamily d, polypeptide 26	2.18
17421187	Pramef8	PRAME family member 8	2.18
17459801	Gcfc2	GC-rich sequence DNA binding factor 2	2.18
17220866	Rd3	retinal degeneration 3	2.17
17291635	A530084C06Rik	RIKEN cDNA A530084C06 gene [Source:MGI Symbol;Acc:MGI:3704402]	2.17
17355722	Sall3	sal-like 3 (Drosophila)	2.17
17366393	Acbd7	acyl-Coenzyme A binding domain containing 7	2.17
17399162	Gm25945	predicted gene, 25945 [Source:MGI Symbol;Acc:MGI:5455722]	2.17
17535445	Gm14684	predicted gene 14684 [Source:MGI Symbol;Acc:MGI:3705272]	2.17
17540116	Ssxb3	synovial sarcoma, X member B, breakpoint 3	2.17
17211885	Vwa3b	von Willebrand factor A domain containing 3B	2.16
17256513	Hsd17b1	hydroxysteroid (17-beta) dehydrogenase 1	2.16
17265229	Alox15	arachidonate 15-lipoxygenase	2.16
17276800	Rad51b	RAD51 homolog B	2.16
17325132	Gm15829	predicted gene 15829 [Source:MGI Symbol;Acc:MGI:3801893]	2.16
17454278	Gm454	predicted gene 454	2.16
17480216	Prcp	prolylcarboxypeptidase (angiotensinase C)	2.16
17502051	Il12rb1	interleukin 12 receptor, beta 1	2.16
17505009	Gm16156	predicted gene 16156 [Source:MGI Symbol;Acc:MGI:3801720]	2.16
17512611	Dpep2	dipeptidase 2	2.16
17514592	Mmp7	matrix metallopeptidase 7	2.16
17518316	Gm23136	predicted gene, 23136 [Source:MGI Symbol;Acc:MGI:5452913]	2.16
17549072			2.16
17281885	Six4	sine oculis-related homeobox 4	2.15
17314556	Slc48a1	solute carrier family 48 (heme transporter), member 1	2.15
17318807	Apol10b	apolipoprotein L 10B	2.15
17328573	Vpreb1	pre-B lymphocyte gene 1	2.15
17422404	Ttc34	tetratricopeptide repeat domain 34	2.15
17469455	Prok2	prokineticin 2	2.15
17483772	Sec23ip	Sec23 interacting protein	2.15
17498426	Ppfia1	protein tyrosine phosphatase, receptor type, f polypeptide (PTPRF), interacting protein (liprin), alpha 1	2.15
17521971	Slc26a6	solute carrier family 26, member 6	2.15
17549016			2.15
17217562	Gm10535	predicted gene 10535 [Source:MGI Symbol;Acc:MGI:3642442]	2.14
17287325	S1pr3	sphingosine-1-phosphate receptor 3	2.14
17349082			2.14
17431667	Gm13003	predicted gene 13003	2.14
17433009	Pex14	peroxisomal biogenesis factor 14	2.14
17486145	Zfp773	zinc finger protein 773	2.14
17499436	Cln8	ceroid-lipofuscinosis, neuronal 8	2.13
17434705	Gm8906	predicted gene 8906 [Source:MGI Symbol;Acc:MGI:3779820]	2.13
17211087	Gm15818	predicted gene 15818 [Source:MGI Symbol;Acc:MGI:3801960]	2.13
17305133	Rgr	retinal G protein coupled receptor	2.13
17317574	Hhla1	HERV-H LTR-associating 1	2.13
17320811	Gm23129	predicted gene, 23129 [Source:MGI Symbol;Acc:MGI:5452906]	2.13
17344608	H2-T3; Gm38417; H2-T18	histocompatibility 2, T region locus 3; predicted gene, 38417; histocompatibility 2, T region locus 18	2.13
17355270	4930546C10Rik	RIKEN cDNA 4930546C10 gene	2.13
17362708	Tmem138	transmembrane protein 138	2.13
17363666	Gm24252	predicted gene, 24252 [Source:MGI Symbol;Acc:MGI:5454029]	2.13

17483098	Gdpd3	glycerophosphodiester phosphodiesterase domain containing 3	2.13
17274310	Hpcal1	hippocalcin-like 1	2.12
17256051	Zpbp2	zona pellucida binding protein 2	2.12
17308759	4930444M15Rik	RIKEN cDNA 4930444M15 gene	2.12
17356305	Zdhhc24	zinc finger, DHHC domain containing 24	2.12
17412689	Gm12364	predicted gene 12364 [Source:MGI Symbol;Acc:MGI:3649785]	2.12
17503429	Olfr371	olfactory receptor 371	2.12
17537112	Lpar4	lysophosphatidic acid receptor 4	2.12
17275950	4931403G20Rik	RIKEN cDNA 4931403G20 gene	2.11
17285386	Psma2	proteasome (prosome, macropain) subunit, alpha type 2	2.11
17303685	Gm5458	predicted gene 5458	2.11
17392828	Gm14167	predicted gene 14167	2.11
17447041	Gm15614	predicted gene 15614	2.11
17461080	Gm22840	predicted gene, 22840 [Source:MGI Symbol;Acc:MGI:5452617]	2.11
17471201	Rad51ap1	RAD51 associated protein 1	2.11
17497003	Etos1	ectopic ossification 1	2.11
17511806	4930488L21Rik	RIKEN cDNA 4930488L21 gene	2.11
17524703	Ap1m2	adaptor protein complex AP-1, mu 2 subunit	2.11
17215879	Aqp12	aquaporin 12	2.1
17289977	Mcidas	multiciliate differentiation and DNA synthesis associated cell cycle protein	2.1
17332287	LOC102633000; 2310043M15Rik	uncharacterized LOC102633000; RIKEN cDNA 2310043M15 gene [Source:MGI Symbol;Acc:MGI:1919180]	2.1
17406682	1700113A16Rik	RIKEN cDNA 1700113A16 gene	2.1
17450802	Dgkq	diacylglycerol kinase, theta	2.1
17457683	Trbv10	T cell receptor beta variable 10	2.1
17463451	Gm26656	predicted gene, 26656 [Source:MGI Symbol;Acc:MGI:5477150]	2.1
17473635	Olfr1350	olfactory receptor 1350	2.1
17486764	Obox6	oocyte specific homeobox 6	2.1
17536759	Itgb1bp2	integrin beta 1 binding protein 2	2.1
17244057	Gas2l3	growth arrest-specific 2 like 3	2.09
17249483	Zcchc10	zinc finger, CCHC domain containing 10	2.09
17305953	Olfr734	olfactory receptor 734	2.09
17306344	Slc7a7	solute carrier family 7 (cationic amino acid transporter, y+ system), member 7	2.09
17310395	Gm5144	predicted gene 5144	2.09
17346894	Lrrc30	leucine rich repeat containing 30	2.09
17373778	Lincred1	long intergenic non-protein coding RNA of erythroid differentiation 1	2.09
17467072	Pde1c	phosphodiesterase 1C	2.09
17484225	4930544L04Rik	RIKEN cDNA 4930544L04 gene [Source:MGI Symbol;Acc:MGI:1926057]	2.09
17550396			2.09
17229851	Casq1	calsequestrin 1	2.08
17303442	Oit1	oncoprotein induced transcript 1	2.08
17412045			2.08
17424384	Gm13299	predicted gene 13299 [Source:MGI Symbol;Acc:MGI:3701133]	2.08
17527914	4933433G08Rik	RIKEN cDNA 4933433G08 gene	2.08
17538437	Gm24627	predicted gene, 24627 [Source:MGI Symbol;Acc:MGI:5454404]	2.08
17214578	Tmem198	transmembrane protein 198	2.07
17410127	Gm15551	predicted gene 15551	2.07
17222354	4930594C11Rik	G1 to S phase transition pseudogene	2.07
17255556	Mir196a-1	microRNA 196a-1	2.07
17260438	Gm24313	predicted gene, 24313 [Source:MGI Symbol;Acc:MGI:5454090]	2.07
17267430	Gdpd1	glycerophosphodiester phosphodiesterase domain containing 1	2.07
17269229	Krtap4-8	keratin associated protein 4-8	2.07
17292477	Cenpp	centromere protein P	2.07
17317393	Fam84b	family with sequence similarity 84, member B	2.07
17329475	AU015336	expressed sequence AU015336 [Source:MGI Symbol;Acc:MGI:2146536]	2.07
17338355	Gm15556	predicted gene 15556 [Source:MGI Symbol;Acc:MGI:3783005]	2.07
17377258	A930019D19Rik	RIKEN cDNA A930019D19 gene	2.07
17443717	Smok3a; Smok3b	sperm motility kinase 3A; sperm motility kinase 3B	2.07
17455652	Gm7682	predicted gene 7682	2.07
17480030	Rab38	RAB38, member RAS oncogene family	2.07
17486912			2.07
17500242	1700047A11Rik	RIKEN cDNA 1700047A11 gene	2.07
17501288	2500002B13Rik	RIKEN cDNA 2500002B13 gene	2.07
17548904	Gm6489	predicted gene 6489 [Source:MGI Symbol;Acc:MGI:3646684]	2.07

17549260			2.07
17219643	Aim2	absent in melanoma 2	2.06
17277161	Acot5	acyl-CoA thioesterase 5	2.06
17311789	Rnf139	ring finger protein 139	2.06
17349902	Pcdhb11	protocadherin beta 11	2.06
17377210	Cfap61	cilia and flagella associated protein 61	2.06
17393095	Sun5	Sad1 and UNC84 domain containing 5	2.06
17417985	Foxo6os	forkhead box O6, opposite strand	2.06
17434864	Speer8-ps1	spermatogenesis associated glutamate (E)-rich protein 8, pseudogene 1	2.06
17438464	Thegl	theg spermatid protein like	2.06
17508986	Triml1	tripartite motif family-like 1	2.06
17512888	Gm26832	predicted gene, 26832	2.06
17515402	Cnn1	calponin 1	2.06
17224724	BC035947	cDNA sequence BC035947	2.05
17247494			2.05
17248589	Gm12148	predicted gene 12148 [Source:MGI Symbol;Acc:MGI:3649953]	2.05
17258664	Mfsd11	major facilitator superfamily domain containing 11	2.05
17261177	LOC102636777; Gm12665	uncharacterized LOC102636777; predicted gene 12665 [Source:MGI Symbol;Acc:MGI:3652165]	2.05
17272555	Tmc6	transmembrane channel-like gene family 6	2.05
17301232	Gm17232	predicted gene 17232	2.05
17327714	4930455F16Rik	RIKEN cDNA 4930455F16 gene	2.05
17327862	12-Sep	septin 12	2.05
17368401	Stkld1	serine/threonine kinase-like domain containing 1	2.05
17373501	1700029I15Rik	RIKEN cDNA 1700029I15 gene	2.05
17397746	Gm22269	predicted gene, 22269 [Source:MGI Symbol;Acc:MGI:5452046]	2.05
17415424	Gm12637	predicted gene 12637 [Source:MGI Symbol;Acc:MGI:3650764]	2.05
17422546	Cfap74; C030017K20Rik	cilia and flagella associated protein 74; RIKEN cDNA C030017K20 gene	2.05
17458605	Hoxaas3	Hoxa cluster antisense RNA 3	2.05
17460099	Vax2	ventral anterior homeobox 2	2.05
17510264	Haus8	4HAUS augmin-like complex, subunit 8	2.04
17258155	Otop2	otopetrin 2	2.04
17264102	Hs3st3b1	heparan sulfate (glucosamine) 3-O-sulfotransferase 3B1	2.04
17294694			2.04
17373513	Gm13814	predicted gene 13814	2.04
17392652	3300002I08Rik	RIKEN cDNA 3300002I08 gene	2.04
17400732	Pdzk1	PDZ domain containing 1	2.04
17414009	Msantd3	Myb/SANT-like DNA-binding domain containing 3	2.04
17420113	Htr1d	5-hydroxytryptamine (serotonin) receptor 1D	2.04
17433305	1700045H11Rik	RIKEN cDNA 1700045H11 gene	2.04
17492385	Kif7	kinesin family member 7	2.04
17493390	Gm15413	predicted gene 15413	2.04
17548711			2.04
17392317	Pcsk2os2	proprotein convertase subtilisin/kexin type 2, opposite strand 2	2.03
17232724	4930547M16Rik	RIKEN cDNA 4930547M16 gene [Source:MGI Symbol;Acc:MGI:1922549]	2.03
17250392	Myo15	myosin XV	2.03
17274775	Lamb1	laminin B1	2.03
17373943	Pax6	paired box 6	2.03
17398338	Fstl5	follistatin-like 5	2.03
17414173	Nipsnap3b	nipsnap homolog 3B (C. elegans)	2.03
17438597	Gm24524	predicted gene, 24524 [Source:MGI Symbol;Acc:MGI:5454301]	2.03
17453647	Srrm3os	serine/arginine repetitive matrix 3, opposite strand [Source:MGI Symbol;Acc:MGI:1918907]	2.03
17506353	Gm22	predicted gene 22	2.02
17237599	1700064J06Rik	RIKEN cDNA 1700064J06 gene	2.02
17263223	Obscn	obscurin, cytoskeletal calmodulin and titin-interacting RhoGEF	2.02
17284607	Ighv1-62-2; Tnpo3	immunoglobulin heavy variable 1-62-2; transportin 3	2.02
17284631	Ighv1-71; Tnpo3	immunoglobulin heavy variable 1-71; transportin 3	2.02
17355579	Slc14a2	solute carrier family 14 (urea transporter), member 2	2.02
17404941	5430434I15Rik	RIKEN cDNA 5430434I15 gene	2.02
17405352			2.02
17443024	Tyw1	tRNA-yW synthesizing protein 1 homolog (S. cerevisiae)	2.02
17334052	Vmn2r116	vomeronasal 2, receptor 116	2.01
17344331	Gm11131	predicted gene 11131 [Source:MGI Symbol;Acc:MGI:3779386]	2.01

17368286	Gm13562	predicted gene 13562	2.01
17449437	1700066N21Rik	RIKEN cDNA 1700066N21 gene	2.01
17458544	C530044C16Rik	RIKEN cDNA C530044C16 gene	2.01
17410921	Clca4a	chloride channel accessory 4A	-2.09
17238878	Syne1	spectrin repeat containing, nuclear envelope 1	-6.44
17479485			-7.44
17278761	AF357425	snoRNA AF357425	-22.59
17541917	Snord61	small nucleolar RNA, C/D box 61	-29.61

Table S6: Transcription factors affected by Western diet and LDL-R -/- in hippocampal microvascular endothelium.

M WT WD vs WT CD		M LDL-R -/- CD vs WT CD		M LDL-R -/- WD vs WT CD	
Network	***p-Value***	***Network***	***p-Value***	***Network***	***p-Value***
GATA-2	7.64E-14	ETS1	0	ETS1	0
ETS1	7.64E-14	FOXP3	0	FOXP3	0
FOXP3	7.64E-14	E2F1	0	E2F1	0
GABP alpha	7.64E-14	Oct-3/4	0	Oct-3/4	0
Oct-3/4	2.55E-09	GABP alpha	0	CREB1	0
c-Myc	2.55E-09	CREB1	0	GABP alpha	0
E2F1	2.55E-09	SOX2	0	SOX2	0
CREB1	2.55E-09	NANOG	0	NANOG	0
YY1	2.55E-09	c-Myc	0	c-Myc	0
SP1	0.000063	GLIS3	0	GLIS3	0
SOX2	0.000063	GATA-2	0	N-Myc	1.85E-277
ESR2	0.000063	ZFX	0	HNF4-alpha	8.79E-250
HNF4-alpha	0.000063	HNF4-alpha	0	KLF4	3.01E-242
SMAD1	0.000063	KLF4	0	GATA-2	3.01E-242
BMAL1	0.000063	ESR1 (nuclear)	1.13E-300	ZFX	9.73E-240
POU3F2 (BRN2)	0.000063	LBP9	8.74E-296	p53	1.08E-224
PU.1	0.000063	N-Myc	1.87E-288	Androgen receptor	3.52E-217
AP-1	0.000063	GATA-1	1.87E-288	SP1	3.52E-217
SRF	0.000063	SP1	3.95E-281	RelA (p65 NF-kB subunit)	3.59E-207
AML1 (RUNX1)	0.000063	E2F4	1.09E-278	ESR1 (nuclear)	1.13E-199
PPAR-gamma	0.000063	p53	7.02E-252	LBP9	1.12E-194
HIF1A	0.000063	Androgen receptor	1.02E-239	E2F4	1.08E-184
GLIS3	0.000063	RelA (p65 NF-kB subunit)	2.75E-237	GATA-1	8.81E-160
AP-2A	0.000063	GATA-3	7.42E-235	STAT3	8.29E-155
LBP9	0.000063	YY1	1.99E-232	TCF7L2 (TCF4)	5.54E-130
KLF4	0.000063	STAT3	1.44E-227	TCF7L1 (TCF3)	5.54E-130
ESR1 (nuclear)	0.000063	GCR	1.38E-210	GATA-3	5.54E-130
c-Jun/c-Fos	0.000063	HIF1A	1.25E-193	HIF1A	1.67E-127
PEA3	0.000063	c-Jun	2.26E-186	c-Jun	1.67E-127
Androgen receptor	0.000063	HNF3-beta	5.93E-184	HNF3-beta	4.5E-120

Table S7: Effect of the Western diet on the expression of microRNAs (miRNAs) in male hippocampal microvessels.

MiRNAs	WT WD vs WT CD	LDL-R -/- CD vs WT CD	LDL-R -/- WD vs WT CD
Mir1954	3.28		
Mir687		36.93	
Mir882		35.17	
Mir466n		32.87	
Mir382		29.58	
Mir1938		24.76	
Gm24098		23.99	
Gm25381		23.99	
Mir1188		18.6	
Mir692-3		17.54	20.29
Mir692-2		17.54	20.29
Mir1954		15.13	20.12
Mir678		13.64	6.01
Mir673		11.7	81.06
Mir6418		10.54	
Mir377		9.62	
Mir1955		9.62	
Mir703		8.96	7.27
Mir466d		7.15	
Mir466j		5.82	
Mir761		5.22	
Mir466h		4.95	5.7
Mir210		4.3	11.24
Mir204		4.11	
Mir344d-2		3.07	2.82
Mir9-2		2.87	
Mir5097		2.84	
Mir466f-4		2.55	
Mir375		2.47	3.06
Mir34c		2.3	
Mir343		2.28	
Mir1912		2.11	
Mir21a		2.04	
Gm23134		16.67	16.89
Gm25559		11.52	
Gm25817		10.11	52.55
Gm24766		10.11	52.55
Gm22932		9.59	
Gm24813		4.47	2.84
Gm25068		4	
Gm22323		2.75	3.36
Gm23931		2.75	3.36
Gm25992		2.08	
Gm26154		23.81	
Mir679			113.69
Mir30d			69.19
Mir669n			67.85
Mir692-1			21.43
Gm15590			20.29
Gm22774			20.29
Mir1192			15.06
Mir574			7.94
Mir6990			6.36
Mir28b			4.87
Mir5109			4.33
Mir378d			4.28
Mir1198			3.86
Mir1199			3.76
Mir486			2.96
Mir196a-1			2.07
Gm24328			23.68
Gm22757			17.11
Gm22759			11.66
Gm24327			4.73
Gm24096			3.22
Gm24627			2.08
Gm24541			-7.44
